# New Insights into the Genetic Control of Gene Expression using a Bayesian Multi-tissue Approach

**DOI:** 10.1371/journal.pcbi.1000737

**Published:** 2010-04-08

**Authors:** Enrico Petretto, Leonardo Bottolo, Sarah R. Langley, Matthias Heinig, Chris McDermott-Roe, Rizwan Sarwar, Michal Pravenec, Norbert Hübner, Timothy J. Aitman, Stuart A. Cook, Sylvia Richardson

**Affiliations:** 1 Medical Research Council Clinical Sciences Centre, Faculty of Medicine, Imperial College London, London, United Kingdom; 2 Department of Epidemiology and Biostatistics, Faculty of Medicine, Imperial College, London, United Kingdom; 3 Max-Delbrück Center for Molecular Medicine, Berlin, Germany; 4 Institute of Physiology, Czech Academy of Sciences and Centre for Applied Genomics, Prague, Czech Republic; 5 Charles University in Prague, Institute of Biology and Medical Genetics of the First Faculty of Medicine and General Teaching Hospital, Prague, Czech Republic; 6 Section of Molecular Genetics and Rheumatology, Division and Faculty of Medicine, Imperial College, London, United Kingdom; 7 National Heart and Lung Institute, Imperial College, London, United Kingdom; Cornell University, United States of America

## Abstract

The majority of expression quantitative trait locus (eQTL) studies have been carried out in single tissues or cell types, using methods that ignore information shared across tissues. Although global analysis of RNA expression in multiple tissues is now feasible, few integrated statistical frameworks for joint analysis of gene expression across tissues combined with simultaneous analysis of multiple genetic variants have been developed to date. Here, we propose Sparse Bayesian Regression models for mapping eQTLs within individual tissues and simultaneously across tissues. Testing these on a set of 2,000 genes in four tissues, we demonstrate that our methods are more powerful than traditional approaches in revealing the true complexity of the eQTL landscape at the systems-level. Highlighting the power of our method, we identified a two-eQTL model (*cis*/*trans*) for the *Hopx* gene that was experimentally validated and was not detected by conventional approaches. We showed common genetic regulation of gene expression across four tissues for ∼27% of transcripts, providing >5 fold increase in eQTLs detection when compared with single tissue analyses at 5% FDR level. These findings provide a new opportunity to uncover complex genetic regulatory mechanisms controlling global gene expression while the generality of our modelling approach makes it adaptable to other model systems and humans, with broad application to analysis of multiple intermediate and whole-body phenotypes.

## Author Summary

Integrated analysis of genome-wide genetic polymorphisms and gene expression profiles from different tissues or cell types has been highly successful in identifying genes modulating complex phenotypes in animal models and humans. However, an important limitation of the current approaches consists in their sole application to individual tissues, thus ignoring information shared across different tissues. To uncover complex genetic regulatory mechanisms controlling gene expression at the whole organism's level, it is essential to develop appropriate analytical methods for the analysis of genome-wide genetic polymorphisms and gene expression profiles simultaneously in multiple tissues. This paper presents a novel, fully integrated Bayesian approach for mapping the genetic components of gene expression within and across multiple tissues. In addition to increased power and enhanced mapping resolution when compared with traditional approaches, our model directly provides information on potential systemic effects on transcriptional profiles and co-existing local (*cis*) and distant (*trans*) genetic control of gene expression. We also discuss the possibility to extend our approach for the analysis of different phenotypes, and other study designs, thus providing an integrated computational tool to explore the genetic control underlying transcriptional regulation at the systems-level, beyond the single tissue resolution.

## Introduction

### Background

A number of integrated transcriptional profiling and linkage mapping studies have been published to date [1]–[8], however most of these studies were restricted to analysis in single tissues or cell types. Even when expression profiles are available from multiple tissues, expression QTL (eQTL) mapping is usually carried out at the level of the individual tissue and the lists of significant eQTLs are subsequently compared across experiments [3],[8],[9]. One limitation of this approach is that different false positive (and/or false negative) rates across studies inflate the discrepancies between the lists of eQTLs [10]. In addition, intersecting eQTL lists with similar False Discovery Rates (FDR) is likely to be a conservative approach and is potentially affected by variability between tissues [11]. A number of studies have investigated whether the *cis*- and/or *trans*-acting genetic control of gene expression is conserved across tissues, i.e. whether there is evidence of tissue-consistent eQTLs. By a slight abuse of terminology, here, we refer for simplicity to tissue-consistent eQTL as “pleiotropic eQTL”, i.e. when an eQTL for the same probe set expression is detected across multiple tissues (not necessarily exerting multiple cellular functions). eQTL studies in the rat [3],[11], mouse [12] and in humans [8] have shown that detection of eQTLs with a systemic effect (i.e., detected across multiple tissues) is strongly biased towards *cis*-eQTLs. This is likely a consequence of their strong genetic effects, levels of expression, high heritability or a combination of these factors, but could also result from a lack of power to detect smaller effects, typically *trans*-eQTLs. *Cis*-acting genetic variation can have important pathophysiological consequences at the level of the whole organism [5],[8],[13], likely reflecting modifications of key regulatory functions across tissues and cell-types. However, studies in plants have shown that *cis*-eQTLs can also exhibit strong tissue-specific dependency, and polymorphisms in *cis*-regulatory regions may affect gene transcription exclusively in a few crucial cell types [14],[15].

Identification of *trans*-eQTLs within and across tissues or cell lines is statistically challenging, and it is plausible that the relative paucity of shared *trans*-regulatory effects discovered to date is mainly due to their small genetic effect and higher FDR [5],[11],[16]. A review of the eQTL literature data reveals that many observations of *trans*-regulated gene expression are often contradictory [10], and detection of *trans*-eQTL hotspots can be affected by the permutation strategy used to assess their statistical significance [17]. Tissue-specific transcriptional regulation has been reported for differentially expressed genes and for regulatory genetic hotspots [18] or region-specific regulatory networks and genes regulated by *trans*-acting elements [19]. However, using inbred mouse lines, Bystrykh *et al.* [9] observed a substantial proportion of *trans*-eQTLs with identical genomic location in brain and stem cell tissues in the same animals. These discrepant observations highlight the uncertainty concerning cross-tissue conservation of *cis* and *trans*-acting genetic regulation.

### State of the art statistical methods

Although global analysis of mRNA expression in multiple tissues in now feasible [20],[21], few methods have addressed the problem of jointly performing an analysis of gene expression across tissues combined with multivariate analysis of a large number of genetic control points. Statistical methods for multiple QTL analyses have followed current developments of sparse regression methods designed to address the large *p*, small *n* paradigm, i.e., set-ups where the number of potential covariates (here, the genetic markers) is (much) larger than the number of available samples. In this context, two families of methods can broadly be distinguished: regularised multivariate regression approaches such as the Lasso [22], where the residual sum of squares is penalised and regression coefficients are shrunk towards zero, or methods using a variable selection formulation, typically implemented in a Bayesian framework. Regularised regressions are focussed on delivering overall good predictive ability rather than interpretability of the effect of a few key regressors, whereas variable selection methods are constructed to explore a large model space, seeking a set of well supported models, each including only a small number of interpretable regressors. In the eQTL context, regularisation methods have been proposed for single [23] and multiple phenotypes [24]. However, interpretability of the genetic effects is important as well as an adequate characterisation of uncertainty, and the Bayesian variable selection (BVS) approach that we and others [25]–[27] have adopted offers additional insights.

In this paper we have implemented a new Bayesian variable selection method for multivariate mapping of single or multiple outcomes, and show an application to uncover simultaneous *cis* and *trans*-regulation of gene expression at the level of individual tissues as well as across tissues. We show that by implementing a computationally challenging multi-locus strategy, our model can identify substantially more *cis*- and *trans*-effects than commonly used single marker eQTL methods for the same FDR level and that it permits efficient identification of genetic regulation across multiple tissue types. These biological findings are complemented by a simulation study where our method is compared to classical and a recently proposed multi-locus penalised regression method and shown to have increased power.

## Results

We used Sparse Bayesian Regression (SBR) and Sparse Bayesian Multiple Regression (SBMR) models to identify genetic control points of gene expression, which are common across or specific to four rat tissues. To demonstrate the power of this approach, we selected a subset of 2,000 probe sets that show the highest variation in gene expression in the BXH/HXB RI strains [3] jointly across fat, kidney, adrenal and left ventricle (heart hereafter) tissues (see Materials and Methods). This selection of probe sets is not biased towards highly heritable transcripts (Table S1), and the observed correlations in mRNA levels resemble the correlation structure in the whole set of transcripts (Figure S1). We carried out multi-locus eQTL mapping *i*) within each tissue using SBR models and *ii*) in all tissues simultaneously using the SBMR model (Figure S2). In the latter analysis, mRNA levels measured within each tissue were assembled in a single dataset and treated as multiple responses of the same feature (see Materials and Methods). The SBMR identifies the best combination of markers that jointly predict the responses, thus representing a pleiotropic model for predicting variation in gene expression in all tissues. Results from both SBR analysis were compared with eQTL analysis using QTL Reaper which based on the Haley-Knott regression [3],[11], and with a two-stage Sequential Search Method (SSM) of pairs of significant eQTLs, following Storey's approach [28], that was adapted to map one or more eQTLs using a purely additive eQTL model (Text S1). These methods have been widely used to map genome-wide eQTLs in several systems [3], [4], [9], [11], [13], [28]–[30] and because of their wide applicability they represent a useful benchmark for our approach. The results of the SBMR approach were compared with the Hotelling's *T* ^2^-test for mapping eQTL across multiple tissues (see Materials and Methods) and with the eQTLs identified by intersecting eQTL lists from single tissue analyses.

### Single tissue analysis

We first investigated the distribution of the size of the eQTL lists associated with the best SBR model visited, for the transcripts that were below the 5% FDR using Jeffreys' scale of evidence (see Materials and Methods). Consistently across all tissues, ∼16% of all probe sets were under genetic control by one eQTL, although for a small proportion of probe sets (∼3%) multiple control points were detected (Table 1). As expected, adopting more conservative FDR levels the proportion of probe sets with multiple eQTLs decreases significantly (Table S2). The SBR model identified a similar number (or more) of eQTLs compared with the SSM approach, whereas it yielded substantially more eQTLs than QTL Reaper (∼2 fold increase) (Table S3). All methods identified a larger proportion of *cis* than *trans*-eQTLs to a varying degree, with enrichment for *cis*-eQTLs that were commonly detected by all methods (from 72% to 78% across tissues).

**Table 1:** Number of probe sets found to be under genetic control in the SBR and SBMR analyses (FDR <5%).

*Analysis*	no eQTL*	1 eQTL¶	2 eQTLs¶	≥3 eQTLs¶
SBR in fat	1634 (81.7%)	311 (15.6%)	43 (2.2%)	12 (0.6%)
SBR in kidney	1627 (81.4%)	323 (16.2%)	38 (1.9%)	12 (0.6%)
SBR in adrenal	1649 (82.5%)	301 (15.1%)	40 (2.0%)	10 (0.5%)
SBR in heart	1607 (80.4%)	345 (17.3%)	39 (2.0%)	9 (0.5%)
SBMR in all tissues	1469 (73.5%)	275 (13.8%)	93 (4.7%)	163 (8.2%)

*We used “no eQTL” to identify probe sets whose best model visited was the null model (i.e., no evidence of genetic control) or when the best model visited with genetic control was not significant at FDR <5% (see Materials and Methods).

**¶:** For the probe sets that are under genetic control the number of probe sets with one, two or at least 3 distinct eQTLs is indicated. Polygenic models (2 eQTLs and ≥3 eQTLs) are indicative of two or more distinct eQTLs (for the same probe set) that are located at least 10 cM far apart. Percentages were calculated with respect of the set of 2,000 transcripts considered in this study.

The SBR outperformed both QTL Reaper and SSM approaches in detecting complex genetic regulation by two or more eQTLs. While QTL Reaper and SSM found no polygenic control in any tissue at 5% FDR, the SBR model revealed that ∼12% of the probe sets that were found to be under genetic control across tissues mapped to two or more distinct eQTLs, delineating a set of 140 polygenic expression traits (Table S4). Similarly, the full two-stage SSM that accounts for epistatic interaction between the primary and secondary locus [28] found no significant polygenic regulation (data not shown). This indicates that while the number of observations in this dataset is relatively small, when compared with either QTL Reaper or SSM approaches, the multi-locus approach implemented by SBR model offers a significant gain in power for eQTL detection, with increased sensitivity to identify polygenic effects at low FDR levels. A detailed comparison between the single tissue analyses using the different approaches is reported in Text S1.

### Multiple tissues analysis

Thresholding the Jeffreys' scale of evidence to control the FDR at 5% level, the SBMR model identified 531 transcripts (∼27% of the total) under common genetic regulation in all tissues. We showed evidence of polygenic control by two or more distinct eQTLs for a significant proportion of probe sets (13%) (Table 1), and this fraction remains substantial, albeit decreasing, when more conservative FDR thresholds are considered (Table S2). This reflects the high sensitivity of the SBMR approach to identify potential pleiotropic loci even when their individual effect within each tissue is marginally weak. Although we specified priors on the model size to penalize highly polygenic models, the evidence provided by the data supports common genetic regulation for SBMR models with a high number of eQTLs (Figure S3).

**Figure 1:**
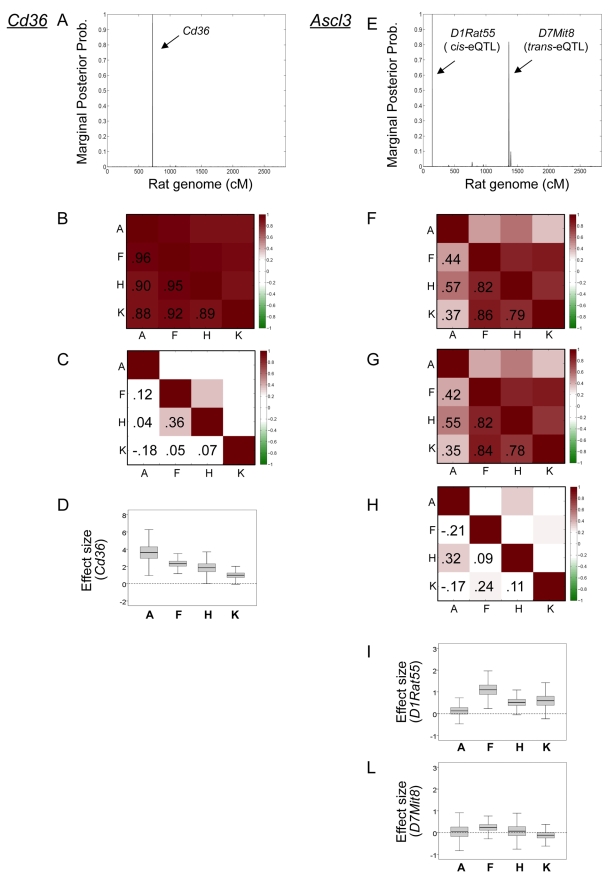
Evidence of pleiotropic eQTLs detected by the SBMR model. (A, E) For each gene, the set of markers associated with high marginal posterior probability of inclusion corresponds to the filtered best model found by SBMR, showing monogenic control for *Cd36* gene (marker *Cd36*) and polygenic control for *Ascl3* gene (markers *D1Rat55* and *D7Mit8*). The marginal probability of inclusion is calculated conditionally on all visited models whose 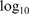 Bayes Factor is above the calibrated threshold at 5% FDR level (see Materials and Methods). (B, C, F, G, H) Systemic effects of pleiotropic eQTLs detected by the SBMR model. For each gene we report the raw empirical correlation of gene expression across four tissues and the posterior mean of the residual correlation matrix. Posterior correlations in panels C, H were simulated conditionally on the filtered best model that coincide with the noticeable effects (see Materials and Methods): marker *Cd36* for *Cd36* gene and markers *D1Rat55* and *D7Mit8* for *Ascl3* gene, respectively. In panel G, the posterior correlations were generated conditionally on the *cis*-eQTL only (marker *D1Rat55*). The size of the correlation is colour coded and reported in each graph. (D, I, L) Box-plots of the posterior density of the effect size (see Text S1) for the eQTLs with noticeable effect are reported for each tissue. These illustrate the tissue-specific contribution provided by each eQTL to the pleiotropic effect. Tissues: A, adrenal; F, fat; H, heart; K, kidney.

A key aspect of the SBMR approach is that it exploits additional information provided by the covariance structure between tissues to find a set of parsimonious models that jointly predict gene expression levels in all tissues. For illustration, Figure 1A–E shows contrasting case examples for *Cd36* and *Ascl3* genes, where the SBMR confirmed shared genetic effects due to a single *cis*-eQTL for *Cd36* (marker *Cd36*) and indentifies a new *cis*- and *trans*-eQTL genetic model for *Ascl3* (markers *D1Rat55* and *D7Mit8*, respectively). The Hotelling's *T* ^2^-test found common genetic regulation for *Cd36* gene, while it indentified only the *cis*-eQTL for *Ascl3* but failed to detect the secondary *trans*-acting locus at the 5% FDR level (Figure S4). For comparison with the single tissue analyses using SBR, the *cis*-effect for *Ascl3* is seen in fat, kidney and heart, while the *trans*-signal is detectable only in fat and heart (Figure S5). Similarly, both QTL Reaper and SSM failed to detect common genetic regulation in *cis* or *trans* across tissues for the *Ascl3* gene (Figure S4).

The proposed SBMR model directly provides information on potential systemic effects of the eQTL(s). To assess the extent by which the detected common eQTLs explain the correlation in gene expression across tissues, we calculated the raw empirical correlation matrix and the posterior mean of the residual correlation matrix given the putative eQTL markers (see Text S1). For both *Cd36* and *Ascl3* genes, Figure 1 shows that the SBMR approach pinpoints genetic regulators that explain the majority of the correlation structure between tissues as the off-diagonal residual correlations are considerably smaller (Figure 1, panels B, C and F, G, H). The probe set (1386901_at) representing *Cd36* gene is derived from sequence in the 3′ untranslated region, that is constitutively deleted from the SHR genome [31], indicating a systemic effect of this *cis*-eQTL as shown by the posterior mean of the residual correlation matrix (Figure 1C). For the *Ascl3* gene, the *cis*-eQTL alone (marker *D1Rat55*) is not sufficient to explain the pattern of correlation leading to substantial residual correlation (Figure 1G), whereas inclusion of the *trans*-locus (*D7Mit8*) significantly decreased the residual correlation in gene expression across tissues (Figure 1H). This suggests that both the *cis*- and *trans*-eQTLs might have a tissue-consistent pleiotropic effect on *Ascl3* expression, similarly to *Cd36*. In addition, within the SBMR model we investigated the effect size of the eQTLs within each tissue by simulating their posterior density in a post processing analysis (see Materials and Methods). For each gene, we report the distribution of the eQTL effect size across tissues showing marked effects for the *cis*-eQTLs for both *Cd36* and *Ascl3* genes (Figure 1D and Figure 1I, respectively). In the latter case, despite the smaller effect size for the *trans*-acting eQTL (Figure 1L), its influence on expression in fat and heart tissues is visible. Taken together, these findings provide evidence of common genetic regulation by one or two loci in the case of *Cd36* and *Ascl3* genes, respectively. Using post-processing results we were able to uncover the tissue-specific contribution of each eQTL to the pleiotropic model. These illustrative examples show how the SBMR approach makes use of the information shared across tissues by joint modelling of mRNA levels to identify common genetic control points of gene expression across tissues.

### Comparison between multiple and single tissues analyses

We investigated whether the common eQTLs mapped within each tissue by the SBR model were detected in the SBMR analysis. Ninety-three transcripts showed genetic regulation by the same eQTL that was independently detected in all tissues by SBR (FDR <5%) (Table S5). In contrast, at similar FDR levels, the SBMR approach identified 531 probe sets under genetic regulation in all tissues, yielding >5 fold increase in the number of common eQTLs when compared with the SBR (Table 1). When contrasting the SBMR approach with QTL Reaper and the SSM, which detected 50 and 59 shared eQTLs, respectively, we found ∼10 times more shared eQTLs at 5% FDR. The SBMR model was also more powerful in detecting shared *trans*-acting regulation when compared to SBR (or both QTL Reaper and SSM methods). While the SBR approach identified only five transcripts with common regulation by the same *trans*-eQTL in all tissues, SBMR yielded 42 models (2%) with one *trans*-acting eQTL, 147 models (7%) with *trans*-acting eQTLs that are observed in combination with a *cis*-eQTL and 95 models (∼5%) with multiple *trans*-eQTLs for the same transcript. This suggests that exploiting the dependence between gene expression levels among tissues greatly enhance identification of common *trans*-regulators that can be missed when eQTLs are mapped separately within individual tissues.

### Comparison with other multivariate approaches

Both SBMR and Hotelling's *T* ^2^-test approaches outperformed the single tissue analyses and identified a common set of 373 transcripts with tissue consistent pleiotropic eQTLs, where 277 were *cis*-eQTL genes (Table S6). This set of eQTLs can be considered as common regulatory control points of gene expression across all tissues that have been replicated using two independent statistical approaches. We compared the performance of the SBMR approach with that of the Hotelling's *T* ^2^-test and showed that our method found significantly more polygenic regulation, accounting for ∼13% of all transcripts, as compared with 3% found by the Hotelling's *T* ^2^-test. These analyses suggest that while both approaches agree in finding common *cis*-regulation, the SBMR model had increased power to discover complex genetic regulation of gene expression across tissues when compared with a traditional approach based on analyzing each marker separately (see Text S1 for detailed comparisons). While this increased power could be expected in principle from the use of a multivariate method, we shows that the SBMR algorithm succeeds in exploring effectively the vast space of possible multi-locus models, which is a very challenging task.

**Figure 2:**
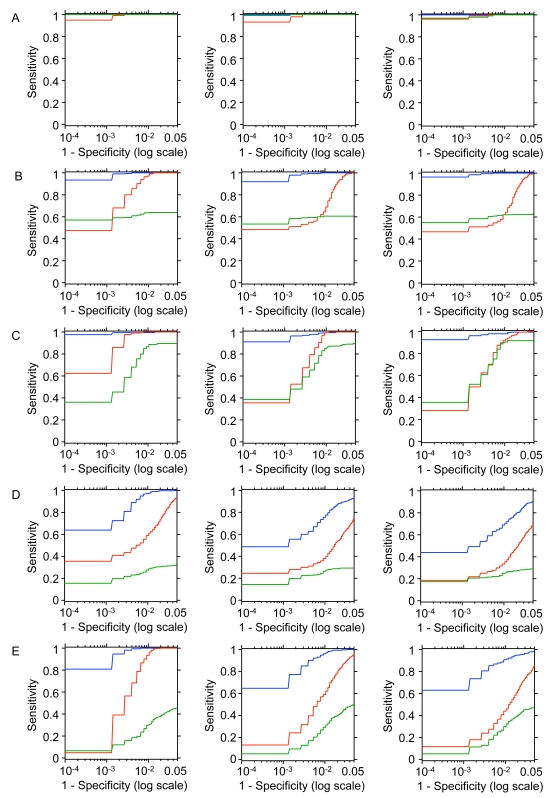
Sensitivity and specificity of SBMR and alternative approaches. Log-scale Receiver Operating Characteristic (ROC) curves of SMBR (blue), Hotelling's 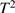-test (red) and GFlasso (green) methods, using simulated data generated under five different scenarios. The scenarios for the pleiotropic eQTL are as follows: (A) one *cis*-eQTL; (B) one *cis*- and one *trans*-eQTLs; (C) two *trans*-eQTLs; (D) one *cis*-eQTLs and four *trans*-eQTLs and (E) four *trans*-eQTLs. In each case we simulated strong (left panel), medium (central panel) and weak (right panel) correlation pattern among gene expression traits (see Text S1 for details).

In addition, we carried out a simulation study to investigate the power of our approach as compared with the Hotelling's 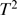-test and a recently proposed generalised Lasso-type algorithm and associated software, the GFlasso [24], which also considers multi-locus models on the full set of markers and is specifically designed to borrow information across correlated phenotypes. In all simulated cases (see Text S1 for details), the SBMR outperformed both the Hotelling's 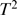-test and the GFlasso algorithm, in particular in the detection of polygenic control by strong and weak eQTL effects (i.e., one *cis*-QTL and multiple *trans*-QTLs) or several weak effects (i.e., *trans*-QTLs), Figure 2 and Text S1.

### Validation of eQTL linkages

To validate eQTL linkages detected by microarray using Bayesian model approaches, we measured mRNA abundance in the BXH/HXB RI strains by quantitative RT-PCR (qRT-PCR) for *cis*- and *trans*-acting eQTLs, including complex polygenic effects. We confirmed eQTL findings for strong *cis*-acting linkages, such as *EndoG* and *Card9* (FDR <5%) (Figure S6), as well as for weaker *trans*-acting linkages that were observed for two transcription factors, *Stat4* and *Irf7* (FDR <5%) (Figure S7). Although traditional eQTL mapping approaches identified the *trans*-linkage for *Stat4*, they failed to detect the *trans*-eQTL for *Irf7* at the FDR cut-off of 5%. By contrast, our Bayesian approach identified these *trans*-eQTLs with high confidence (FDR <5%, for both genes), indicating that the data provide convincing evidence for these eQTLs (i.e., large Bayes Factor [32]) despite a model formulation that gives *a priori* a larger weight to the null model (i.e., no genetic control) (see Materials and Methods).

**Figure 3:**
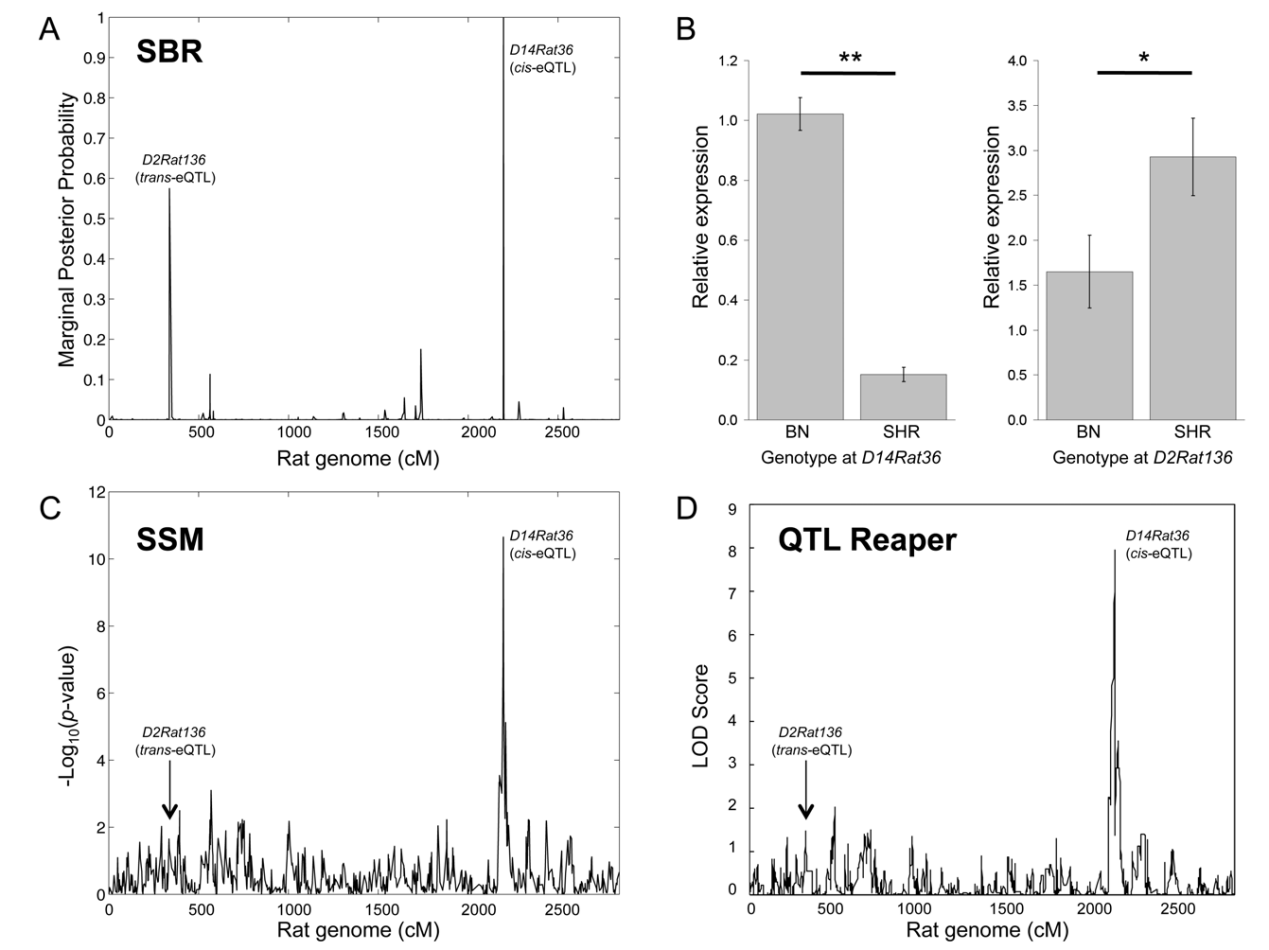
Validation of polygenic regulation for *Hopx*. The filtered best model for the regulation of *Hopx* indicates polygenic control of gene expression by two co-existing eQTLs, *D14Rat36* and *D2Rat136*. The marginal posterior probability of inclusion for the *cis*- (*D14Rat36*) and *trans*-eQTL (*D2Rat136*) is reported in panel (A). RT-PCR data showing relative *Hopx* expression in the BXH/HXB RI strains by BN and SHR genotypes at peak markers *D14Rat36* (left panel) and *D2Rat136* (right panel), (B). The *cis*-eQTL is identified by all methods (SSM: FDR <5%; QTL Reaper: genome-wide corrected *p*-value, 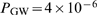, FDR <5%), while the weaker *trans*-eQTL at marker *D2Rat136* (indicated by an arrow) is not significantly detected by either the SSM (panel C) or QTL Reaper (panel D) methods. This shows the power of the SBR model to identify both small (*trans*-acting) and big (*cis*-acting) genetic effects that can simultaneously determine variation in gene expression. Relative expressions are reported as mean ± sem. (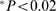, 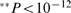).

As a further example to highlight the power of our method when compared to other approaches, we also validated polygenic regulation for the *Hopx* gene, where the best model indicated two coexisting eQTLs at markers *D14Rat362* (*cis*) and *D2Rat136* (*trans*), respectively (Figure 3A–B). Notably, both QTL Reaper and the SSM identified the *cis*-eQTL at marker *D14Rat362* but failed to detect significant *trans*-regulation for *Hopx* expression (Figure 3C–D). This may reflect the strong *cis*-acting regulation of *Hopx* (*D14Rat362*, fold change = 4.4, *P* = 7×10^−14^, by RT-PCR), which masks the weaker but still detectable *trans*-eQTL linkage on chromosome 2 (*D2Rat136*, fold change = 1.6, *P* = 0.02, by RT-PCR) (Figure 3). However, even when we accounted for the effect of the *cis*-eQTL by using composite interval mapping, the *trans*-eQTL at *D2Rat136* was not significantly detected by standard methods (genome-wide significance, *P*_GW_ = 0.316). These results indicate that our Sparse Bayesian model is more powerful for identifying polygenic control relative to the other methods, and that both *cis* and *trans* regulation can simultaneously contribute to variation in gene expression levels, emphasizing the complex nature of gene expression regulation in this system.

## Discussion

We have shown that our Sparse Bayesian Regression models coupled with an efficient computational algorithm (Evolutionary Stochastic Search, ESS hereafter) provide significant advantages over other methods in eQTL mapping within and across multiple tissues. A key feature of the proposed approach is its ability to uncover polygenic regulation of gene expression, with greater power to identify secondary *trans*-eQTLs than traditional methods. Notably, while the standard univariate approaches tested found no significant polygenic control in any tissue, the SBR model revealed a set of 140 probe sets that mapped to two or more distinct eQTLs in at least one tissue (Table S4). To illustrate the power of our method for capturing complex genetic regulation of gene expression, we report a new example of co-existing *cis*- and *trans*-acting eQTLs for the *Hopx* gene in the heart, which was not detected by conventional approaches and that we validated experimentally (Figure 3).

We extended the SBR model to accommodate multiple phenotypic responses such as expression profiles in multiple tissues, and showed increased power to discover pleiotropic genetic regulation of gene expression, that was unappreciated by single tissue analyses or other multivariate approaches. We showed that the SBMR model yielded >5 fold increase in the number of common eQTLs when compared with the SBR model. We identified a set of 277 *cis*-eQTLs using SBMR, which was replicated by the Hotelling's *T* ^2^-test analysis, highlighting the increased power provided by multivariate approaches when compared with intersection of lists of eQTLs mapped within individual tissues.

An additional major advantage of the SBMR approach is its ability to assess systemic genetic effects, as illustrated for the *Cd36* [31] and the *Ascl3* genes (Figure 1). In the latter case, we confirmed systemic *cis*-regulation, previously reported in kidney, liver, skeletal muscle, fat [33], and suggest a role for an additional *trans*-locus that explains a substantial amount of the correlation in gene expression between tissues. Detection of systemic genetic effects may shed light on tissues that are biologically active for specific disease processes at the organism level which otherwise would not be appreciated.

For detection of common polygenic and *trans*-acting regulation of gene expression, SBMR outperformed the multivariate Hotelling's *T* ^2^-test. The extra power gained by SMBR in the real and simulated data sets relative to that of the Hotelling's *T* ^2^-test is attributable to its full multivariate modelling of both predictors (markers) and responses (expression profiles in multiple tissues), whereas the Hotelling's *T* ^2^-test is multivariate only on the responses. In addition, our simulations show that SBMR is more competitive than the multivariate Lasso-based algorithm GFlasso [24], which is specifically designed to borrow information across correlated phenotypes (Figure 2). Overall, this highlights the advantage of performing a powerful multivariate analysis of genetic and genomic data to uncover complex regulatory mechanisms at the systems-level.

Computationally, our ESS algorithm implemented for SBR and SBMR is more efficient than other Bayesian variable selection methods since we sample just the vectors of selection indicators (see Materials and Methods), while the remaining parameters (i.e., size of the genetic effects and correlation structure) are analytically integrated out, allowing a fast mixing of the MCMC. When these latter parameters are of interest, they can be simulated in a post processing analysis. Moreover, using multiple chains run in parallel with search moves inspired from genetic algorithms, we significantly improve the exploration of good combinations of markers that predict the variation of gene expression. The software to perform SBR and SBMR analyses is freely available from the authors as a Matlab program, and we have demonstrated that it can scale efficiently to search over tens of thousands of predictors (L. B., S. R., unpublished data).

Our approach is quite flexible and the underlying linear regression model as well as the model search could be extended to handle more complex scenarios, including human data and other genetic study designs. This versatility is currently being implemented in our software, enabling data from different sources to be analysed, for example with applications to gene expression and epigenetic profiles, or to deal with binary outcomes and quantitative predictors in a similar manner, as well as extending the search space to include epistatic interactions within the predictor subsets. One important additional benefit of our Bayesian variable selection approach is that, besides providing a best visited model with a list of eQTLs, it also addresses the inherent uncertainty in finding best predictor subsets. Looking marginally at the role of each marker, we can average over a set of well supported models to assess the overall marginal contribution of each eQTL to explain gene expression variability. Moreover, we can use the same set of models to perform further post-processing analysis, for example to focus on eQTLs with noticeable biological effects in all tissues (see Text S1 for illustrative examples).

In conclusion, we have shown that the SBR and SBMR approaches have distinctive features and perform significantly better than the existing eQTL mapping methods tested. The proposed modelling approaches provide a general and powerful framework for investigating complex genetic regulatory mechanisms controlling gene expression at the systems-level.

## Materials and Methods

Additional technical details on the implementation of the Bayesian model, detailed comparison between methods, illustrative examples and simulations are given in Text S1.

### Datasets

Here we used data previously described by Petretto *et al.* 2006 [11] who measured gene expression levels in four tissues in a panel of 29 rat Recombinant Inbred (RI) strains derived from a cross between the Spontaneously Hypertensive Rat (SHR) and the Brown Norway (BN) strains [3]. We used a panel of 770 non-redundant genetic markers; missing values (accounting for ∼3% of all genotypes) were imputed by interpolating the genotype values between flanking markers [34]. We investigated whether substantial genotype imputation (at least 10% of genotypes of each marker) have an effect on the identified eQTLs and found that imputed genotypes accounted for a small fraction (<10%) of the total number of eQTLs mapped within single tissues or across tissues by SBR and SBMR, respectively. Gene expression measurements were standardized across tissues to reduce potential batch effects, by computing expression summary values using the Robust Multichip Average (RMA) algorithm [35] and pooling together Affymetrix GeneChip data for all tissues. We assume that mRNA levels of each gene measured within each tissue are dependent within each strain and thus can be treated as multiple response of the same feature.

To show the benefit of the proposed statistical method, in this pilot study we analyzed a subset of 2,000 probe sets from the original set of 15,923 that are common in the four tissues. In particular we chose a set of 2,000 probe sets that have the largest variation across tissues, measured as 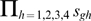, where 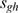 is the standard deviation of the 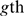 probe set in the 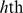 tissue. We investigated if the proposed selection criteria introduce some bias in the between-tissue correlation pattern for each pairs of probe sets: when compared with the whole set of probe sets, the correlation structure shows no evidence of alteration with a slight increment of positive correlation among the selected probe sets (Figure S1).

### Non-Bayesian mapping

*Cis*- and *trans*-eQTLs were mapped using standard regression-based approach (Haley-Knott regression) as implemented in the QTL Reaper program (http://sourceforge.net/projects/qtlreaper/) [29] and using a modified version of the two-stage Sequential Search Method (SSM) for multiple eQTLs [28], without including an additional gene×gene interaction term (Text S1 2.1). For the probe sets that mapped to unique positions in the genome, we determined which eQTLs were regulated in *cis* or in *trans* by defining *cis*-eQTLs as those with a linkage peak within 10 Mbp of the physical location of the probe set [11]. In order to avoid an inflated number of eQTLs, for each probe set we investigated the genetic control point(s) and, within a 5 cM window, we removed redundant eQTLs which may result from linkage of expression values to multiple adjacent markers, as previously described [3].

Hotelling's 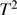-test [36], the multivariate extension of the *t*-test, was used to detect linkage between each marker and the level of gene expression in the four tissues simultaneously. In each independent two-sample test, we also checked the homogeneity of the covariance matrices between the two groups applying the Box's 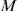 statistics [36] with significant level equal to 0.05. For all non-Bayesian methods, to account for multiple testing of the number of expression traits, we calculated the FDR using the *q*-value approach [37].

### Sparse Bayesian Regression models

Here we are using a Bayesian variable selection (BVS) approach. BVS methods for mapping multiple quantitative loci have been implemented for single trait [25],[26] or extended to consider multiple traits [27]. Besides different choices of prior distribution for the regression coefficients, these methods differ mostly in their implementation of MCMC algorithms, in particular with respect to the update moves that are used and to whether regression coefficients are integrated out or sampled. Gibbs sampling combined with spike and slab priors for the regression coefficients [25],[26] or local adaptation [38] are relatively straightforward to implement but the chains will be highly auto-correlated by construction as when the covariates are non-orthogonal with a strong linear dependence between the regression coefficients. As a result in both cases there could be the tendency to mix slowly. In this vast multi-modal model space analytic integration of the parameters can speed up the convergence of the MCMC: fast mixing is possible because the variable selection indicator does not depend on the value of the effect coefficient [39]. Furthermore, performing a full scan Gibbs sampling of all the covariates at each sweep of the algorithm becomes quickly too computationally demanding when the number of markers is larger than a few hundreds. Our implementation of BVS differs from these works in several key aspects: *i*) a model formulation where regression coefficients are integrated out and not updated at each sweep of the algorithm, *ii*) moves on the model space that involve only the selection indicators and *iii*) a novel class of algorithms, so-called Evolutionary MCMC algorithms, designed to search efficiently multi-modal space by using parallel chains at different temperature [40], discussed in the context of variable selection by [41]. These aspects are important for being able to scale up to well mixing implementations involving thousands of markers. The code for running the SBR and SBMR model will be available upon request from the authors.

Besides the difference in computational schemes associated to BVS, an important extension is the simultaneous analysis of multiple traits. Banerjee *et al.* [27] consider a broad class of multiple traits models, that in particular include a model (referred to as TMV in [27]) similar to the one considered in our paper, where the BVS search is focussed on finding a set of markers associated with all the traits (i.e., expression levels measured in four tissues). However, the flexibility in the model specification in Banerjee *et al.* requires to increase considerably the number of predictors (number of traits × genetic markers), making the search in the model space rather difficult. On the other hand, the mixture over markers model (MOM) proposed in Kendziorski *et al.* [42] is aimed at borrowing information across a large set of traits (e.g., transcripts) in order to better estimate the marginal probability that each marker is a genetic control point. The MOM method is designed for a large set of traits, larger than the number of markers, and hence not appropriate to our context where the number of predictors is substantial and larger than the number of traits. The analysis of multiple complex traits has been also considered in the different context of family-based data and variance component models by Liu *et al.* [38].

#### Likelihood and sparsity

Here we report the likelihood specification for the linear regression model when multiple outcomes are taken into account as well as when a single response is considered. In the former case, the 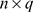 matrix of transcription values 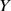 is model as
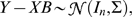
(1)where 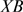 is the linear predictor, with 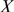 the matrix of markers of dimension 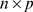 and 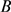 a 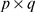 matrix of regression coefficients. 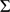 is a 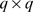 covariance matrix between the 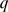 outcomes. 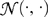 indicates the matrix extension of the centred multivariate normal distribution (matrix-variate normal) [43], where the first argument controls the correlation among the 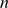 observations and the second one the correlation structure among the 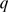 responses. When 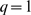, the linear model simplifies to
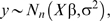
(2)where 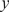 is a 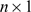 vector of gene expression levels, 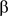 is a 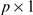 vector of regression coefficients and finally 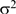 corresponds to the variance of the error term. 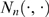 indicates the 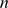-variate normal distribution.

In order to induce sparsity and find a parsimonious model which predicts the multiple outcomes using only a few predictors, we place ourselves in the Bayesian variable selection framework [44] and introduce a latent binary vector 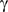 of 0s and 1s of dimension 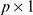 such that 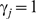 if 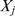, the 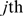 column of 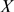 is used as a predictor for 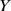 and 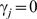 otherwise, 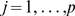. By construction, the 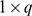 row vector of regression coefficients associated with 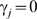 is set equal to 0 with a similar interpretation when 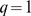. Conditionally on the binary vector 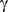, equations (1) and (2) become
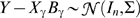
and
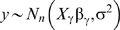
with 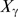 is the original design matrix deprived of the columns that are not used to predict 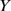 or 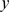.

#### Prior specification

From a Bayesian point of view, uncertainty about the parameters in (1) is introduced by specifying a suitable prior distribution for all the unknowns [45]. The matrix of regression coefficients is distributed as a matrix-variate normal, 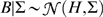, centered in a 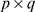 matrix of 0s, where 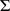 is the covariance matrix of the 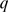 outcomes and 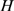 is an appropriate variance-covariance matrix that regulates the dependencies among the 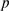 predictors (genetic markers). 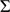 follows an Inverse Wishart distribution, 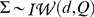, where 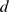 are the degrees of freedom and 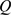 is proportional to the expected value of 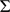, 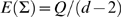. Further simplifications arise fixing 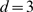, such that the first moment of the Inverse Wishart distribution exists, and imposing 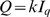, i.e. *a priori* all the 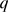 outcomes have the same expected error variance [45]. For the SBR model, priors on the regression coefficients and the error variance greatly simplify [44] with 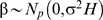, where 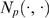 is the 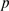-variate normal distribution and 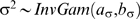.

The specification of the hypermatrix 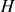 requires particular attention: since it controls the correlation structure of the regression coefficients among the 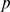 predictors, we decided to model it as 
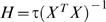
, which together with the prior specification for the matrix of regression coefficients 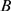 gives rise to the “*g*-prior” set-up [45],[46], i.e., a *priori* the dependency among the 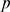 rows of 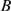 replicates the precision (inverse covariance) structure of the data, thus allowing for marker dependence structure in a natural way. Conditionally on the binary vector 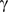, the matrix of regression coefficients 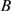 is distributed as 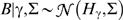, where 
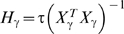
. In the single phenotype case once conditioned on 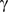 the regression coefficients become 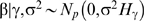.

The coefficient 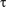 can be interpreted as the relative quantity of information provided by the prior relatively to the sample [47] and its value can influence the results of the variable selection procedure. To avoid arbitrary tuning, we do not fix it, but let it adapt to the data by specifying a prior for 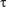 [48], derived from the Jeffreys' prior, a commonly adopted Bayesian specification,
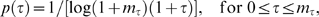
(3)with the support for 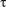 truncated to 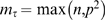, where 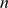 and 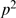 are the number of observations and the squared of the number of predictors. Note that 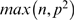 has been proposed as a Benchmark prior by Fernández *et al.* [49]. For large values of 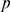, (3) is relatively uninformative with the mode in 0 and finite mean in 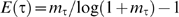. From this point of view, *a priori* we are slightly favoring the null model, i.e. the model that does not include any predictor (genetic marker), a sensible conservative choice. In general it has been found that data adaptivity of the degree of shrinkage conforms better to different variable selection scenarios than assuming standard fixed values [48].

The exchangeable prior on each predictor, 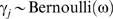, induces a prior over the model size, 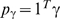, proportional to a Binomial prior, 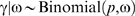. Once the hyperparameter 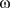 has been integrated out, 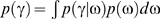, the latent binary vector 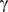 is distributed as a Beta-Binomial prior whose hyperparameters 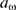 and 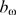 can be worked out once 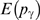 and 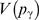, the expected number and the variance of the number of genetic control points for each probe set, are specified [50].

Bearing in mind the likelihood and the prior specification of the parameters, the joint distribution of all variables can be written as
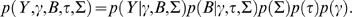
For computational efficiency, the parameters B and Σ can be integrated out leading to
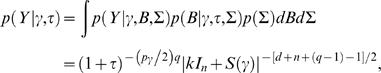
(4)where 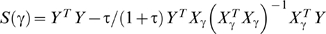. Similar expression can be derived [44] in the case of single response linear regression model, integrating out 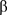 and 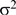 from (2).

#### Evolutionary Stochastic Search

Here we highlight the main features of the algorithm, namely Evolutionary Stochastic Search, ESS hereafter, while interested readers are referred to Bottolo, L. and Richardson S. (2010) Evolutionary Stochastic Search for Bayesian model exploration (http://arxiv.org/abs/1002.2706). Sampling from the target distribution 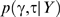 is possible using the full conditionals
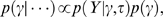
(5)

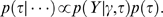
(6)ESS combines two ideas in order to sample from (5) and (6). *i*) Given 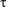, Evolutionary Monte Carlo is used to sample posterior values of 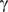: combining a Parallel Tempering [51] sampling scheme with an efficient exchange of information between chains that are run in parallel, each of which with different temperatures (which flatten down the posterior probability of the heated chains), it prevents that the algorithm is trapped in local modes, one of the key problem of stochastic search in high dimensional space. Automatically balancing the computational time spent between local moves, that update locally the chains, and bold moves, that allows the algorithm to jump from a local mode to another, is one of the main features of ESS. An automatic tuning of the temperature ladder during the burn-in, targeting an optimum frequency of exchange of information between chains, contributes to reach marginal convergence; *ii*) Given the population of chains 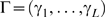, where 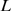 is the number of chains simulated in parallel, the full conditional (6) becomes
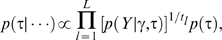
(7)where 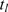, 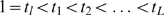, is the temperature attached to the 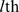 chain. Given the bounded support of (3), 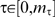 with 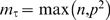, we decided to discretize the support of the prior for computational reasons [47]. This allows the construction of an easy to implement Gibbs sampler. For an alternative sampling scheme for 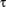, see (http://arxiv.org/abs/1002.2706) for technical details. We chose 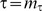 as initial value at the start of the algorithm and we initialised the binary latent vector with 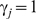 if the variable 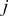 was selected in a simple stepwise regression in each of the tissues.

#### Hyperparameters setting

One of the key features of ESS algorithm applied to SBR or SBMR models is the automatic set-up and tuning of most of the hyperparameters during the burn-in (in particular for the temperature ladder in the population-based MCMC). The only discretionary setting necessary for both SBR or SBMR is the specification of 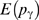 and 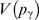, the *a priori* expected value and variance of the model size 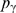, 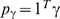, here the number of genetic control points, for a typical probe set in a single tissue or in multiple tissues analysis. We fixed 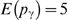 and 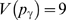, i.e. *a priori* the number of control points ranges roughly between 0 and 12, while values larger that 12 are increasingly penalized. Sensitivity analysis shows that the results are not driven by this particular choice (see Text S1 1.1). The hyperparameter choice for the Inverse Wishart prior distribution for 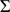 and 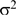 is discussed in Text S1 1.1.

#### Posterior analysis

Details of the running of ESS (number of sweeps and burn-in) are given in Text S1 1.2. Once ESS is run both for SBR and SBMR, we have the sequence 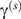, 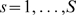, of visited models, or any subset of them, together with their posterior probabilities from which three main quantities of interest. The *marginal posterior probability of inclusion* for each marker 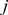 across the models visited:
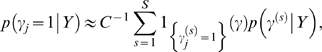
(8)where 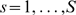 indicates the sequence of sweeps after the burn-in, 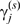 is the binary indicator of *j*th marker at the *s*th sweep, and 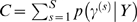. (8) is the weighted frequency (with respect to 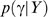) of inclusion for marker *j*, i.e., 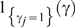. The *model size posterior probability*

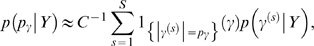
(9)where 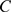 is defined as before. The *best model visited*

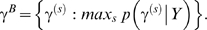
(10)Equation (8) provides a model-averaged measure of importance of each variable (genetic marker) with respect to the models visited, while (9) evaluates the posterior distribution of the number of control points. Rather than providing a single value, (9) quantifies the uncertainty associated with the number of predictors resulting from finding adequate competing explanations involving different set of markers. Multimodality of the search space is a common situation when the number of predictors far outnumbers the number of samples. A simple but effective synthesis of the posterior model size distribution is the mode of 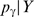. Finally, (10) highlights the best supported multivariate model in terms of its posterior probability.

#### Assessing model importance

Associated to each unique model visited, we define the posterior model probability as the renormalized version of the posterior probability 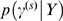, 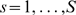, once the parameter 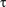 has been integrated out (see Text S1 1.2). Together with this measure of importance of each (unique) visited model among the whole set of visited models, the Bayes Factor [32] indicates how much the data support one particular model *versus* an alternative one. We define 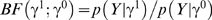, where 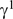 and 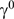 are two models in competition and 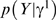 is the marginal probability of model 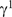 once 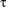 has been integrated out and 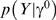 is defined similarly. Since we choose 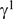 as the best model visited, indicated as 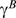, and 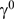 as the null model, indicated as 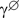, the Bayes Factor compares the strength given by the data of the best model visited with respect to the null model (i.e., no genetic control). Interpreting this ratio in order to select models that are worth reporting is feasible by means of the Jeffreys' scale of evidence [32]. Inspired by Servin and Stephens [52], we decided to calibrate the Jeffreys' scale (for each tissue and for the four tissues together):

1. simulating for each probe set the null model through a reshuffle of the order of the observations;
2. running ESS for SBR and SBMR for the reshuffled transcripts;
3. calculating the Bayes Factor of the best model visited with respect to the null model;
4. selecting the level of the Jeffreys' scale above which the best model visited is considered decisively different from the null model, for a fixed level of the FDR.

In an ideal situation, after the reshuffle, which weakens the genotype–phenotype association, the best model visited and the null model should coincide, 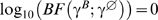. Of course, by chance it will also happen that 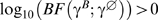, giving rise to a false positive model: a transcript under genetic control is defined to be falsely discovered if all of the predictors in the model 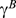 are false positives. For a given threshold of the Jeffreys' scale, which linearly increases according to the best visited model's dimension, we counted the number of transcripts with 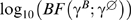 above the threshold in the original data set. These represent the (best visited) models that would be declared different from the null (i.e., positive) at the fixed threshold. Next, for the transcripts that are called positive in the original dataset, we counted the number of the declared false positives after reshuffling. We then adjust the cut-off of the Jeffreys' scale, in symbol 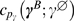, such that the ratio of these two quantities is not greater than 5%. In this way, the Jeffreys' scale is calibrated with respect to the desired FDR level. A similar procedure applies when more reshuffles are performed. Mixure-based FDR calculation is also possible (in the same spirit of Storey *et al.* [28]), although preliminary analysis showed no significant differences with the results obtained using the proposed fully non-parametric procedure.

For the probe sets whose Jeffreys' scale is above the 5% FDR cut-off, as described before for the non-Bayesian mapping, for the best model visited we investigated the position of the putative eQTLs and collapse markers that we found within a 5 cM window, giving rise to a more easily interpretable list of genetic control points. We refer to this refined list of markers as the filtered best model. Although this has been done in a post-processing exercise for ease of interpretation and comparison with other non-Bayesian mapping approaches, ESS takes full advantage, during the model search, of sets of non-redundant closely linked markers in order to better explain the responses' variability (see Text S1 1.4 for illustration).

#### eQTL effect size in the filtered best model

While the posterior density of the regression coefficients can be simulated for each predictor 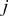 (see Text S1 1.3), here we focus only on the effects sizes of the markers in filtered best model (for a single tissue and for the four tissues together). We want to highlight a robust subset of markers that repeatedly contribute to the set of well supported models whose Jeffreys' scale is above the 5% FDR cut-off. The procedure can be summarized as follows:

1. for a marker 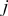 in the filtered best model, we record the fraction of times 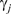 is different from zero over the set of visited models when 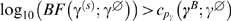, 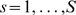;
2. we define a marker in the filtered best model as having a noticeable effect if this fraction is larger than 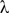 = 0.5 (this fraction 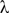 can be increased if a more parsimonious list is required);
3. we simulate the regression coefficients (effect sizes) conditionally on 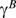 and show only the effect of the markers that fulfilled the above criterion. In the multiple tissue analysis, this will give additional information on the role played by each eQTL within the individual tissue in explaining the pleiotropic effect (see Text S1 1.3 for illustration).

## Supporting Information

Text S1 Supplementary Information.
(1.58 MB PDF)

Figure S1 Correlation structure for the 2,000 transcripts that have the largest variation across tissues. Only 18 probe set pairs, whose Pearson's correlation is above 0.5, are common in the four tissues, while 102,932, 134,690, 82, 508 and 161,341 are the probe set pairs with Pearson's correlation above 0.5 in adrenal, fat, heart and kidney, respectively. This shows that the increment of the pairwise Pearson's positive correlation does not involve the same set of transcripts in the four tissues.
(0.82 MB TIF)

Figure S2 Overview of the Sparse Bayesian Regression (SBR) and Sparse Bayesian Multiple Regression (SBMR) approaches. In the SBR, mRNA levels (y_gh_, with g for the g_th_ probe set and h for the hth tissue, respectively) are modelled at the level of each tissue, y_gh_∼N_n_(Xβ,σ^2^), and the resulting eQTL lists are then compared to find common eQTLs across tissues. In the SBMR approach, mRNA levels of the same transcript measured in four tissues (Y_g_ = [y_g1_, y_g2_, y_g3_, y_g4_]) are modelled jointly, Y_g_−XB∼N (I_n_,Σ), and mapped to the genome to identify pleiotropic genetic control points of gene expression in all tissues. In the multiple tissues analysis the search for a set of markers that jointly predict the level of gene expression is complicated due to the fact that marginally each tissue can be potentially associated to a different group of covariates (mainly *trans*-effects) and share some others (mainly *cis*-effects). The SBMR approach is well powered to identify common genetic regulators even when they have moderate marginal effects.
(3.00 MB TIF)

Figure S3 Distribution of log_10_ Bayes Factor for the best model visited for each transcript (y-axes) versus the number of distinct control points (x-axes) identified in each model after merging closely linked markers (see Materials and Methods). All 531 SBMR models were significant at <5% FDR threshold level, where this threshold was calculated taking into account the size of the best visited model (see Materials and Methods). On average, stronger evidence for common genetic control in all tissues is observed for high-dimensional models.
(3.00 MB TIF)

Figure S4 Genome-wide eQTL linkage results for *Cd36* (A–E) and *Ascl3* (F–L) genes in all tissues simultaneously using Hotelling's T^2^ test (top panels: A, F) and within individual tissues (panels B–E and G–L). For *Cd36* gene the Hotelling's T^2^ test found common genetic regulation in all tissues at the *Cd36* marker; this common eQTL is also detectable by intersecting the results from the single tissues analysis. For *Ascl3* gene, the Hotelling's T^2^ test found the *cis*-eQTL on chromosome 1 (markers *D1Rat55*) but failed to detect the *trans*-eQTL on chromosome 7 (marker *D7Mit8*) at the 5% FDR level. The eQTL results from the individual tissue analysis did not find common *cis*- or *trans*-eQTLs, respectively.
(3.25 MB TIF)

Figure S5 Marginal posterior probability of inclusion obtained from the SBMR and from the SBR analysis within individual tissues. We report the marginal posterior probability for all models visited (top panels) and for the filtered models (bottom panels) whose log_10_ Bayes Factor is above the selected cut-off (see Materials and Methods). (A–E) For *Cd36* gene, the *cis*-regulatory control is consistently found using single tissue modelling (SBR) and the marginal posterior probability of inclusion corresponds to the filtered best model. (F–L) For *Ascl3* gene, neither the *cis*-eQTL or the *trans*-eQTL was systematically detected by the SBR in all tissues, while the SBMR model identified both loci. In adrenal tissue, the filtered models did not show any genetic control points at FDR <5% (G, bottom panel).
(3.25 MB TIF)

Figure S6 Validation of microarray gene expression linkages by RT-PCR. We replicated *cis*-eQTL linkages for: (A–D) *Endog* (Jeffreys' scale = 14.2) and (E–H) *Card9* (Jeffreys' scale = 9.9), showing strong *cis* regulation in the heart tissue at markers *D3Cebr204s4* and *D3Cebr83s1*, respectively. For each *cis*-eQTL we report the linkage results by *t*-test (panel A, E), by the SBR model (panel B, F), and expression values by BN and SHR genotype at the peak marker by microarray (panel C, G) and by RT-PCR (panel D, H). Expression data are reported as mean ± sem. Consistently with the microarray results, the RT-PCR data show significant evidence for cis-linkage for both genes. (*P<0.001)
(3.00 MB TIF)

Figure S7 Validation of small-effect *trans*-eQTLs by RT-PCR. We replicated *trans*-eQTL linkages for: (A–D) *Stat4* (Jeffreys' scale = 2.8) and (E–H) *Irf7* (Jeffreys' scale = 2.7), both showing *trans*-acting regulation at marker *D15Rat107* in the heart tissue with FDR <5%. For each *trans*-eQTL we report the linkage results by QTL Reaper (panel A, E), by the SBR model (panel B, F), and expression values by BN and SHR genotype at the peak marker (*D15Rat107*) by microarray (panel C, G) and by RT-PCR (panel D, H). QTL Reaper identified the *trans*-eQTL for *Stat4* with genome-wide *P*-value (*P*_GW_) = 0.008 (FDR = 5%) and for *Irf7* with *P*_GW_ = 0.04 (FDR = 28%). For comparison, the SSM found *trans*-linkages for *Stat4* and *Irf7* at FDR = 5% and FDR = 17%, respectively. Expression data are reported as mean ± sem. Consistently with the microarray results, the RT-PCR data show significant evidence for *trans*-linkage for both genes. (*P<0.05, **P<0.01)
(3.00 MB TIF)

Table S1 Summary statistics of heritability of mRNA levels for the 2,000 transcripts considered in this study.
(0.03 MB DOC)

Table S2 Number of probe sets found to be under genetic control in the SBR and SBMR analyses (FDR 1% and 0.5%).
(0.05 MB DOC)

Table S3 Comparison between SBR, SSM and QTL Reaper results.
(0.06 MB DOC)

Table S4 Polygenic models that have been detected in at least one tissue by the SBR model (FDR <5%).
(0.10 MB PDF)

Table S5 eQTLs that were detected in common to all tissues by the SBR model (FDR <5%).
(0.09 MB PDF)

Table S6 Cis-regulated transcripts found by both SBMR and the Hotelling's T2-test at 5% FDR.
(0.09 MB PDF)

## References

[1] Brem RB, Yvert G, Clinton R, Kruglyak L (2002). Genetic dissection of transcriptional regulation in budding yeast.. Science.

[2] Morley M, Molony CM, Weber TM, Devlin JL, Ewens KG (2004). Genetic analysis of genome-wide variation in human gene expression.. Nature.

[3] Hubner N, Wallace CA, Zimdahl H, Petretto E, Schulz H (2005). Integrated transcriptional profiling and linkage analysis for identification of genes underlying disease.. Nat Genet.

[4] Chesler EJ, Lu L, Shou S, Qu Y, Gu J (2005). Complex trait analysis of gene expression uncovers polygenic and pleiotropic networks that modulate nervous system function.. Nat Genet.

[5] Goring HH, Curran JE, Johnson MP, Dyer TD, Charlesworth J (2007). Discovery of expression QTLs using large-scale transcriptional profiling in human lymphocytes.. Nat Genet.

[6] Dixon AL, Liang L, Moffatt MF, Chen W, Heath S (2007). A genome-wide association study of global gene expression.. Nat Genet.

[7] Schadt EE, Molony C, Chudin E, Hao K, Yang X (2008). Mapping the genetic architecture of gene expression in human liver.. PLoS Biol.

[8] Emilsson V, Thorleifsson G, Zhang B, Leonardson AS, Zink F (2008). Genetics of gene expression and its effect on disease.. Nature.

[9] Bystrykh L, Weersing E, Dontje B, Sutton S, Pletcher MT (2005). Uncovering regulatory pathways that affect hematopoietic stem cell function using ‘genetical genomics’.. Nat Genet.

[10] Gilad Y, Rifkin SA, Pritchard JK (2008). Revealing the architecture of gene regulation: the promise of eQTL studies.. Trends Genet.

[11] Petretto E, Mangion J, Dickens NJ, Cook SA, Kumaran MK (2006). Heritability and Tissue Specificity of Expression Quantitative Trait Loci.. PLoS Genet.

[12] Wang SS, Schadt EE, Wang H, Wang X, Ingram-Drake L (2007). Identification of pathways for atherosclerosis in mice: integration of quantitative trait locus analysis and global gene expression data.. Circ Res.

[13] Petretto E, Sarwar R, Grieve I, Lu H, Kumaran MK (2008). Integrated genomic approaches implicate osteoglycin (Ogn) in the regulation of left ventricular mass.. Nat Genet.

[14] West MA, Kim K, Kliebenstein DJ, van Leeuwen H, Michelmore RW (2007). Global eQTL mapping reveals the complex genetic architecture of transcript-level variation in Arabidopsis.. Genetics.

[15] Potokina E, Druka A, Luo Z, Moscou M, Wise R (2008). Tissue-dependent limited pleiotropy affects gene expression in barley.. Plant J.

[16] Peirce JL, Li H, Wang J, Manly KF, Hitzemann RJ (2006). How replicable are mRNA expression QTL?. Mamm Genome.

[17] Breitling R, Li Y, Tesson BM, Fu J, Wu C (2008). Genetical genomics: spotlight on QTL hotspots.. PLoS Genet.

[18] Yang X, Schadt EE, Wang S, Wang H, Arnold AP (2006). Tissue-specific expression and regulation of sexually dimorphic genes in mice.. Genome Res.

[19] Hovatta I, Zapala MA, Broide RS, Schadt EE, Libiger O (2007). DNA variation and brain region-specific expression profiles exhibit different relationships between inbred mouse strains: implications for eQTL mapping studies.. Genome Biol.

[20] Bence KK, Delibegovic M, Xue B, Gorgun CZ, Hotamisligil GS (2006). Neuronal PTP1B regulates body weight, adiposity and leptin action.. Nat Med.

[21] Dobrin R, Zhu J, Molony C, Argman C, Parrish ML (2009). Multi-tissue coexpression networks reveal unexpected subnetworks associated with disease.. Genome Biol.

[22] Tibshirani R (1996). Regression shrinkage and selection via the lasso.. J Royal Statist Soc B.

[23] Hoggart CJ, Clark TG, De Iorio M, Whittaker JC, Balding DJ (2008). Genome-wide significance for dense SNP and resequencing data.. Genet Epidemiol.

[24] Kim S, Xing EP (2009). Statistical estimation of correlated genome associations to a quantitative trait network.. PLoS Genet.

[25] Zhang M, Montooth KL, Wells MT, Clark AG, Zhang D (2005). Mapping multiple Quantitative Trait Loci by Bayesian classification.. Genetics.

[26] Zhang M, Zhang D, Wells MT (2008). Variable selection for large p small n regression models with incomplete data: mapping QTL with epistases.. BMC Bioinformatics.

[27] Banerjee S, Yandell BS, Yi N (2008). Bayesian quantitative trait loci mapping for multiple traits.. Genetics.

[28] Storey JD, Akey JM, Kruglyak L (2005). Multiple locus linkage analysis of genomewide expression in yeast.. PLoS Biol.

[29] Williams RB, Cotsapas CJ, Cowley MJ, Chan E, Nott DJ (2006). Normalization procedures and detection of linkage signal in genetical-genomics experiments.. Nat Genet.

[30] Monti J, Fischer J, Paskas S, Heinig M, Schulz H (2008). Soluble epoxide hydrolase is a susceptibility factor for heart failure in a rat model of human disease.. Nat Genet.

[31] Aitman TJ, Glazier AM, Wallace CA, Cooper LD, Norsworthy PJ (1999). Identification of Cd36 (Fat) as an insulin-resistance gene causing defective fatty acid and glucose metabolism in hypertensive rats.. Nat Genet.

[32] Kass RE, Raftery AE (1995). Bayes Factors.. Journal of the American Statistical Association.

[33] Wallis RH, Collins SC, Kaisaki PJ, Argoud K, Wilder SP (2008). Pathophysiological, genetic and gene expression features of a novel rodent model of the cardio-metabolic syndrome.. PLoS ONE.

[34] Siegmund D, Yakir B (2007).

[35] Irizarry RA, Bolstad BM, Collin F, Cope LM, Hobbs B (2003). Summaries of Affymetrix GeneChip probe level data.. Nucleic Acids Res.

[36] Johnson RA, Wichern DW (1992). Applied Multivariate Statistical Analysis: 3rd. ed.

[37] Storey JD (2002). A direct approach to false discovery rates.. J R Statist Soc B.

[38] Liu J, Liu Y, Liu X, Deng HW (2007). Bayesian mapping of quantitative trait loci for multiple complex traits with the use of variance components.. Am J Hum Genet.

[39] O'Hara RB, Sillanpää MJ (2009). Review of Bayesian variable selection methods: what, how and which.. Bayesian Analysis.

[40] Liang F, Wong WH (2000). Evolutionary Monte Carlo: application to Cp model sampling and change point problem.. Stat Sinica.

[41] Jasra A, Stephens DA, Holmes CC (2007). Population-Based Reversible Jump Markov Chain Monte Carlo.. Biometrika.

[42] Kendziorski CM, Chen M, Yuan M, Lan H, Attie AD (2006). Statistical methods for expression quantitative trait loci (eQTL) mapping.. Biometrics.

[43] Dawid AP (1981). Some matrix-variate distribution theory: notational considerations and a Bayesian application.. Biometrika.

[44] Chipman H, George EI, McCulloch RE, Lahiri P (2001). The practical implementation of Bayesian model selection (with discussion)..

[45] Brown PJ, Vannucci M, Fearn T (1998). Multivariate Bayesian variable selection and prediction.. J R Statist Soc B.

[46] Zellner A, Zellner PKGaA (1986). On assessing prior distributions and Bayesian regression analysis with g-prior distributions..

[47] Robert C, Marin J-M (2008).

[48] Liang F, Paulo R, Molina G, Clyde MA, Berger JO (2008). Mixtures of g-priors for Bayesian variable selection.. J Am Statist Assoc.

[49] Fernández C, Ley E, Steel MFJ (2001). Benchmark priors for Bayesian model averaging.. J Econometrics.

[50] Kohn R, Smith M, Chan D (2001). Nonparametric regression using linear combinations of basis functions.. Statist Comp.

[51] Iba Y (2001). Extended Ensemble Monte Carlo.. Int J Mod Phys, C.

[52] Servin B, Stephens M (2007). Imputation-based analysis of association studies: candidate regions and quantitative traits.. PLoS Genet.

