# Supplementary material for: New Insights into the Genetic Control of Gene Expression using a Bayesian Multi-tissue Approach
Source: PLoS Comput Biol. 2010 Apr 8;6(4):e1000737. doi: 10.1371/journal.pcbi.1000737 (PMC2851562; doi:10.1371/journal.pcbi.1000737)
Supplement: Text S1 — Supplementary Information. (1.58 MB PDF) [file pcbi.1000737.s001.pdf]

SUPPLEMENTARY INFORMATION

New insights into the genetic control of gene expression using a Bayesian multi-tissue approach

Enrico Petretto[1,2,*], Leonardo Bottolo[2,1,*], Sarah R. Langley[1], Matthias Heinig[3], Chris McDermott-Roe[1], Rizwan Sarwar[1], Michal Pravenec[4,5], Norbert Hübner[3], Timothy J. Aitman[1,6], Stuart A. Cook[1,7] and Sylvia Richardson[2]

1 Medical Research Council Clinical Sciences Centre, Faculty of Medicine, Imperial College London, London, UK.

2 Department of Epidemiology and Biostatistics, Faculty of Medicine, Imperial College, London, UK.

3 Max-Delbrück Center for Molecular Medicine, Berlin, Germany.

4 Institute of Physiology, Czech Academy of Sciences and Centre for Applied Genomics, Prague, Czech Republic.

5 Charles University in Prague, Institute of Biology and Medical Genetics of the First Faculty of Medicine and General Teaching Hospital, Prague, Czech Republic.

6 Section of Molecular Genetics and Rheumatology, Division and Faculty of Medicine, Imperial College, London, UK.

7 National Heart and Lung Institute, Imperial College, London, UK.

* These authors contributed equally to this work.

Correspondence should be addressed to:

Sylvia Richardson

Department of Epidemiology and Biostatistics

Faculty of Medicine, Imperial College

Norfolk Place

London, W2 1PG, UK

Email: sylvia.richardson@imperial.ac.uk

1 Sparse Bayesian Regression models
1.1 Hyperparameters setting
1.2 Number of sweeps and convergence
1.3 Effects size
1.4 Illustrative examples of filtered best model and noticeable effects
2 Comparison with non-Bayesian mapping approaches
2.1 Adapted two-stage Sequential Search Method (SSM)
2.2 Comparison between SBR and SSM
2.3 Comparison between SBR and QTL Reaper
2.4 Comparison between SBR, SSM and QTL Reaper
2.5 Comparison between SBMR and Hotelling's T^2-test
2.6 Power comparison with Hotelling's T^2-test and GFlasso
3 Validation of microarray gene expression data

1. Sparse Bayesian Regression models

1.1 Hyperparameters setting

In this paper we use $E(p_\gamma)=5$ and $V(p_\gamma)=9$, allowing the number of predictors to range between 0 and 12, a sensible choice in keeping with biological knowledge on the number genetic controls. In order to judge the sensitivity of the results to the prior on the model size, we also ran our SBR and SBMR models with an alternative setting: $E(p_\gamma)=2$ and $V(p_\gamma)=1$, that is *a priori* the number of predictors ranges roughly between 0 and 5. Overall, for the single tissues analyses, the results did not change demonstrating that the information contained in the data dominates the prior distribution on p_γ. Note that the adaptive level of shrinkage provided by τ stabilises the results with respect to different choices of the prior model size.

While for the SBR model the error variance follows an Inverse Gamma distribution, $\sigma^2 \sim InvGam(a_\sigma, b_\sigma)$ with a_σ and b_σ chosen such that the prior on σ^2 is non-informative ($a_\sigma = 10^{-10}, b_\sigma = 10^{-3}$), setting the parameter k for the expectation of the error variance in the SBMR model, $E(\Sigma) = kI_q$, is more complicated and, in contrast to previous reported analyses [1], we found some sensitivity to the value of k even when the degrees of freedom are very small such as $d = 3$. Here we propose a practical way to fix the hyperparameter k in the spirit of an Empirical Bayes approach [2]: firstly, given a probe set, for every tissue, $h = 1,\ldots,4$, we perform a stepwise regression (SWR), on the whole set of markers which enables us to derive an approximate estimate of the error variance $\hat{\sigma}_h^{2\text{SWR}}$. Combining the results derived from the stepwise regressions in the four tissues, we fix k to be the average of the approximate error variance for each tissue, $k = \sum_{h=1}^{4} \hat{\sigma}_h^{2\text{SWR}} / 4$.

1.2 Number of sweeps and convergence

We chose the number of chains to be run in parallel equal to four, which guarantees a sufficient ability of ESS to explore far apart regions of high posterior density $p(\gamma, \tau | Y)$. In the SBR model the algorithm is run for 25,000 sweeps, which 5,000 as burn-in. In the SBMR model we decided to increase the number of sweeps by roughly a factor of four such that the number of sweeps becomes 110,000 with 10,000 as burn-in. For 770 markers the average computational time for SBR is around 21 minutes ran on a desktop computer with a 2GHz processor and 2Gb memory, while for SBMR the computational time ranges between 80 and 115 minutes depending on the number of control points found during the search.

Performance of the ESS algorithm in the SBR case was extensively tested by simulations, including a test case based on HapMap on a set of 775 non-redundant SNPs

and real data examples in a space up to 10,000 predictors. Typically the stationary distribution is reached by the end of the burn-in period and the simulated effects have high posterior probability of inclusion. For SBMR, the search of a set of markers that jointly predict the level of gene expression in the four tissues is complicated due to the fact that marginally each tissue can be potentially associated to a different groups of covariate (mainly *trans* effects) and share some others (mainly *cis* effects). For that reason, after running the ESS for the selected 2,000 probe sets for the SBMR model with 110,000 sweeps, we recorded, in a post processing analysis, how many times the algorithm visited the top model ranked by the posterior probability

$$p\left(\gamma^{(s)}|Y\right)=\int p\left(\gamma^{(s)}|Y,\tau\right)p(\tau)d\tau$$
$$\approx \sum_{r=1}^{S} p\left(\gamma^{(s)}|Y,\tau^{(r)}\right),$$

where $s,r=1,\ldots,S$ is the sequence of sweeps after burn-in and $p\left(\gamma^{(s)}|Y,\tau^{(r)}\right)\propto p\left(Y|\gamma^{(s)},\tau^{(r)}\right)p\left(\gamma^{(s)}\right)$. If the algorithm reached the top model less than 25 times in each of the two halves of the sweeps, we increased the number of sweeps to ensure a faithful exploration of the posterior probability. According to this criterion, we reran ESS for SBMR in 557 probe sets (~28%) using 510,000 sweeps with 10,000 sweeps as burn-in. Only for seven (< 1%) of the remaining probe sets, the top ranked model was visited less than 25 times in each of the two halves: in these cases, we visually inspected the trace of $p(\gamma|Y)$ and checked that the algorithm reached the top model at nearly evenly spread intervals, reassuring that the search detect a good combination of markers.

1.3 Effects size

Given a value of $\gamma^{(s)}$ and $\tau^{(s)}$, B, the matrix of regression coefficients and Σ, the error variance matrix, can be simulated as follows [3]

$$\Sigma^{(s)} \Big| Y, \gamma^{(s)}, \tau^{(s)} \sim \mathcal{IW}\left(d^*, Q^*\right), \quad \text{(S.1)}$$

$$\left(B^{(s)} + m^*\right) \Big| Y, \gamma^{(s)}, \tau^{(s)}, \Sigma^{(s)} \sim \mathcal{N}\left(H^*_{\gamma^{(s)}}, \Sigma^{(s)}\right), \quad \text{(S.2)}$$

where

- $H^*_{\gamma^{(s)}} = \dfrac{\tau^{(s)}}{1+\tau^{(s)}}\left(X^T_{\gamma^{(s)}} X_{\gamma^{(s)}}\right)^{1}$,
- $d^* = d^* + n$,
- $m^* = H^*_{\gamma^{(s)}} X^T_{\gamma^{(s)}} Y = \dfrac{\tau^{(s)}}{1+\tau^{(s)}} B^{\mathrm{LS}}_{\gamma^{(s)}}$,
- $$\begin{aligned} Q^* &= kI_n + Y^T Y - m^{*T}\left(H^*_{\gamma^{(s)}}\right)^{-1} m^* \\ &= kI_n + Y^T Y - \frac{\tau^{(s)}}{1+\tau^{(s)}} Y^T X_{\gamma^{(s)}}\left(X^T_{\gamma^{(s)}} X_{\gamma^{(s)}}\right)^{-1} X^T_{\gamma^{(s)}} Y, \end{aligned}$$

with $B^{\mathrm{LS}}_{\gamma^{(s)}}$, the $p \times q$ matrix of solutions of the least squares given the set of covariates selected in the sth model. For $\gamma^{(s)} = \gamma^B$, we average the posterior values of $\left(B^B + m^*\right)$ and Σ^B over the set of τ that are associated with the best model visited.

1.4 Illustrative examples of filtered best model and noticeable effects

Here we show that the inclusion of non-redundant closely linked markers helps ESS to identify *trans*-acting eQTLs that would have been missed otherwise. We present here an example for transcript 1371960_at in heart tissue. Figure 1 below shows the posterior probability of inclusion for the visited models with $\log_{10}\left(BF\left(\gamma^{(s)};\gamma^{\varnothing}\right)\right) > c_{p_\gamma}\left(\gamma^B;\gamma^{\varnothing}\right)$,

$s = 1, \ldots, S$. The best model visited has four genetic control points: *Igf2* on chromosome 1 at 151.2 cM; *D1Arb22* on chromosome 1 at 154.2 cM; *Jak2* on chromosome 1 at 193.9 cM, and *D5Rat38* on chromosome 5 at 1003.7 cM, indicated by the arrows. The first three markers are *trans*-acting loci (Figure 1, insert) while the marker on chromosome 5 is a *cis*-acting eQTL for transcript 1371960_at. For comparison reasons with other non-Bayesian methods, we collapsed markers in the best model visited that we found within a 5 cM window, giving rise the filtered best model that includes two distinct *trans*-eQTLs and one *cis*-eQTL. However, inclusion of both non–redundant markers, *Igf2* and *D1Arb22*, is essential to discover (at least) one of the two *trans*-acting eQTLs. To show this, we excluded *Igf2* from the list of markers and ran ESS for the same number of iterations as before. The *cis*-acting eQTL was found in the best model visited, but neither *D1Arb22* nor *Jak2* were detected. This suggests that the ESS exploits the combined effects of tightly linked markers to facilitate identification of secondary effects, i.e., *trans*-eQTLs.

The second illustrative example shows the steps that we took to reduce the dimension of the best model visited for transcript 1374907_at for the four tissues together. ESS indentifies a group of nine distinct genetic control points indicated with arrows in Figure 2A below. As described in Materials and Methods, we perform the following steps to highlight noticeable effects:

1. for each of the nine markers we count the fraction of times γ_j is different from zero in the set of visited model above the suitable FDR cut-off for the Jeffrey's scale;
2. five markers out of nine, having marginal posterior probability of inclusion > 0.5, are declared having noticeable effects (indicated in red in Figure 2A), reducing nearly by half the number of control points: *D1Rat42* on chromosome 1 at 124.2

cM; *D1Rat47* on chromosome 1 at 136.7 cM; *D2mit6* on chromosome 2 at 68.8 cM; *D6Cep8* on chromosome 6 at 12 cM; *Fh* on chromosome 13 at 88.3 cM;

3. conditionally on the best visited model γ^B, we simulate the effects using (S.1) and (S.2), reporting only the effects corresponding to the markers that fulfilled the above condition (Figure 2 panels B-E, for each tissue separately).

The transcript 1374907_at is located on chromosome 1, the *cis*-acting eQTL (*D1Rat47*) has marginal posterior probability equal to one, with a consistent negative effect in all tissues, while the other *trans*-acting eQTL on chromosome 1 (*D1Rat42*) shows a weaker marginal association with the gene expression level. For *D1Rat42,* visual inspection of the simulated regression coefficients indicates that the contribution to the linkage provided by kidney is less pronounced than for the other tissues. A similar situation arises for *D2mit*, that is the stronger *trans*-acting eQTL according to the marginal posterior probability of inclusion: the posterior distribution of the regression coefficient in kidney is concentrated near zero, while is large and negative in adrenal and relative small and positive in fat and heart indicating tissue-specific allelic effects. The choice of a 0.5 cut-off level for the fraction of times γ_j is different from zero is a pragmatic choice although larger values can be specified to select more parsimonious models. Alternatively, to control FDR level, the threshold level can be chosen following Chen *et al.* [4].

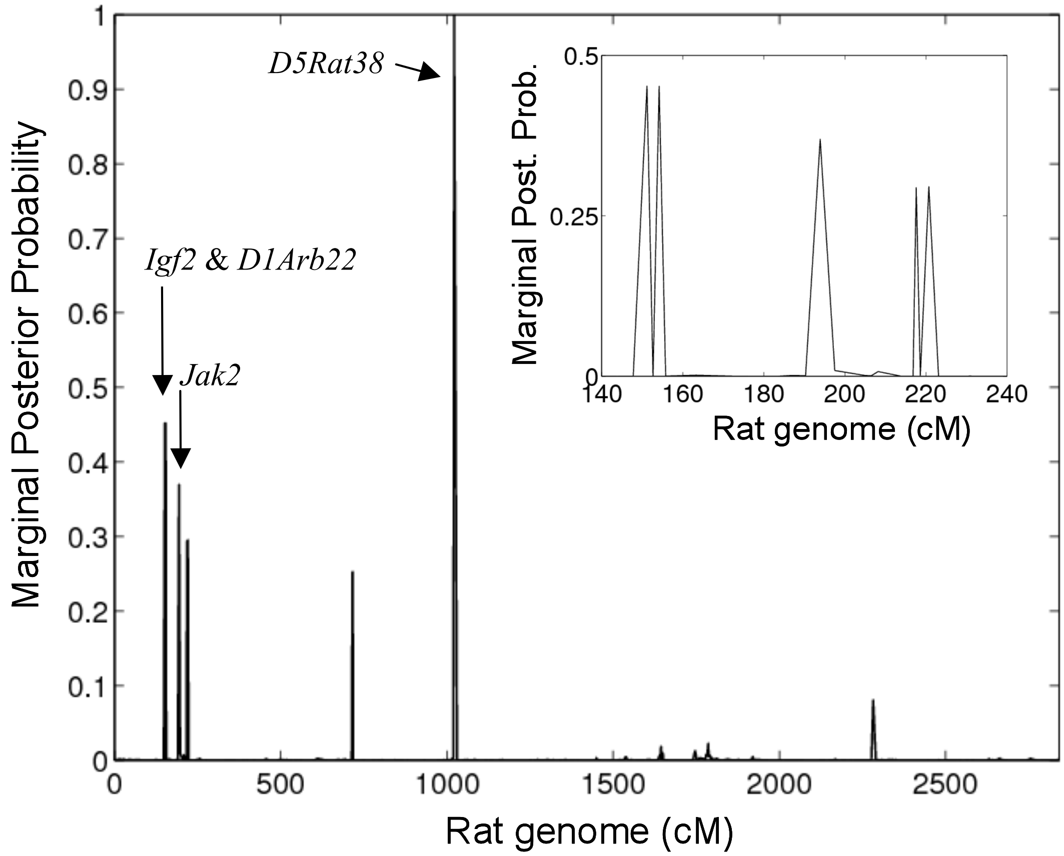

Figure 1. Marginal posterior probability of inclusion of each genetic marker for transcript 1371960_at in the heart tissue. Arrows indicate the best model visited, defined by four distinct genetic control points (*Igf2* on chromosome 1 at 151.2 cM; *D1Arb22* on chromosome 1 at 154.2 cM; *Jak2* on chromosome 1 at 193.9 cM, and *D5Rat38* on chromosome 5 at 1003.7 cM). The filtered best model is obtained by removing redundant markers within a 5 cM window, thus resulting in three distinct eQTLs. Insert, magnification of the region comprising *Igf2, D1Arb22* and *Jak2* genetic markers. The marginal probability of inclusion is calculated conditionally on all visited models whose $\log_{10}$ Bayes Factor is above the calibrated threshold controlling FDR at 5% level.

A

B

C

D

E

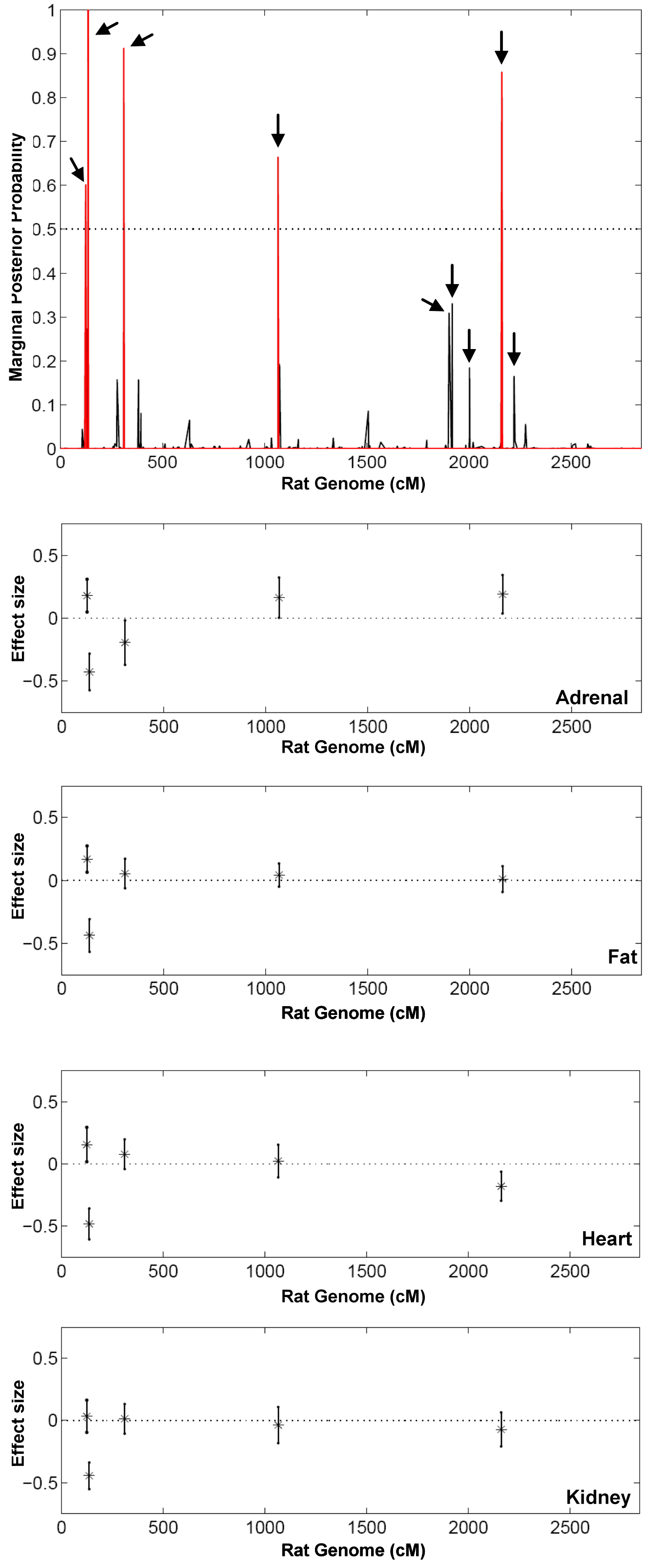

Figure 2. A) Marginal posterior probability of inclusion of each genetic marker for transcript 1374907_at in all tissues identified by the SBMR model. Arrows indicate the nine control points in the best model visited (FDR < 5%). The distinct eQTLs with noticeable effects are highlighted in red. B-E) Posterior density of the regression coefficients (i.e., effect size) for each marker with noticeable effect in each tissue. For each effect, the posterior interquantile range is indicated by the vertical bars, and the posterior median with a star.

2. Comparison with non-Bayesian mapping approaches

2.1 Adapted two-stage Sequential Search Method (SSM)

The two-stage Sequential Search Method, SSM hereafter, proposed by Storey et al. [5] for each gene expression trait, maps the most significant QTL and then sequentially identifies an additional QTL conditional on the first QTL found. With a slight modification of Storey's notation, the joint statistics for the 2-QTLs model is calculated based on

$$\begin{aligned} M_0 &: y_h = m + \varepsilon, \\ M_1 &: y_h = m + \mathrm{QTL}_1 + \varepsilon, \\ M_2 &: y_h = m + \mathrm{QTL}_1 + \mathrm{QTL}_2 + \mathrm{QTL}_1 \times \mathrm{QTL}_2 + \varepsilon, \end{aligned} \tag{S.3}$$

where y_h, $h = 1,\ldots,4$, is the transcript level for the hth tissue, m is the intercept, ε is the normal error term, $\varepsilon \sim N_n\left(0,\sigma^2 I_n\right)$, and finally $\mathrm{QTL}_1 = \beta_j X_j$ and $\mathrm{QTL}_2 = \beta_{j'} X_{j'}$ with $j = 1,\ldots,p$, $j \neq j'$.

In this paper, we have adapted SSM to make it comparable with the SBR and QTL Reaper analyses. The sequential method for locating the two loci remained the same, but the epistatic interaction between the primary and secondary locus, i.e. $\mathrm{QTL}_1 \times \mathrm{QTL}_2$ in (S.3), is not considered. The model given below by M_2^* is then directly comparable to the additive the SBR model which does not consider epistatic interactions between eQTLs although its inclusion would not be difficult, but requires a different specification for the prior probability of the epistatic interaction [6]. The modified SSM that we consider is

$$\begin{aligned} M_0 &: y_h = m + \varepsilon, \\ M_1 &: y_h = m + \mathrm{QTL}_1 + \varepsilon, \\ M_2^* &: y_h = m + \mathrm{QTL}_1 + \mathrm{QTL}_2 + \varepsilon. \end{aligned} \tag{S.4}$$

A Bayesian representation of the posterior probability of linkage for the models in (S.4) was adopted. The R package q-value available at the web site

http://www.genomine.org/qvalue/, was used to calculate the posterior probability of linkage [7] for the adapted SSM.

This above SSM approach was applied to the RI strains population, consisting of 29 distinct observations [8]. Across tissues, 65% to 81% of the transcripts found by SBR to have one significant eQTL were also identified by SSM. However, SBR found 12-15% of the transcripts with at least one significant eQTL are under polygenic control, whereas SSM found no transcripts with more than one locus significantly associated with it. Using SSM, a population size of 29 strains does not have enough power to identify any significant multiple eQTL interactions as the method is limited by the power of the Wilcox test for the secondary locus (data not shown).

2.2 Comparison between SBR and SSM

Figure 3 below shows the number of transcripts which were found by the SBR model (FDR < 5% across all tissues), by the SSM model and those in common between the two methods when one locus found in the filtered best SBR model (see Materials and Methods) is also called significant by SSM (after removing redundant eQTLs, see Materials and Methods) at FDR < 5% across all tissues. Consistently across tissues, SBR found similar number of eQTLs (or more eQTLs that SSM), with the majority of them identified using both approaches. Figure 4 below shows the *cis*- and *trans*-regulated transcripts which were found by both methods for each tissue. The commonly detected eQTLs are primarily regulated in *cis* (66% to 72% across tissues), 14% to 24% of common loci were *trans* regulated and the rest had unreliable positions, suggesting that the both methods have high power to detect *cis*-acting effects within individual tissues.

However, out the transcripts identified by both methods, the SBR model identified several transcripts per tissue that had polygenic control (12% to 15%) while modified Storey's

approach did not find any transcripts with polygenic control. Figure 5 below shows the total number of transcripts found by both methods. Those transcripts that the SBR model found to have polygenic control and SSM found to have monogenic control are given in grey, and those which both found to be monogenic are given in black (Figure 5).

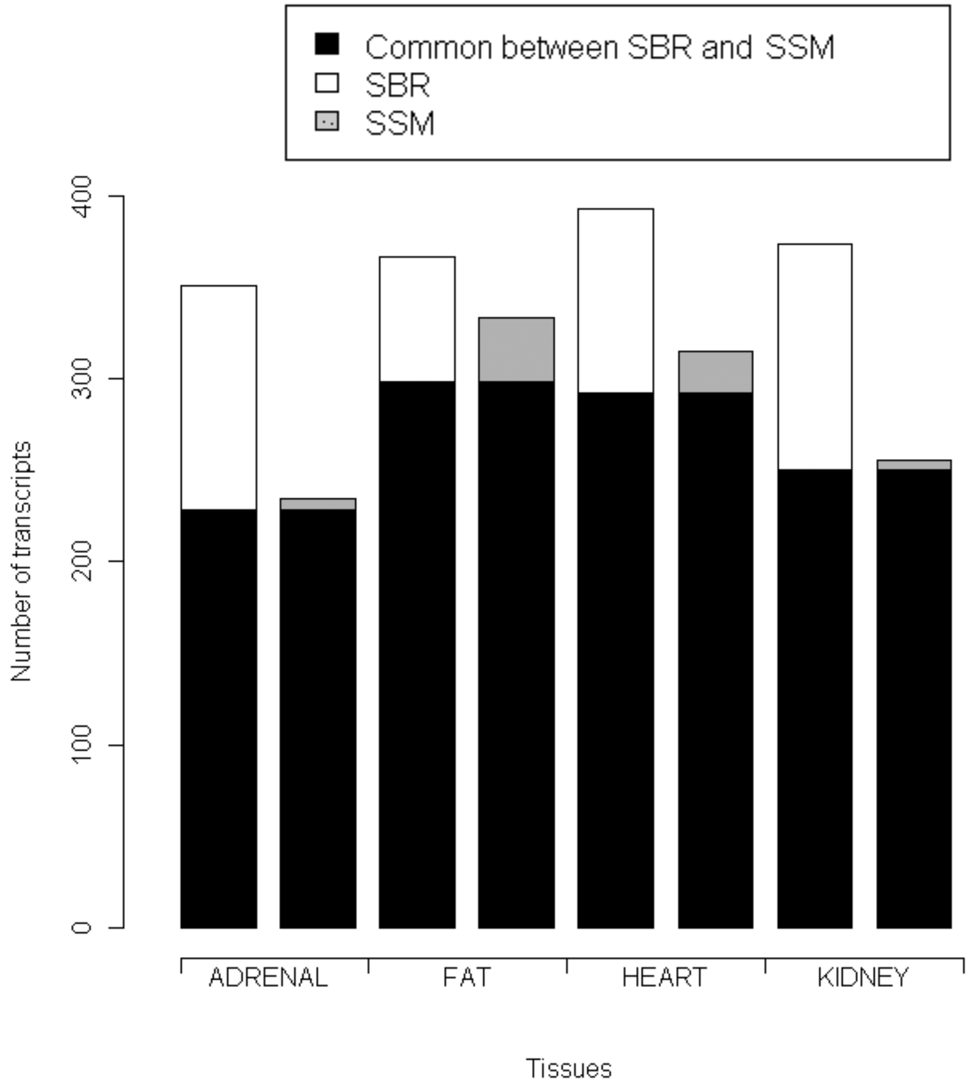

Figure 3. Number of significant transcripts found by SBR and SSM at FDR < 5%. Transcripts were considered to be in common if one locus in the filtered best SBR model matched one locus found by SSM after removing redundant eQTLs which may result from linkage of expression values to multiple adjacent markers.

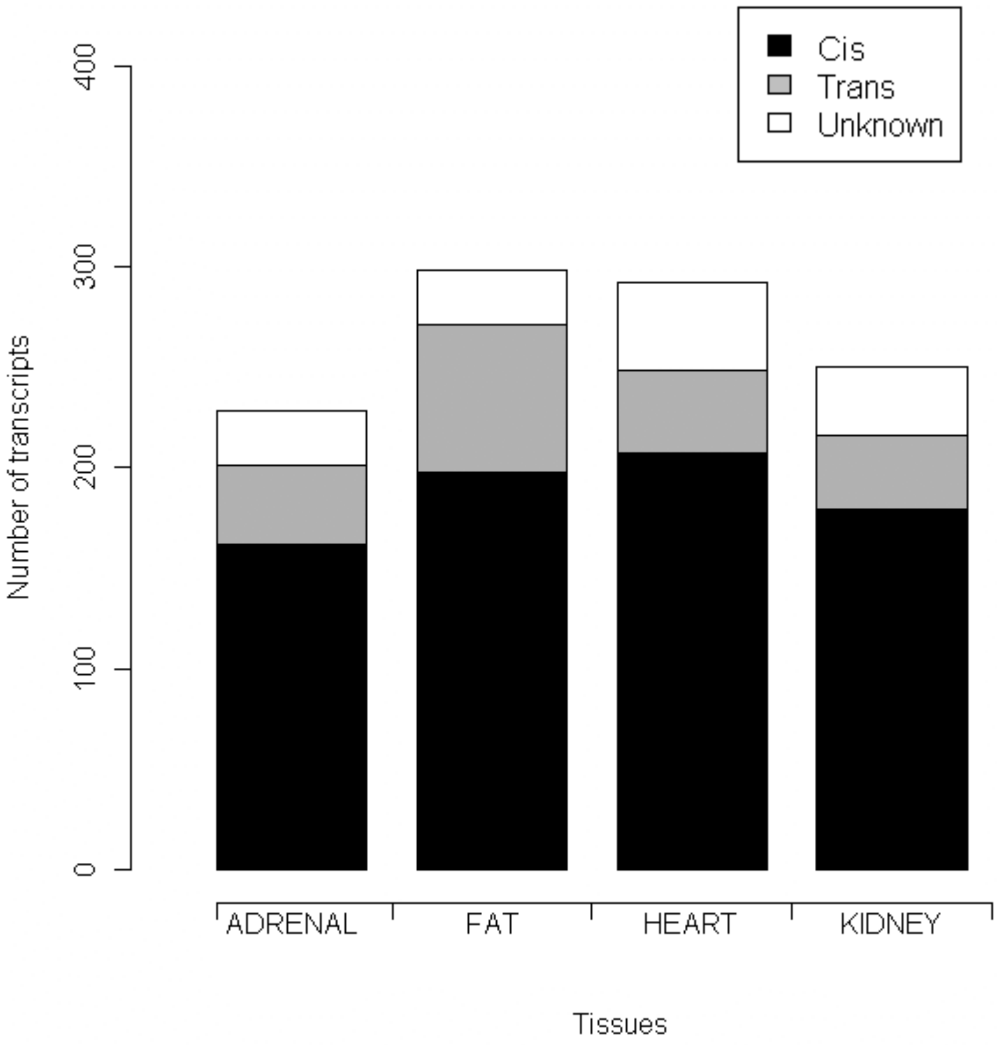

Figure 4. Number of *cis*-acting, *trans*-acting and unknown eQTLs found in common between SBR and SSM (in significant transcripts). Transcripts were considered to be in common if one locus in the filtered best SBR model matched one locus found by SSM after removing redundant eQTLs which may result from linkage of expression values to multiple adjacent markers. An eQTL was called *cis*-acting if it fell into a 10 Mb region around the localization of the transcript (see Materials and Methods).

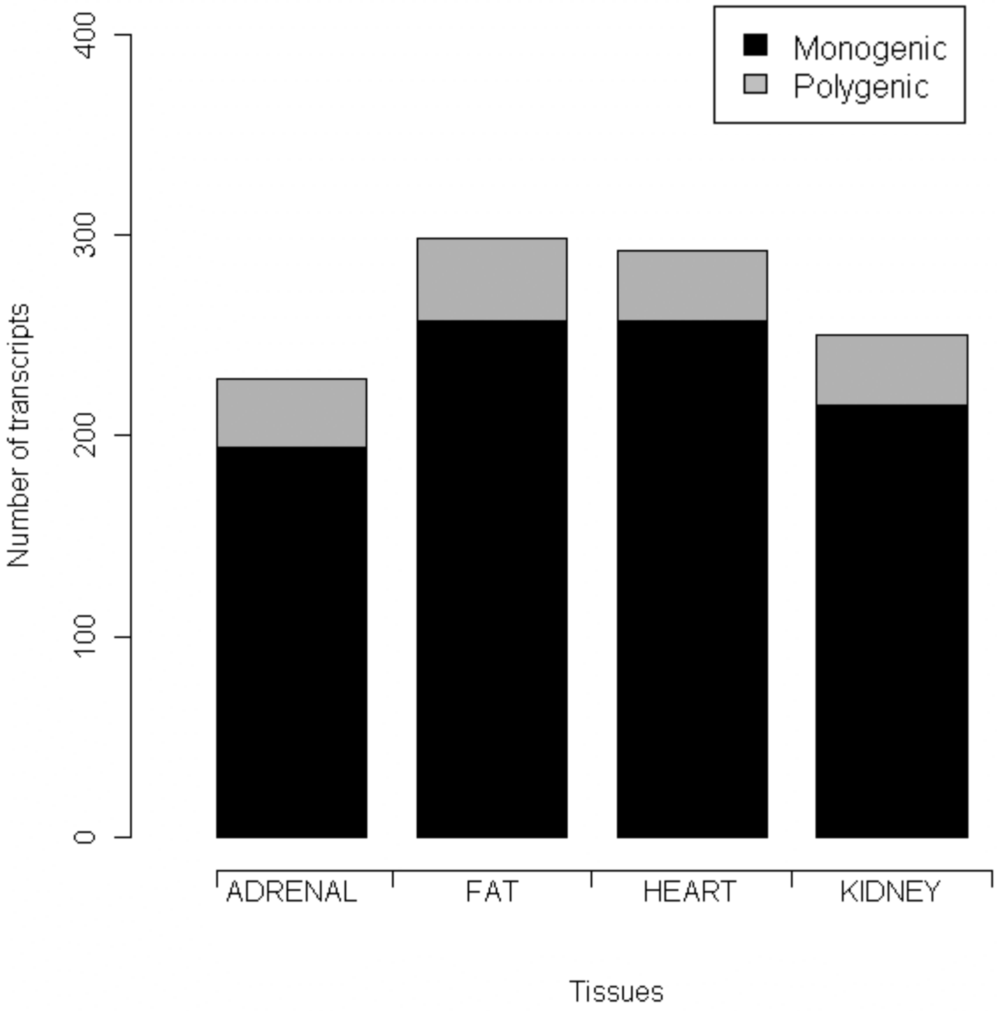

Figure 5. Number of transcripts that have one locus in common between SBR and SSM at FDR < 5% and that have the filtered best SBR model with multiple loci (polygenic control). No polygenic control was found by SSM at 5% FDR.

2.3 Comparison between SBR and QTL Reaper

eQTLs were also mapped using the QTL Reaper programs [9] which employs a standard regression approach and permutations to assess genome-wide corrected *p*-values (P_{GW}). Figure 6 below shows the number of transcripts found by the SBR model (FDR < 5% across all tissues), by QTL Reaper ($P_{GW} = 0.001$, FDR < 5% across all tissues) and those found in common by both methods. The SBR method found a greater number of significant transcripts as compared to QTL Reaper in each of the four tissues (~ 2 fold more eQTLs). The vast majority of transcripts found by QTL Reaper were also found by SBR. Figure 7 below shows the number of *cis*-acting, *trans*- acting and unknown eQTL in the transcripts found significant by both methods at 5% FDR. The majority of the commonly detected eQTLs are *cis* regulated (72% to 78%), 8% to 14% are *trans* regulated with the rest having unreliable positions. Both methods appear to have higher power in detecting *cis*-acting effects within individual tissues. Like the SSM method, QTL Reaper failed to detect any transcripts under polygenic control, while the SBR method found 12% to 20% of commonly detected transcripts to be under polygenic control. Figure 8 below shows the number of transcripts found by both the SBR model and QTL Reaper. The number of transcripts that the SBR model found to be under polygenic control and QTL Reaper found to be under monogenic control is given in grey and those which both methods found to be under monogenic control is give in black (Figure 8).

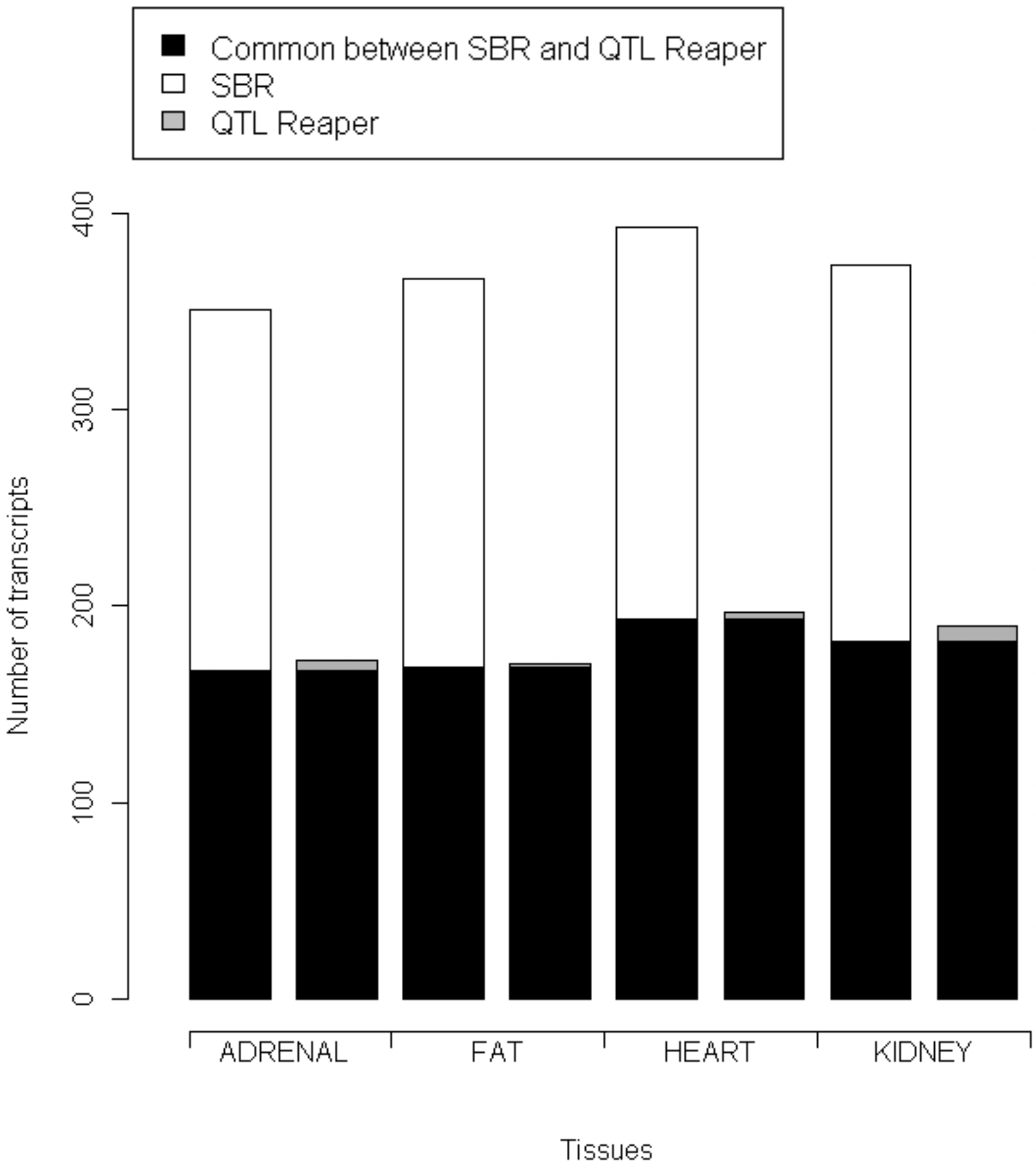

Figure 6. Number of significant transcripts found by SBR (FDR < 5%) and QTL Reaper (P_{GW} = 0.001, FDR < 5%). Transcripts were considered to be in common if one locus in the filtered best SBR model matched one locus found by QTL Reaper after removing redundant eQTLs which may result from linkage of expression values to multiple adjacent markers.

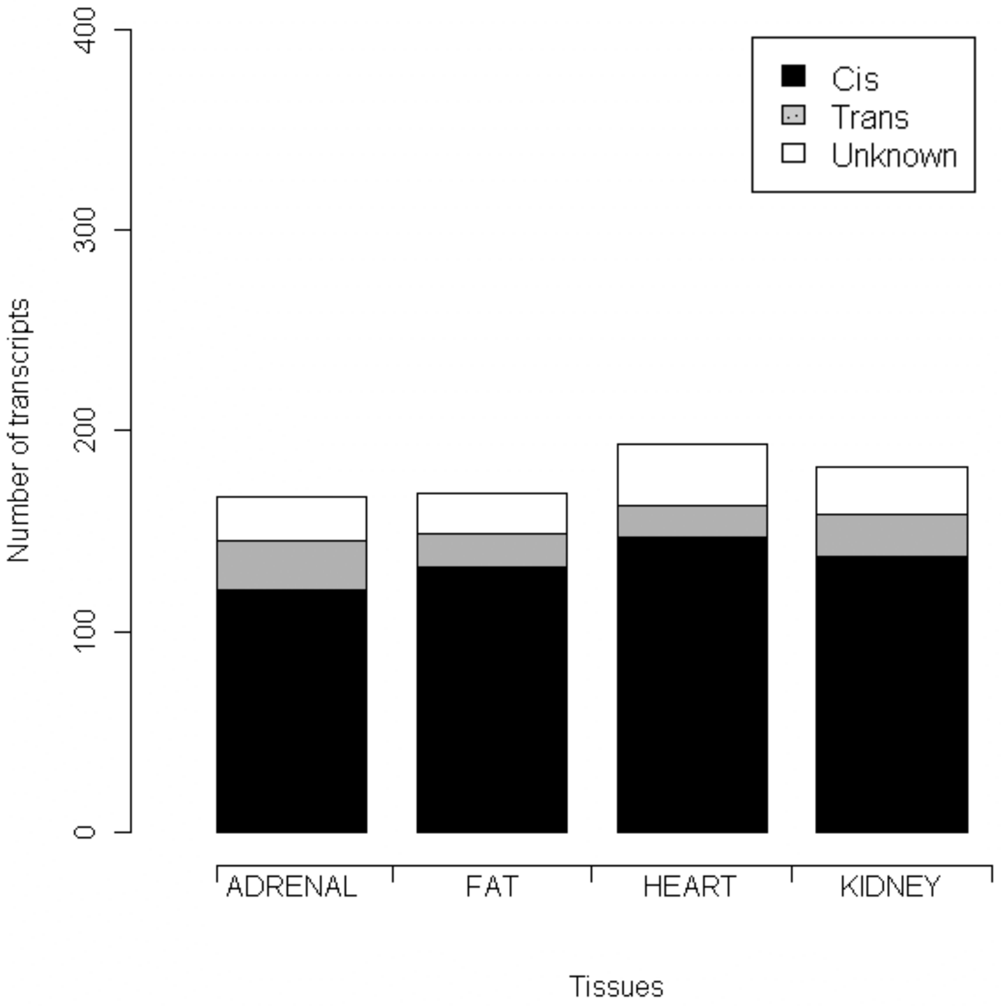

Figure 7. Number of *cis*-acting, *trans*-acting and unknown eQTLs found in common between SBR (FDR <5%) and QTL Reaper (P_{GW} = 0.001, FDR < 5%). Transcripts were considered to be in common if one locus in the filtered best SBR model matched one locus found by QTL Reaper after removing redundant eQTLs which may result from linkage of expression values to multiple adjacent markers. An eQTL was called *cis*-acting if it fell into a 10 Mb region around the localization of the transcript (see Materials and Methods).

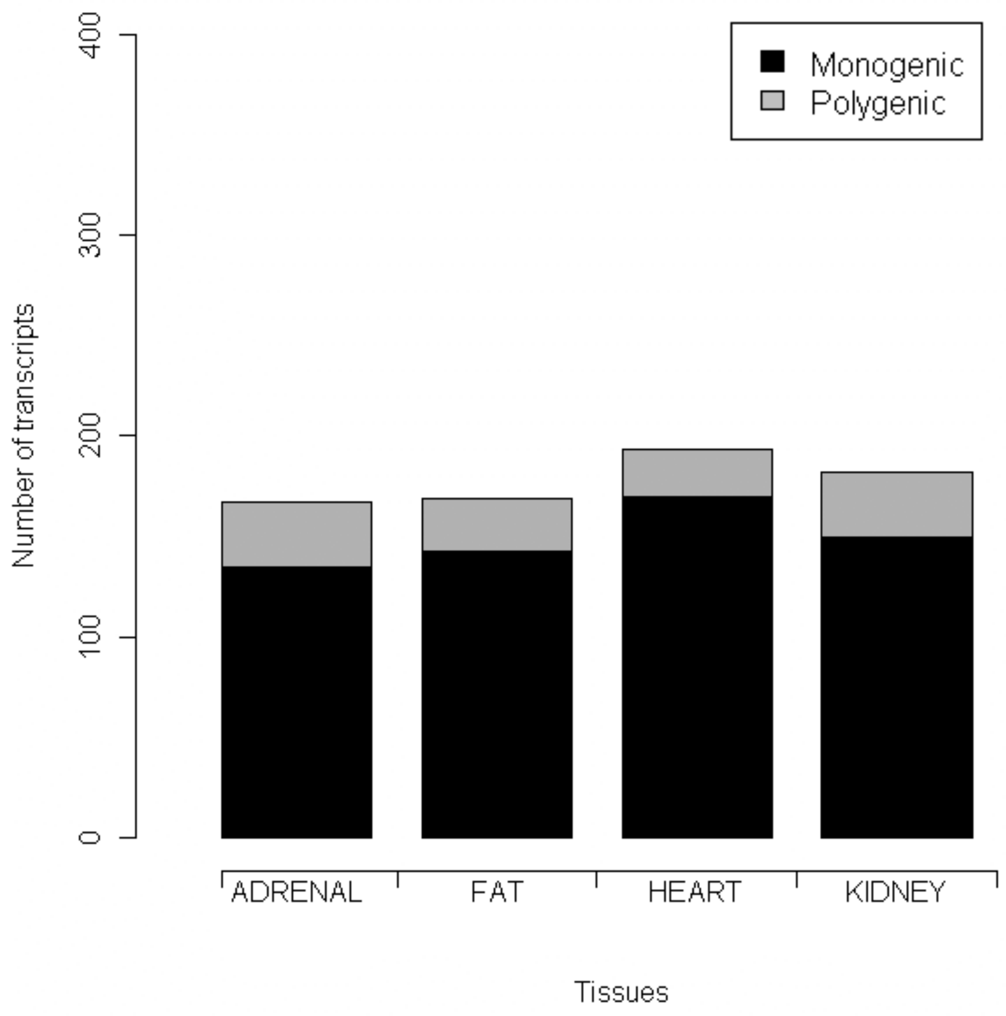

Figure 8. Number of transcripts that have one locus in common between SBR (FDR < 5%) and QTL Reaper (P_{GW} = 0.001, FDR < 5%) that have the filtered best SBR model with multiple loci (polygenic control). No polygenic control was found by QTL Reaper at 5% FDR.

2.4 Comparison between SBR, SSM and QTL Reaper

Figure 9 below shows the number of transcripts found in common between all three methods, SBR, SSM and QTL Reaper at 5% FDR. Both SBR and SSM found a greater number of significant transcripts than QTL Reaper and the vast majority of those found by QTL Reaper were also found by SBR and SSM. Figure 10 below shows the number of eQTL which are *cis*-acting, *trans*-acting and unknown for the transcripts found in common between the three methods, with the highest proportion being *cis*-acting (72% to 78%) and 8% to 15% being *trans*-acting. Figure 11 below shows the number of transcripts that SBR found to be under polygenic control out the transcripts found in common between all three methods. SBR found 13% to 18% of common transcripts to be under polygenic control (shown in grey), whereas the rest are under monogenic control (given in black).

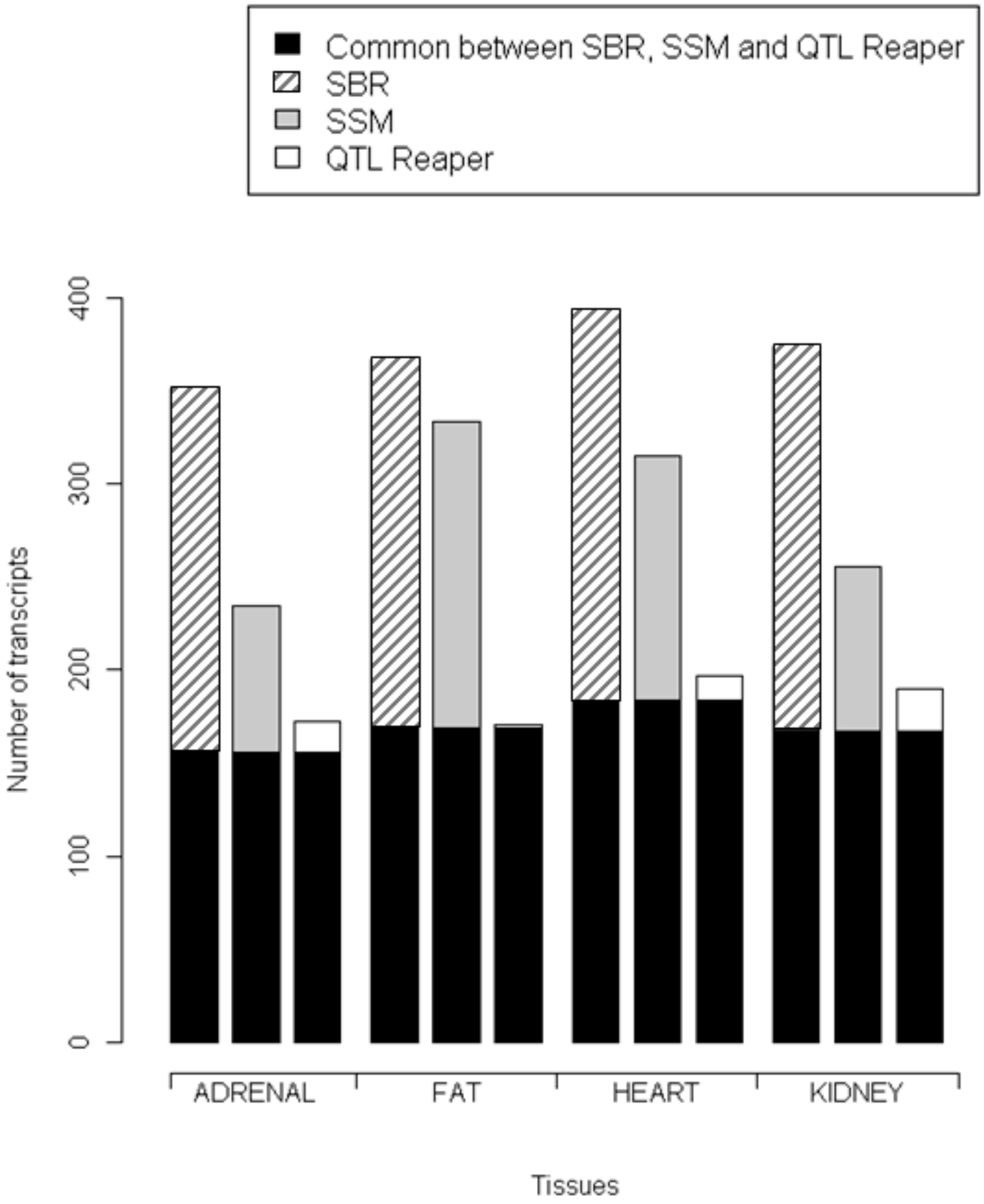

Figure 9. Number of significant transcripts found by SBR (FDR < 5%), SSM (FDR < 5%), and QTL Reaper (P_{GW} = 0.001, FDR < 5%) Transcripts were considered to be in common if one locus in the filtered best SBR model matched one locus found by SSM and QTL Reaper after removing redundant eQTLs which may result from linkage of expression values to multiple adjacent markers.

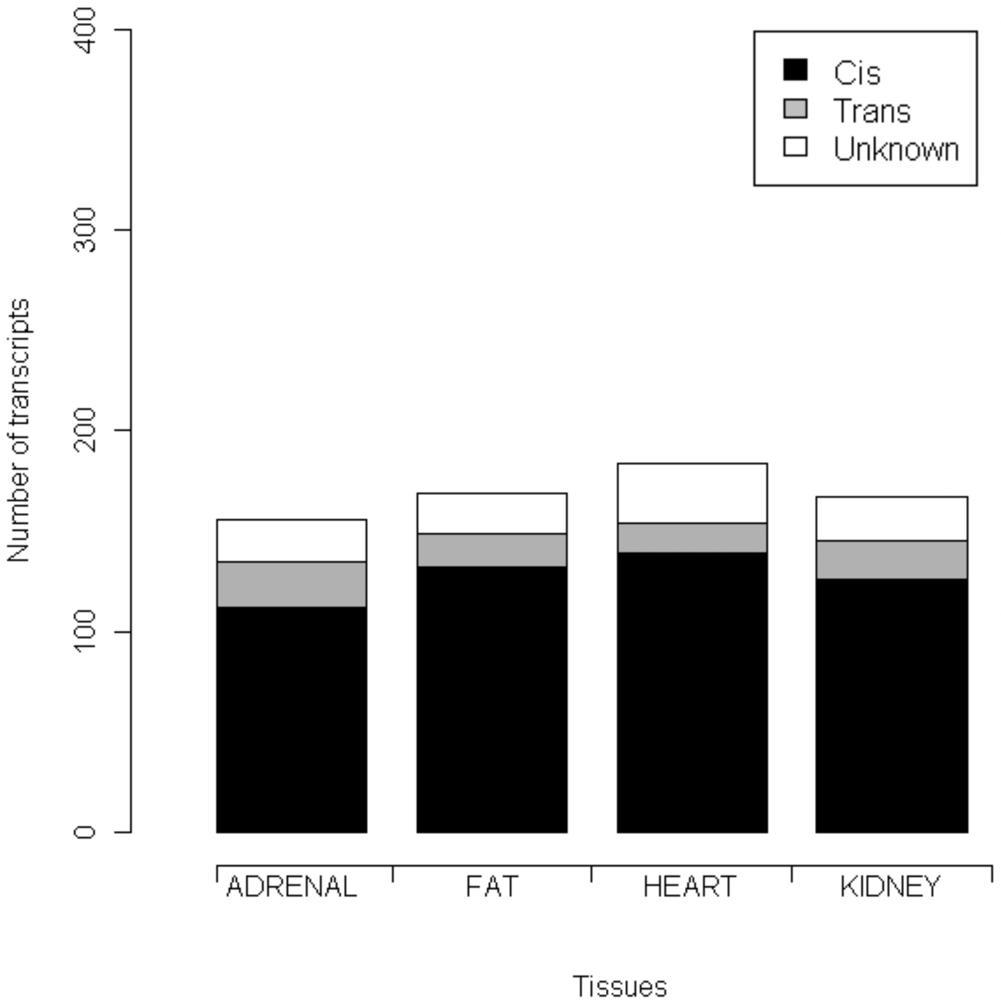

Figure 10. Number of *cis*-acting, *trans*-acting and unknown eQTLs found in common between SBR (FDR < 5%), SSM (FDR < 5%) and QTL Reaper ($P_{GW} < 0.001$, FDR < 5%). Transcripts were considered to be in common if one locus in the filtered best SBR model matched one locus found by SSM and QTL Reaper after removing redundant eQTLs which may result from linkage of expression values to multiple adjacent markers. An eQTL was called *cis*-acting if it fell into a 10 Mb region around the localization of the transcript (see Materials and Methods).

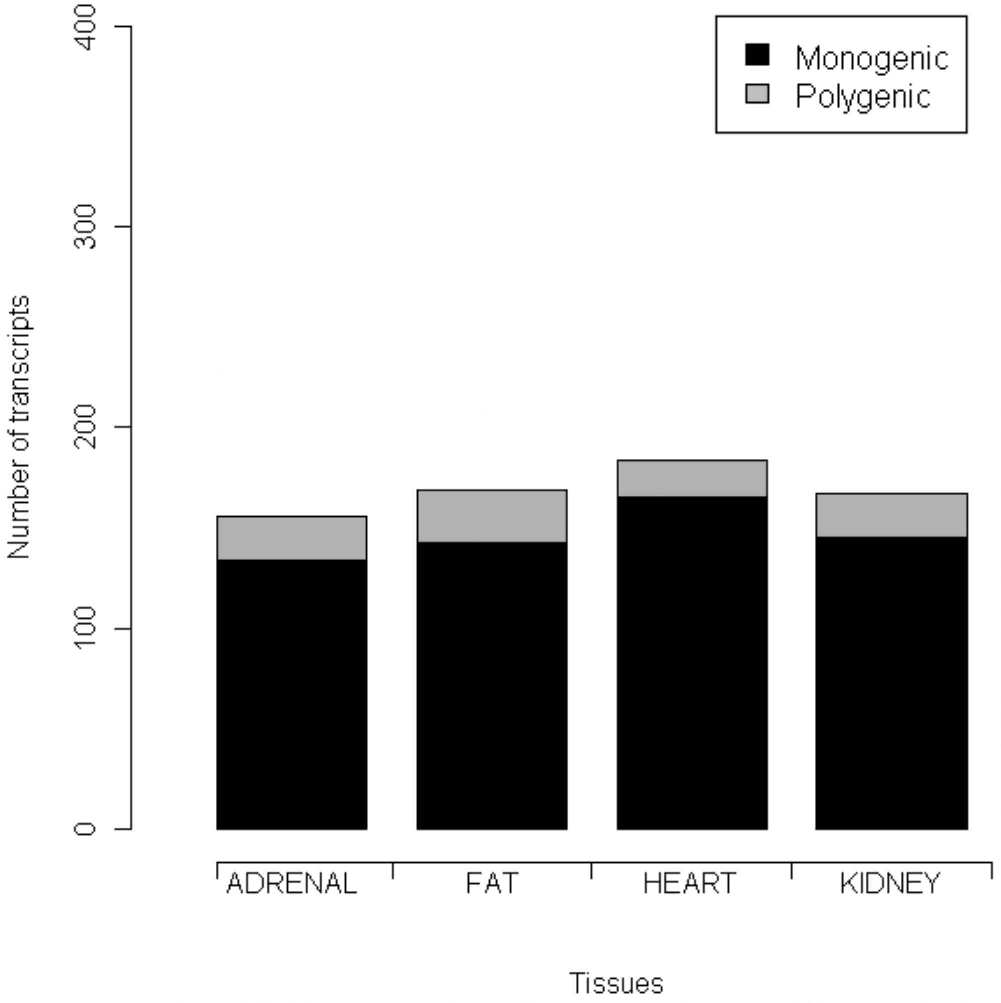

Figure 11. Number of transcripts that have one locus in common between SBR (FDR < 5%), SSM (FDR < 5%) and QTL Reaper (P_{GW} = 0.001, FDR < 5%) and that have the filtered best SBR model with multiple loci (polygenic control). No polygenic control was found by either SSM or QTL Reaper at 5% FDR.

2.5 Comparison between SBMR and Hotelling's T^2-test

The SBMR model identified 531 transcripts under common regulatory control across all tissues by one or more eQTLs, whereas the Hotelling's T^2-test found 459 transcripts at FDR < 5% (Table 1 below). An additional set of 72 transcripts were detected only by the SBMR, and 49 transcripts (69%) were under complex regulatory control by at least two eQTLs.

Table 1. Comparison between SBMR and the Hotelling's T^2-test

	SBMR		Hotelling's T^2-test	
Cis[1]	368	69%	369	80%
Trans[2]	137	26%	68	15%
undefined*	26	5%	22	5%
Total	531		459	

[1]For each transcript indentified by the SBMR or Hotelling's T^2-test we indicated whether there is at least one *cis*-eQTL, or one *cis*-eQTL and other *trans*-eQTL(s). [2]For each transcript indentified by the SBMR or Hotelling's T^2-test we indicated whether it mapped to at least one *trans*-eQTL. Percentages are calculated with respect to the total number of transcripts indentified within each analysis, i.e., 531 for SBMR and 459 for the Hotelling's T^2-test.

373 transcripts were found to be under common regulatory control in all tissues by both methods. When both methods find evidence for genetic regulation for the same transcript, we observed enrichment for *cis*-regulation (277 commonly detected *cis*-eQTLs, Table S6). Amongst the common set of transcripts, on average SBMR found more *trans*-eQTLs when compared with the Hotelling's T^2-test (Table 2 below).

Within the set of 373 transcripts detected by both methods, the percentage of polygenic regulation by $\geq$ 2 eQTLs was ~40% in the SBRM and only 16% in the Hotelling's T^2-test analysis (Figure 12 below). This suggests that although both methods found a large common set of probe sets under genetic control, the SBMR was more powerful to detect complex regulatory mechanism by multiple genetic control points.

Table 2. Transcripts found by both the SBMR and the Hotelling's T^2-test

	SBMR		Hotelling's T^2-test	
Cis[1]	291	78%	317	85%
Trans[2]	63	17%	37	10%
undefined*	19	5%	19	5%
Total	373		373	

[1]For each of the transcripts indentified by both the SBMR and Hotelling's T^2-test we indicated whether there is at least one *cis*-eQTL, or one *cis*-eQTL other *trans*-eQTL(s). [2]For each of the transcripts indentified by both the SBMR and Hotelling's T^2-test we indicated whether it mapped to at least one *trans*-eQTL. Percentages are calculated with respect to the total number of transcript indentified by both methods, i.e., 373.

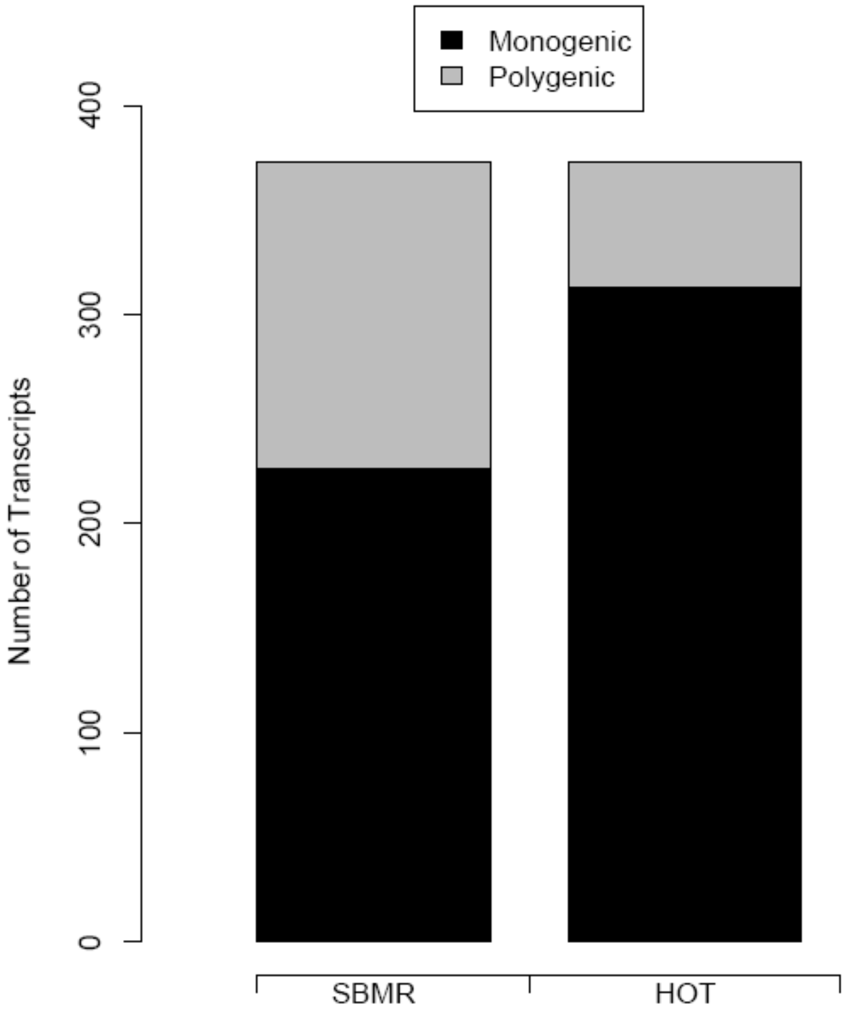

Figure 12. Number of transcripts under monogenic or polygenic control (≥ 2 eQTLs) found by the SBMR and Hotelling's T^2-test within the set of 373 transcripts (commonly detected by both approaches at FDR <5%). HOT, Hotelling's T^2-test.

In addition to the commonly detected 373 transcripts, the SBMR and the Hotelling's T^2-test identified a distinct set of 158 and 86 transcripts detected by one or the other method, respectively. The SBMR approach found that the 69% of the 158 transcripts were under complex regulation by two or more eQTLs, whereas only 2% of the 86 transcripts that were identified only by Hotelling's T^2-test showed polygenic regulation by ≥ 2 eQTLs (Figure 13 below).

Overall, the SBMR identified more transcripts under polygenic control than the Hotelling's T^2-test, and found 284 transcripts (14% of the total 2,000) with complex *trans*-acting genetic control, including 42 (2%) *trans*-acting eQTLs, 147 (7%) *trans*-acting eQTLs that are observed in combination with a *cis*-eQTL and 95 (~5%) models with multiple *trans*-eQTLs for the same transcript. In contrast, the Hotelling's T^2-test found only 68 transcripts (3% or the total 2,000) with complex *trans*-acting genetic control.

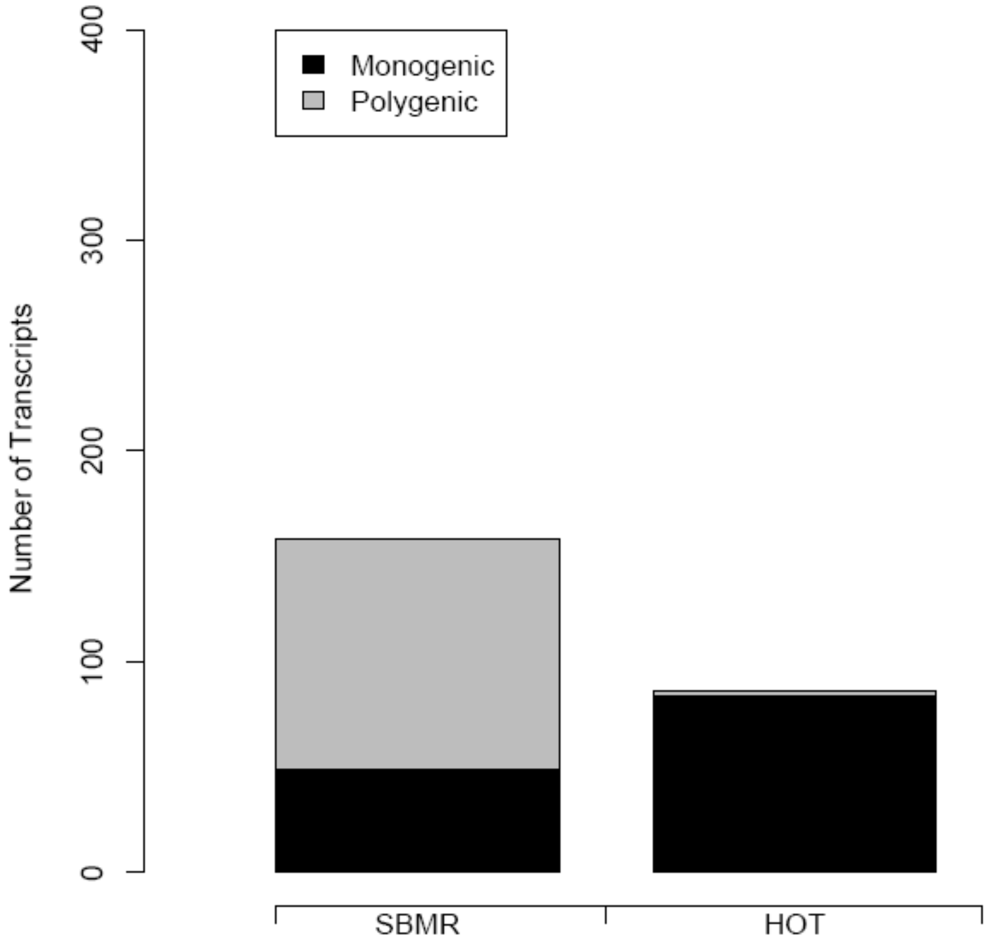

Figure 13. Number of transcripts found just by SBMR (158) or just Hotelling's T^2-test (86) at FDR <5% that are under monogenic or polygenic control by at least 2 eQTLs. HOT, Hotelling's T^2-test.

2.6 Power comparison with Hotelling's T^2-test and GFlasso

In order to assess the power of SBMR, we simulated six different cases: *i*) null model, i.e. no association; *ii*) single *cis*-acting eQTL model; *iii*) co-existing *cis*- and *trans*-acting regulation model; *iv*) a bigenic model with two *trans*-acting eQTLs; *v*) co-existing *cis*- and four *trans*-acting regulation model; *vi*) four *trans*-acting eQTLs. For each model we considered strong (pairwise correlation among the four tissues is between 0.95 and 0.90), medium (pairwise correlation is between 0.45 and 0.40) and low correlation pattern (pairwise correlation is no greater than 0.05). We simulated the gene expression level in the four tissues using equation (1) of main text, with a covariance matrix Σ derived from the correlation structure described above and a fixed marginal variance for each tissue equal to 0.25. We obtained the mean level XB multiplying the real maker data set X with a suitable matrix of regression coefficients B. In particular the (pleiotropic) *cis*-effect can assume values (4, 3, 2.5, 2), while the (pleiotropic) *trans*-effect has smaller size (1.5, 1.25, 1, 0.75) and closer to the variance level. For type-I error and power calculation, we simulated for each of the 18 cases (four genetic control models and three correlation structures), one hundred data sets. For each of the 1,800 data sets, we run SBMR, the Hotelling's T^2-test and a recently proposed generalised Lasso-type algorithm, the GFlasso algorithm [10], using the default specifications provided by the authors. For GFlasso, the output of the algorithm returns the non zero effects found for each tissue. Based on this, several definitions of an eQTL that is detected across tissues are possible, and for the presentation of results we defined a "detected eQTL" when GFlasso found at least one non-zero effect in one of the four tissues (see discussion below).

Table 3 below shows the type-I error for SBMR when the decision rule we adopted to select relevant markers was based on FDR level of 5% using the methods described in [4]. Type-I

error is very small in all the cases with a maximum value around 10^{-4} and with no error in the first four scenarios when the simulated correlation structure is very strong; a small type-I error is detected in the two highly polygenic models. In order to perform the power calculation, we fixed the error at 1×10^{-4} that corresponds to the maximum observed type-I error with 5% FDR decision rule. The greater power of SBMR is clear in all simulated cases and it decreases slightly as the correlation structure becomes weaker in the case of the highly polygenic models (case *v* and *vi*). As expected the Hotelling's T^2-test is competitive only when a single *cis*-acting eQTL is simulated. In the case of the polygenic models, since Hotelling's T^2-test is not multivariate in the predictors, it faces more difficulties in discovering the truth signal. The GFlasso is constantly outperformed, competing with SBMR just in the single *cis*-QTL scenario: its power constantly decreases (even faster than Hotelling's T^2-test) as more complex polygenic models are simulated. Note that these results are obtained even when we have adopted a "loose definition" of detected eQTL (i.e., at least a non zero effect in one of the four tissues) based on the estimated effects provided by the algorithm. Using a more stringent definition of detected eQTL (i.e., effects different from zeros in all tissues), we found an even lower power for the GFlasso. For the most difficult scenarios (co-existing *cis* and four *trans* eQTLs or four *trans* eQTLs with weak correlation) the SBMR power is around 45% and 63%, respectively, while both the Hotelling's T^2-test and GFlasso have less than half and about 1/5-1/10 of the SBMR power for case *v* and *vi*, respectively (Table 3).

Table 3. Power calculation for a fixed level of Type-I error for the SBMR, the Hotelling's T^2-test and the GFlasso [10].

		SBMR		Hotelling's T^2-test	GFlasso
Genetic model	*Correlation between tissues**	*Type-I error at 5% FDR*	*Power at 1×10^{-4} Type-I error*	*Power at 1×10^{-4} Type-I error*	*Power at 1×10^{-4} Type-I error*
Null	Strong	0	-	-	-
	Medium	0	-	-	-
	Weak	1.56×10^{-4}	-	-	-
One pleiotropic *cis-eQTL*	Strong	0	1.000	0.950	1.000
	Medium	1.30×10^{-5}	1.000	0.930	0.990
	Weak	1.30×10^{-5}	1.000	0.960	0.970
Pleiotropic *cis* and *trans*-eQTLs	Strong	0	0.935	0.475	0.570
	Medium	2.60×10^{-5}	0.920	0.485	0.540
	Weak	6.51×10^{-5}	0.965	0.465	0.550
Two pleiotropic *trans*-eQTLs	Strong	0	0.975	0.625	0.360
	Medium	6.51×10^{-5}	0.910	0.355	0.385
	Weak	9.11×10^{-5}	0.925	0.280	0.355
Pleiotropic *cis* and four *trans*-eQTLs	Strong	1.31×10^{-5}	0.640	0.355	0.155
	Medium	3.92×10^{-5}	0.485	0.245	0.145
	Weak	2.61×10^{-5}	0.445	0.185	0.173
Four pleiotropic *trans*-eQTLs	Strong	2.61×10^{-5}	0.810	0.076	0.056
	Medium	1.04×10^{-4}	0.656	0.133	0.050
	Weak	2.61×10^{-5}	0.630	0.116	0.056

*Strong: pairwise correlation among the four tissues is between 0.95 and 0.90; Medium: pairwise correlation is between 0.45 and 0.40; Weak: pairwise correlation is no greater than 0.05

3. Validation of microarray gene expression data

We conducted reverse transcription quantitative PCR (RT-QPCR) across all RI strains to validate the linkage data of two *cis*-regulated transcripts, two *trans*-regulated transcripts and one transcript representing the *Hopx* gene showing *cis* and *trans* regulation in the heart. In the case of *Hopx* and *EndoG*, cDNA was generated from left ventricle total RNA using iScript (Bio-Rad) and then amplified with SYBR Green JumpStart Taq Ready Mix (Sigma) with gene-specific primers (sequences below). In the case of *Irf7* and *Stat4*, one-step RT-PCR was carried out on total RNA with One-Step RT-PCR Master-Mix reagent (Applied biosystems) and predesigned Taqman probes (*Irf7* - Rn01450778_g1; *Stat4* – Rn01437242_m1) (Applied biosystems). The expression level of hypoxanthine phosphoribosyltransferase (HPRT) housekeeping gene was used for normalization. C_T values were analysed using the $2^{-\Delta\Delta C_T}$ method of Livak and Schmittgen [11]. Forward and reverse primers are given below.

**Gene**	**Forward primer**	**Reverse primer**
Hopx	AGGAGCAGACGCAGAAATGGT	CCGTGACCGATCTGCATTC
HPRT	TGACTATAATGAGCACTTCAGGGATTT	CGCTGTCTTTTAGGCTTTGTACTTG
EndoG	CCAATCACCGCTGGAGTCA	AGGCCCTGTGCAGACATAAAC

REFERENCES

1. Brown PJ, Vannucci, M. and Fearn, T (1998) Multivariate Bayesian variable selection and prediction. J R Statist Soc B 60: 627-641.
2. Gelman A, Carlin JB, Stern HS, Rubin DB (2004) Bayesian Data Analysis; Hall/CRC C, editor: CRC Press.
3. Denison DGT, Holmes, C.C., Mallick, B.K., Smith, A.F.M. (2002) Bayesian Methods for Nonlinear Classication and Regression: Wiley.
4. Chen W, Ghosh D, Raghunathan TE, Sargent DJ (2009) Bayesian Variable Selection with Joint Modeling of Categorical and Survival Outcomes: An Application to Individualizing Chemotherapy Treatment in Advanced Colorectal Cancer. Biometrics.
5. Storey JD, Akey JM, Kruglyak L (2005) Multiple locus linkage analysis of genomewide expression in yeast. PLoS Biol 3: e267.
6. Chipman H (1996) Bayesian Variable Selection with Related Predictors. Canadian Journal of Statistics 24: 17-36.
7. Storey JD (2002) A direct approach to false discovery rates. J R Statist Soc B 63: 479-498.
8. Petretto E, Mangion J, Dickens NJ, Cook SA, Kumaran MK, et al. (2006) Heritability and Tissue Specificity of Expression Quantitative Trait Loci. PLoS Genet 2.
9. Williams RB, Chan EK, Cowley MJ, Little PF (2007) The influence of genetic variation on gene expression. Genome Res 17: 1707-1716.
10. Kim S, Xing EP (2009) Statistical estimation of correlated genome associations to a quantitative trait network. PLoS Genet 5: e1000587.
11. Livak KJ, Schmittgen TD (2001) Analysis of relative gene expression data using real-time quantitative PCR and the 2(-Delta Delta C(T)) Method. Methods 25: 402-408.
