# Supplementary figures and images for: New Insights into the Genetic Control of Gene Expression using a Bayesian Multi-tissue Approach
Source: PLoS Comput Biol. 2010 Apr 8;6(4):e1000737. doi: 10.1371/journal.pcbi.1000737 (PMC2851562; doi:10.1371/journal.pcbi.1000737)

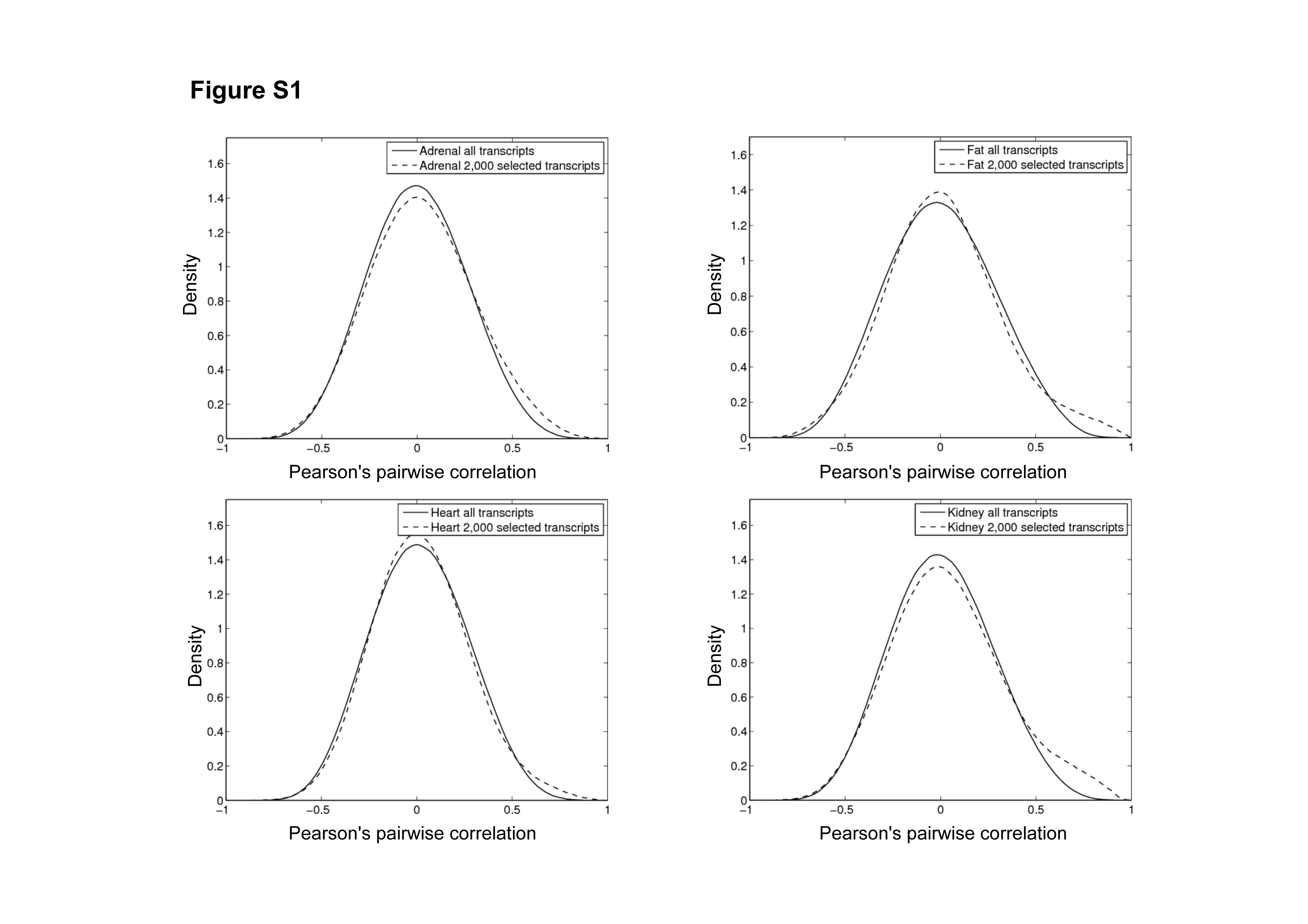

Supplement: Figure S1 — Correlation structure for the 2,000 transcripts that have the largest variation across tissues. Only 18 probe set pairs, whose Pearson's correlation is above 0.5, are common in the four tissues, while 102,932, 134,690, 82, 508 and 161,341 are the probe set pairs with Pearson's correlation above 0.5 in adrenal, fat, heart and kidney, respectively. This shows that the increment of the pairwise Pearson's positive correlation does not involve the same set of transcripts in the four tissues. (0.82 MB TIF) [file pcbi.1000737.s002.tif]

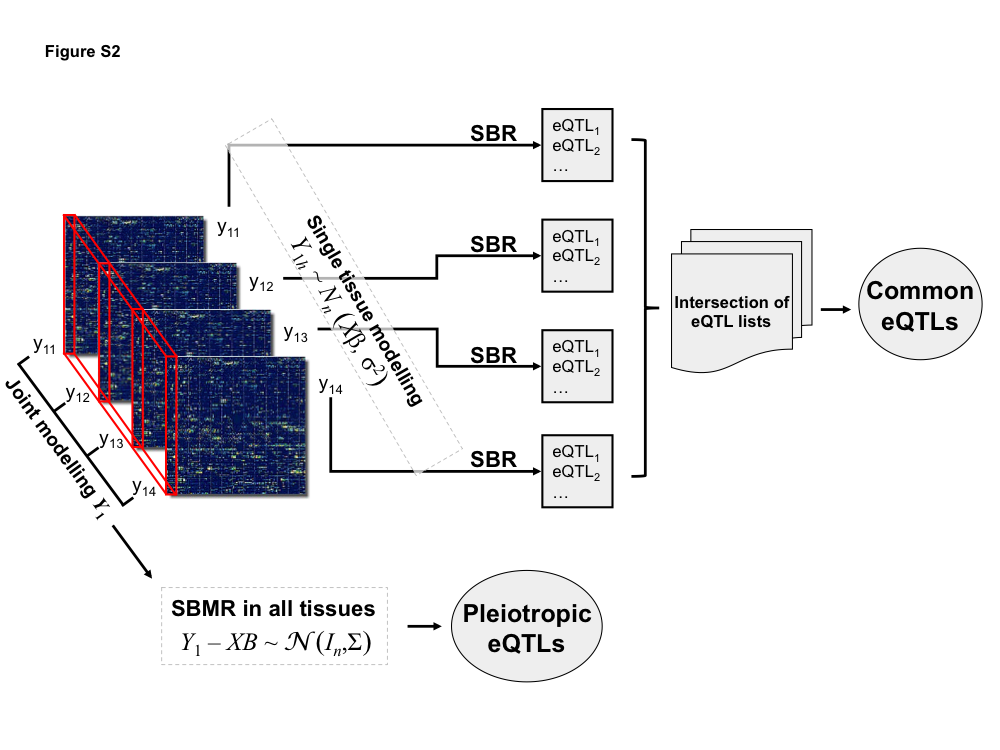

Supplement: Figure S2 — Overview of the Sparse Bayesian Regression (SBR) and Sparse Bayesian Multiple Regression (SBMR) approaches. In the SBR, mRNA levels (ygh, with g for the gth probe set and h for the hth tissue, respectively) are modelled at the level of each tissue, ygh∼Nn(Xβ,σ2), and the resulting eQTL lists are then compared to find common eQTLs across tissues. In the SBMR approach, mRNA levels of the same transcript measured in four tissues (Yg = [yg1, yg2, yg3, yg4]) are modelled jointly, Yg−XB∼N (In,Σ), and mapped to the genome to identify pleiotropic genetic control points of gene expression in all tissues. In the multiple tissues analysis the search for a set of markers that jointly predict the level of gene expression is complicated due to the fact that marginally each tissue can be potentially associated to a different group of covariates (mainly trans-effects) and share some others (mainly cis-effects). The SBMR approach is well powered to identify common genetic regulators even when they have moderate marginal effects. (3.00 MB TIF) [file pcbi.1000737.s003.tif]

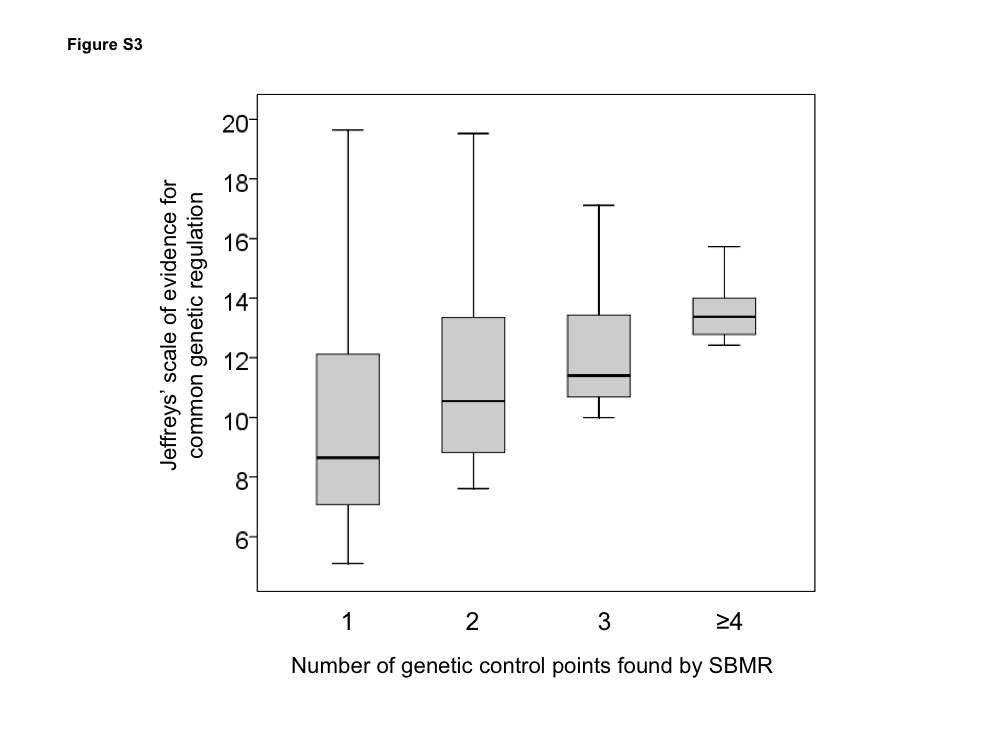

Supplement: Figure S3 — Distribution of log10 Bayes Factor for the best model visited for each transcript (y-axes) versus the number of distinct control points (x-axes) identified in each model after merging closely linked markers (see Materials and Methods). All 531 SBMR models were significant at <5% FDR threshold level, where this threshold was calculated taking into account the size of the best visited model (see Materials and Methods). On average, stronger evidence for common genetic control in all tissues is observed for high-dimensional models. (3.00 MB TIF) [file pcbi.1000737.s004.tif]

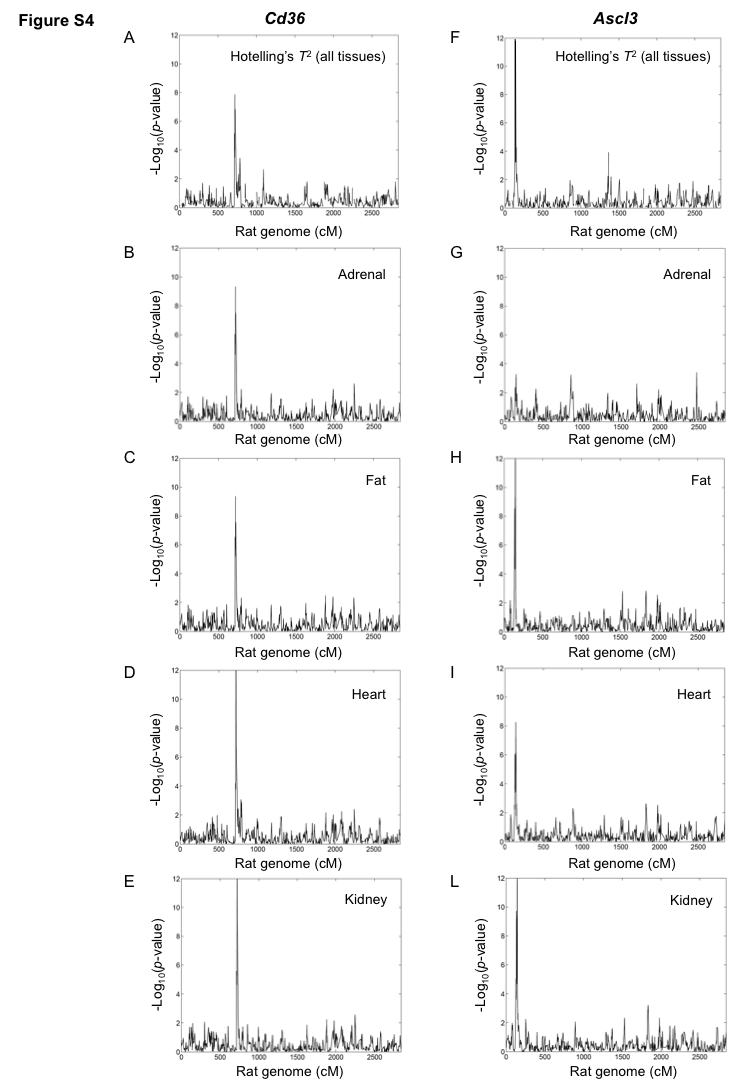

Supplement: Figure S4 — Genome-wide eQTL linkage results for Cd36 (A–E) and Ascl3 (F–L) genes in all tissues simultaneously using Hotelling's T2 test (top panels: A, F) and within individual tissues (panels B–E and G–L). For Cd36 gene the Hotelling's T2 test found common genetic regulation in all tissues at the Cd36 marker; this common eQTL is also detectable by intersecting the results from the single tissues analysis. For Ascl3 gene, the Hotelling's T2 test found the cis-eQTL on chromosome 1 (markers D1Rat55) but failed to detect the trans-eQTL on chromosome 7 (marker D7Mit8) at the 5% FDR level. The eQTL results from the individual tissue analysis did not find common cis- or trans-eQTLs, respectively. (3.25 MB TIF) [file pcbi.1000737.s005.tif]

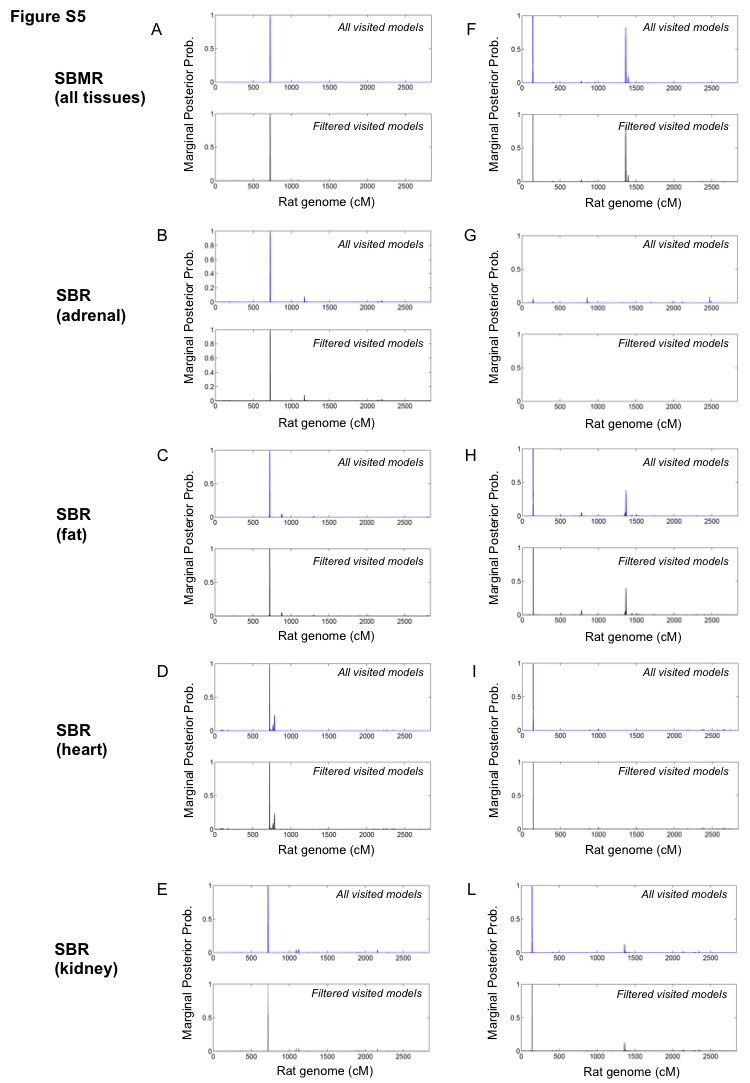

Supplement: Figure S5 — Marginal posterior probability of inclusion obtained from the SBMR and from the SBR analysis within individual tissues. We report the marginal posterior probability for all models visited (top panels) and for the filtered models (bottom panels) whose log10 Bayes Factor is above the selected cut-off (see Materials and Methods). (A–E) For Cd36 gene, the cis-regulatory control is consistently found using single tissue modelling (SBR) and the marginal posterior probability of inclusion corresponds to the filtered best model. (F–L) For Ascl3 gene, neither the cis-eQTL or the trans-eQTL was systematically detected by the SBR in all tissues, while the SBMR model identified both loci. In adrenal tissue, the filtered models did not show any genetic control points at FDR <5% (G, bottom panel). (3.25 MB TIF) [file pcbi.1000737.s006.tif]

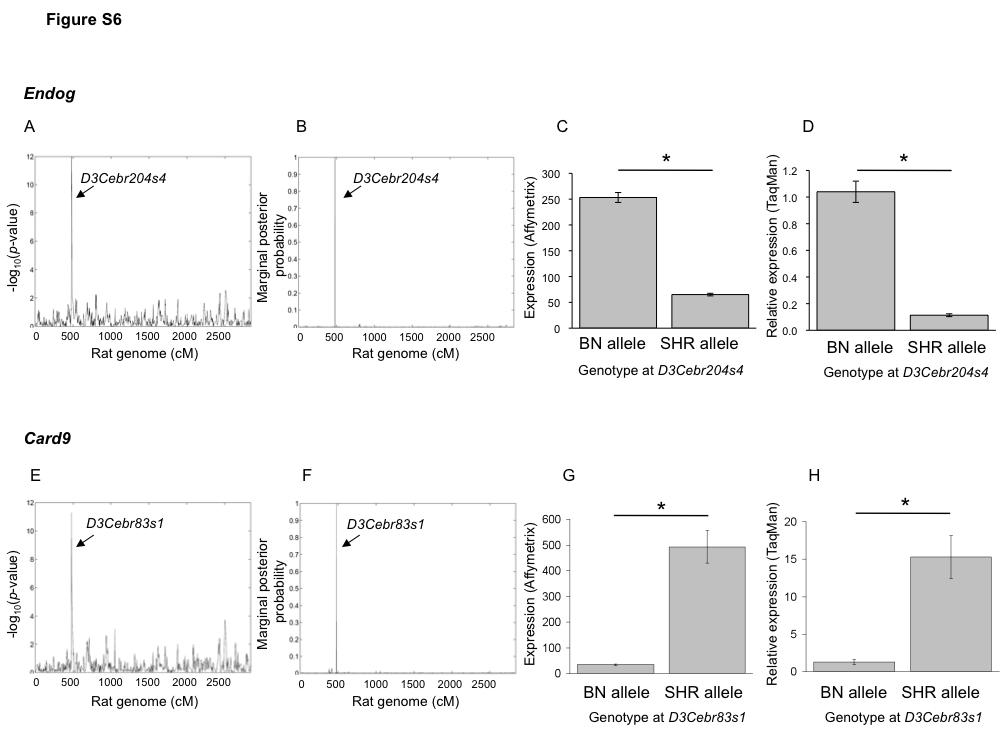

Supplement: Figure S6 — Validation of microarray gene expression linkages by RT-PCR. We replicated cis-eQTL linkages for: (A–D) Endog (Jeffreys' scale = 14.2) and (E–H) Card9 (Jeffreys' scale = 9.9), showing strong cis regulation in the heart tissue at markers D3Cebr204s4 and D3Cebr83s1, respectively. For each cis-eQTL we report the linkage results by t-test (panel A, E), by the SBR model (panel B, F), and expression values by BN and SHR genotype at the peak marker by microarray (panel C, G) and by RT-PCR (panel D, H). Expression data are reported as mean ± sem. Consistently with the microarray results, the RT-PCR data show significant evidence for cis-linkage for both genes. (*P<0.001) (3.00 MB TIF) [file pcbi.1000737.s007.tif]

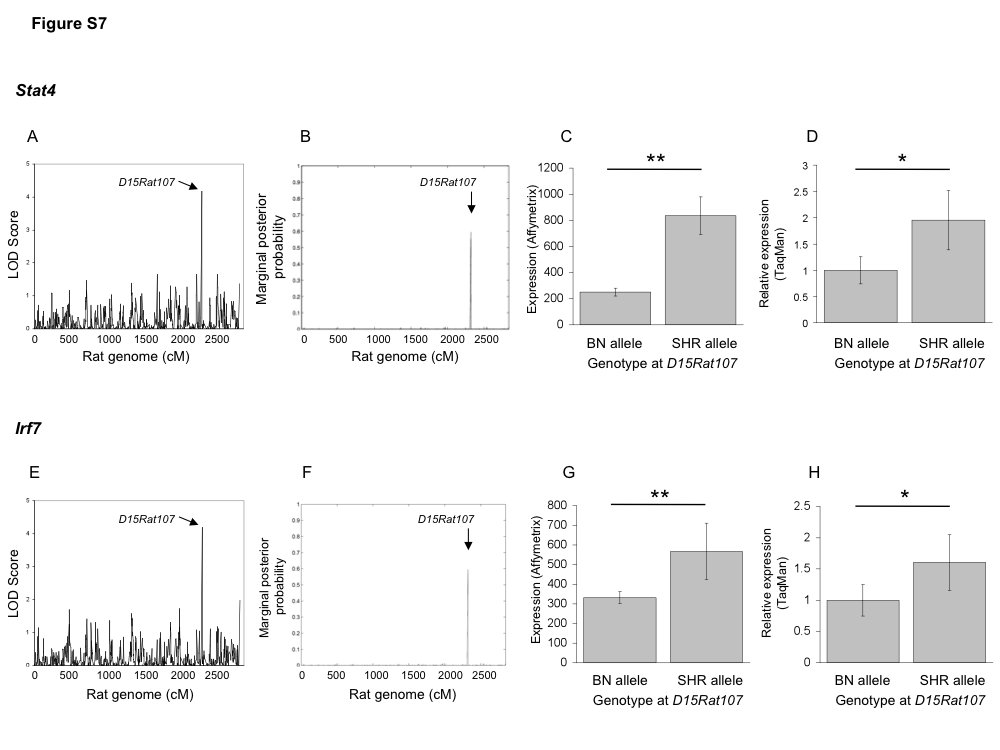

Supplement: Figure S7 — Validation of small-effect trans-eQTLs by RT-PCR. We replicated trans-eQTL linkages for: (A–D) Stat4 (Jeffreys' scale = 2.8) and (E–H) Irf7 (Jeffreys' scale = 2.7), both showing trans-acting regulation at marker D15Rat107 in the heart tissue with FDR <5%. For each trans-eQTL we report the linkage results by QTL Reaper (panel A, E), by the SBR model (panel B, F), and expression values by BN and SHR genotype at the peak marker (D15Rat107) by microarray (panel C, G) and by RT-PCR (panel D, H). QTL Reaper identified the trans-eQTL for Stat4 with genome-wide P-value (P GW) = 0.008 (FDR = 5%) and for Irf7 with P GW = 0.04 (FDR = 28%). For comparison, the SSM found trans-linkages for Stat4 and Irf7 at FDR = 5% and FDR = 17%, respectively. Expression data are reported as mean ± sem. Consistently with the microarray results, the RT-PCR data show significant evidence for trans-linkage for both genes. (*P<0.05, **P<0.01) (3.00 MB TIF) [file pcbi.1000737.s008.tif]
