# Supplementary material for: New Insights into the Genetic Control of Gene Expression using a Bayesian Multi-tissue Approach
Source: PLoS Comput Biol. 2010 Apr 8;6(4):e1000737. doi: 10.1371/journal.pcbi.1000737 (PMC2851562; doi:10.1371/journal.pcbi.1000737)
Supplement: Table S1 — Summary statistics of heritability of mRNA levels for the 2,000 transcripts considered in this study. (0.03 MB DOC) [file pcbi.1000737.s009.doc]

**Table S1.** Summary statistics of heritability of mRNA levels for the 2,000 transcripts considered in this study. Narrow-sense heritability of expression levels in the recombinant inbred lines was estimated using the method of Hegmann and Possidente^1^, h^2^_trait_ = 0.5V_A_/(0.5V_A_ + V_E_), where V_A_ represents the additive genetic component (variances of strain means) and V_E_ the average environmental component (variances within strains) as previously described^2,3^.

| Tissue | | | | |
| --- | --- | --- | --- | --- |
|  | Heart* | Fat* | Kidney* | Adrenal* |
| Minimum h^2^_trait_ | 0.03 | 0.07 | 0.03 | 0.05 |
| Maximum h^2^_trait_ | 0.96 | 0.88 | 0.94 | 0.95 |
| Median h^2^_trait_ | 0.24 | 0.25 | 0.19 | 0.28 |
| 25^ile^ of h^2^_trait_ | 0.15 | 0.19 | 0.11 | 0.17 |
| 75^ile^ of h^2^_trait_ | 0.37 | 0.34 | 0.35 | 0.43 |

*there was not a significant difference (Kolmogorov-Smirnov test) in the distribution of the heritability across tissues.

**References**

1. Hegmann, JP; Possidente, B. Estimating genetic correlations from inbred strains. *Behav Genet*. 1981;11:103–114.

2. Belknap, JK. Effect of within-strain sample size on QTL detection and mapping using recombinant inbred mouse strains. *Behav Genet*. 1998;28:29–38.

3. Petretto E, Mangion J, Dickens NJ, Cook SA, Kumaran MK, Lu H, Fischer J, Maatz H, Kren V, Pravenec M, Hubner N, Aitman TJ. Heritability and tissue specificity of expression quantitative trait loci. *PLoS Genet*. 2006 Oct 20;2(10):e172.
