# Supplementary material for: New Insights into the Genetic Control of Gene Expression using a Bayesian Multi-tissue Approach
Source: PLoS Comput Biol. 2010 Apr 8;6(4):e1000737. doi: 10.1371/journal.pcbi.1000737 (PMC2851562; doi:10.1371/journal.pcbi.1000737)
Supplement: Table S2 — Number of probe sets found to be under genetic control in the SBR and SBMR analyses (FDR 1% and 0.5%). (0.05 MB DOC) [file pcbi.1000737.s010.doc]

**Table S2.** Number of probe sets found to be under genetic control in the SBR and SBMR analyses (FDR 1% and 0.5%). We used “no eQTL” to identify probe sets whose best model was the null model (i.e., no evidence of genetic control) or when the best model visited with genetic control was not significant at FDR < 1% and < 0.5% (see Material and Methods). Polygenic models (≥ 2 eQTLs) are indicative of two or more distinct eQTLs (for the same probe set) that are located at least 10 cM far apart. Percentages were calculated in respect of the set of 2,000 transcripts considered in this study.

|  | *Number of genetic control points for the probe sets that are under genetic control (FDR <1%)* | | | | | | | |
| --- | --- | --- | --- | --- | --- | --- | --- | --- |
| *Analysis* | no eQTL | | 1 eQTL | | 2 eQTLs | | ≥ 3 eQTLs | |
| SBR in Fat | 1776 | (88.8%) | 184 | (9.2%) | 31 | (1.5%) | 9 | (0.5%) |
| SBR in kidney | 1788 | (89.4%) | 172 | (8.6%) | 29 | (1.4%) | 11 | (0.6%) |
| SBR in adrenal | 1853 | (92.6%) | 114 | (5.7%) | 25 | (1.3%) | 8 | (0.4%) |
| SBR in heart | 1800 | (90.0%) | 170 | (8.5%) | 23 | (1.2%) | 7 | (0.3%) |
|  |  |  |  |  |  |  |  |  |
| SBMR in all tissues | 1564 | (78.3%) | 248 | (12.4%) | 81 | (4.0%) | 107 | (5.4%) |
|  | *Number of genetic control points for the probe sets that are under genetic control (FDR <0.5%)* | | | | | | | |
| *Analysis* | no eQTL | | 1 eQTL | | 2 eQTLs | | ≥ 3 eQTLs | |
| SBR in Fat | 1792 | (89.6%) | 169 | (8.4%) | 31 | (1.6%) | 8 | (0.4%) |
| SBR in kidney | 1793 | (89.6%) | 168 | (8.4%) | 28 | (1.4%) | 11 | (0.6%) |
| SBR in adrenal | 1853 | (92.6%) | 114 | (5.7%) | 25 | (1.3%) | 8 | (0.4%) |
| SBR in heart | 1801 | (90.0%) | 169 | (8.4%) | 23 | (2.5%) | 7 | (0.4%) |
|  |  |  |  |  |  |  |  |  |
| SBMR in all tissues | 1759 | (88.0%) | 167 | (8.3%) | 50 | (2.5%) | 24 | (1.2%) |
