# Supplementary material for: New Insights into the Genetic Control of Gene Expression using a Bayesian Multi-tissue Approach
Source: PLoS Comput Biol. 2010 Apr 8;6(4):e1000737. doi: 10.1371/journal.pcbi.1000737 (PMC2851562; doi:10.1371/journal.pcbi.1000737)
Supplement: Table S3 — Comparison between SBR, SSM and QTL Reaper results. (0.06 MB DOC) [file pcbi.1000737.s011.doc]

**Table S3.** Comparison between SBR, SSM and QTL Reaper results.

| **Tissue** | **eQTL** | **SBR**  **(FDR < 5%)** | **SSM**  **(FDR < 5%)** | **Overlap between SBR & SSM (%)^1^** | **QTL Reaper**  ***P*_GW_ = 0.001** | **Overlap between SBR & QTL Reaper (%)^2^** | **Overlap between SBR, SSM & QTL Reaper (%)^3^** |
| --- | --- | --- | --- | --- | --- | --- | --- |
| **Adrenal** |  |  |  |  |  |  |  |
|  | *cis* | 223 | 164 | 162 (71%) | 125 | 121 (72%) | 112 (72%) |
|  | *trans* | 86 | 43 | 39 (17%) | 25 | 24 (14%) | 23 (15%) |
|  | unknown | 42 | 27 | 27 (12%) | 22 | 22 (13%) | 21 (13%) |
|  | Total | 351 | 234 | 228 | 172 | 167 | 156 |
| **Heart** |  |  |  |  |  |  |  |
|  | *cis* | 250 | 211 | 207 (71%) | 151 | 147 (76%) | 139 (76%) |
|  | *trans* | 85 | 55 | 41 (14%) | 16 | 16 (8%) | 15 (8%) |
|  | unknown | 58 | 49 | 44 (15%) | 30 | 30 (16%) | 30 (16%) |
|  | Total | 393 | 315 | 292 | 197 | 193 | 184 |
| **Kidney** |  |  |  |  |  |  |  |
|  | *cis* | 243 | 179 | 179 (72%) | 142 | 137 (75%) | 126 (75%) |
|  | *trans* | 79 | 42 | 37 (15%) | 24 | 21 (12%) | 19 (11%) |
|  | unknown | 51 | 34 | 34 (14%) | 24 | 24 (13%) | 22 (13%) |
|  | Total | 373 | 255 | 250 | 190 | 182 | 167 |
| **Fat** |  |  |  |  |  |  |  |
|  | *cis* | 208 | 207 | 198 (66%) | 134 | 132 (78%) | 132 (78%) |
|  | *trans* | 116 | 93 | 73 (24%) | 17 | 17 (10%) | 17 (10%) |
|  | unknown | 42 | 33 | 27 (9%) | 20 | 20 (12%) | 20 (12%) |
|  | Total | 366 | 333 | 298 | 171 | 169 | 169 |

**^1^** Number of eQTLs detected in common by the Sparse Bayesian Regression (SBR) and two-stage sequential search method (SSM). **^2^** Number of eQTLs detected in common by the SBR and eQTL Reaper methods. **^3^** Number of eQTLs detected in common by the SBR, SSM and eQTL Reaper methods. For each method a cut-off of 5% FDR was considered. In the QTL Reaper analysis this FDR cut-off corresponded to *P*_GW_ = 0.001 (Petretto et al. 2006). All percentages are calculated with respect to the total number of transcripts in common between the different methods considered.
