# Supplementary material for: New Insights into the Genetic Control of Gene Expression using a Bayesian Multi-tissue Approach
Source: PLoS Comput Biol. 2010 Apr 8;6(4):e1000737. doi: 10.1371/journal.pcbi.1000737 (PMC2851562; doi:10.1371/journal.pcbi.1000737)
Supplement: Table S4 — Polygenic models that have been detected in at least one tissue by the SBR model (FDR <5%). (0.10 MB PDF) [file pcbi.1000737.s012.pdf]

Table S4. Polygenic models that have been detected in at least one tissue by the SBR model (FDR <5%). For each probe set under polygenic control we indicate the probe set chromosome and the R^2 of the SBR model. We found 45 (out of 366, 12%) polygenic models in fat tissue, 41 (out of 373, 11%) in kidney, 40 (out of 351, 11%) in adrenal and 43 (out of 393, 11%) in heart tissue. Within each tissue, for each probe set we report the detected eQTLs, and for each eQTL we indicate: chromosome--genetic location (cM)--marker name. *Cis*-eQTLs are defined as having the genetic marker at the peak of linkage within 10 Mb of the location of the transcript and are highlighted in bold in the table.

			R^2 for the polygenic model				*cis* and *trans*-acting genetic regulation identified by the SBR models (≥2 eQTLs)			
Probeset ID	Probeset chr.	gene symbol	fat	kidney	adrenal	heart	fat	kidney	adrenal	heart
1372658_at	1	*Dmn*	-	-	-	93%				**1--117.1--D1Rat348**; 10--120.5--Ppy
1372846_at	1	*Cybasc3*	-	-	96%	-			**1--172.6--D1Rat293**; 5--71.3--D5Rat149; 7--106--D7Rat17; 16--25.9--D16Rat28	
1374907_at	1	--	90%	-	-		**1--136.7--D1Rat47**; 1--124.2--D1Rat42			
1374960_at	1	*RGD1564887*	94%	-	-	-	**1--223.1--D1Rat81**; 7--28.6--D7Utr1			
1374992_at	1	*Paf1*	-	-	-	75%				**1--73.4--D1Rat212**; 8--136.5--D8Ucsf1
1376249_at	1	*Fuca2*	-	86%	-	-		**1--0--D1Rat327**; 3--3.1--D3Ucsf1		
1376780_at	1	*RGD1310022*	91%	94%	-	94%	**1--127.6--D1Cebr103s1**; 6--8--D6Rat167	**1--127.6--D1Cebr103s1**; 5--88.8--D5Rat79		**1--127.6--D1Cebr103s1**; 2--12--D2Rat94
1377407_at	1	--	-	97%	-	-		**1--144.8--D1Cebr7s3**; 10--92.9--D10Wox13		
1387455_a_at	1	--	-	-	-	73%				13--71--D13Utr4; 15--21.8--D15Utr1
1387812_at	1	*Pace4*	79%	-	-	-	**1--114.7--D1Rat35**; 1--91.7--D1Rat30			
1387995_a_at	1	*Ifitm3*	75%	-	-	-	**1--154.2--D1Arb22**; 15--77.7--D15Rat107			
1388491_at	1	*March5*	-	-	74%	88%			**1--216.8--D1Cebrp29s6**; 19--109.1--D19Cebr204s28	**1--204.9--D1Rat304**; 15--21.8--D15Utr1; 16--113.9--D16Cebr48s1
1389650_at	1	--	83%	-	-	-	**1--124.2--D1Rat42**; 1--152.7--D1Rat69			
1367673_at	2	*Selenbp1*	-	85%	-	-		**2--155--D2Cebr104s1**; 4--49--D4Cebr46s5		
1370114_a_at	2	*LOC497750*	94%	-	-	-	**2--12--D2Rat94**; 3--1.7--D3Cebr83s1			
1371776_at	2	*Pik3r1*	94%	94%	94%	92%	**2--12--D2Rat94**; 3--1.7--D3Cebr83s1	**2--12--D2Rat94**; 3--1.7--D3Cebr83s1	**2--12--D2Rat94**; 3--1.7--D3Cebr83s1	**2--12--D2Rat94**; 3--1.7--D3Cebr83s1
1371924_at	2	*Olfml3*	72%	-	-	-	**2--161.3--Atp1a1**; 1--155.8--D1Rat292			
1372923_at	2	*Pex11b*	-	-	-	94%				**2--161.3--Atp1a1**; 7--167.8--D7Cebr46s1
1373610_at	2	--	-	-	74%	-			4--86.6--D4Rat35; 10--142.4--Dcp1	
1373810_at	2	*Pla2g12a*	-	77%	78%	-		**2--205.6--D2Rat61**; 8--41.3--D8Rat49	**2--210.7--D2Rat63**; 18--67--D18Rat61	
1374006_at	2	--	-	-	-	89%				**2--220--D2Rat66**; 4--141.7--Eno2; 20--66.2--D20Rat55
1374586_at	2	--	-	-	-	77%				**2--36.1--D2Rat202**; 11--7.7--D11Utr1
1387616_at	2	--	-	-	69%	-			3--27.8--D3Mit9; 11--30.7--D11Cebr15s1	
1369212_s_at	3	*Epb4.1l1*	-	92%	-	-		**3--196.8--D3Rat5**; 11--11.8--D11Cebr77s5		
1369352_at	3	*Hipk3*	-	-	79%	-	4--184.5--D4Cebr204s35; 6--39.2--D6Rat29		**3--2.4--D3Cebr204s4**; 19--81.8--D19Rat72	
1372289_at	3	*Slc25a12*	-	-	-	71%				**3--74.4--D3Rat180**; 9--12.9--D9Rat131
1373782_a_at	3	--	-	88%	-	-		**3--2.4--D3Cebr204s4**; 1--119.6--D1Rat270; 5--82.5--Pgm1		
1374921_at	3	*Rtel1*	95%	-	-	-	**3--234.4--Edn3**; 11--90.2--D11Rat1			
1376796_at	3	*Rab14*	-	-	99%	-			**3--11.2--D3Rat53**; 6--130.9--D6Utr5; 7--116.9--D7Rat131; 13--88.3--Fh; 19--25.9--D19Rat52	
1376868_at	3	--	-	-	-	72%				4--2.9--D4Ucsf1; 16--5.8--D16Rat51
1387170_at	3	*Csnk2a1*	-	-	-	89%				**3--189.5--D3Mit3**; 11--4.6--D11Rat20
1389142_at	3	*Pldn*	-	91%	-	-		**3--120.9--D3Rat257**; 3--101.1--D3Mit15; 15--21.8--D15Utr1		
1389668_at	3	*Spbc25*					3--120.9--D3Rat257; 10--84.7--D10Rat31			
1390717_at	3	*RGD1311037*	-	89%	-	-		**3--124.4--D3Rat159**; 15--24.6--D15Rat6		
1370075_at	4	*Dhfr*	-	96%	-	-		2--7.4--D2Rat124; 10--4.3--D10Cebr27s2; 19--50.2--D19Rat14		
1370155_at	4	*Col1a2*	78%	-	-	-	**4--133--D4Rat240**; 16--22.3--D16Rat75			
1370806_at	4	*Retsat*	-	-	-	98%				**4--86.6--D4Rat35**; 7--81.8--D7Cebr204s12
1372583_at	4	--	-	-	74%	-			**4--190.5--Pthlh**; 1--41.7--D1Rat15	
1373611_at	4	--	72%	-	-	-	2--213.4--D2Rat247; 11--27.8--D11Mit2			
1374496_at	4	--	-	73%	-	-		7--87.9--D7Mit5; 11--27.8--D11Mit2		
1376808_at	4	--	-	-	-	75%				**4--76--D4Cebrp215s9**; 6--126.8--D6Rat79
1387710_at	4	--	-	74%	-	-		1--127.6--D1Cebr103s1; 2--204.8--D2N35		
1388493_at	4	*AW146242*	89%	-	-	-	**4--79.2--D4Cebrp1016s14**; 3--211.8--D3Cebr80s2; 12--23.4--D12Mit7			
1389234_at	4	*Vwf*	81%	-	-	-	**4--145.6--D4Rat66**; 17--11.2--D17Mit7			
1367949_at	5	*Penk-rs*	-	-	89%	-			**5--5--D5Rat188**; 16--71.5--D16Rat60	
1368317_at	5	*Aqp7*					1--136--Rt6; 4--133--D4Rat240			
1371782_at	5	*Nipsnap3a*	-	-	78%	-			**5--45.8--D5Rat228**; 4--39.6--D4Rat16	
1371960_at	5	--	94%	-	92%	96%	**5--116.2--D5Rat38**; 1--220.7--D1Utr7		**5--116.2--D5Rat38**; 1--154.2--D1Arb22	**5--116.2--D5Rat38**; 1--154.2--D1Arb22; 1--193.9--Jak2
1372475_at	5	*Pink1*	-	-	-	97%				8--53.9--Grik4; 14--12.8--D14Utr6
1376628_at	5	*Zfp189*	-	-	84%	-			**5--33.4--D5Rat140**; 8--80.5--D8Rat150	
1368859_at	6	*Ppm1a*	-	-	92%	-			**6--75.2--D6Cebr36s1**; 3--3.1--D3Ucsf1; 10--79--Srebp1	
1369973_at	6	*Xdh*	-	-	94%	-			**6--19.1--D6Rat147**; 12--39.3--D12Rat14	
1370829_at	6	*Fntb*	-	-	94%	-			**6--85.1--D6Rat87**; 16--1.7--D16Rat54; 16--11.1--D16Utr1	
1376268_at	6	*Arf6*	-	-	79%	88%			6--38.3--D6Rat36; 6--99.1--D6Rat117	**6--75.2--D6Cebr36s1**; 13--69.4--D13Utr7
1377329_at	6	*Bzw2*	-	-	-	94%				**6--58.1--D6Mit4**; 1--103.2--D1Arb11
1387344_at	6	--	-	-	-	72%				4--54.3--D4Cebrp145s3; 16--32.3--D16Rat67
1389334_at	6	*LOC316632*	95%	-	-	-	5--92.9--Slc2a1; 9--107.2--Alp1; 10--49.8--D10Cebrp1016s2; 15--2.7--D15Rat1			
1372754_at	7	--	-	-	-	73%				3--75.9--D3Rat37; 9--93.7--Inha
1374915_at	7	--	-	93%	97%	-		**7--120.8--D7Cebr77s1**; 10--1.5--D10Rat218	**7--120.8--D7Cebr77s1**; 12--21.1--D12Rat28	
1382778_at	7	*Dusp6*	92%	-	-	94%	**7--28.6--D7Utr1**; 13--82.6--D13Mit4			**7--28.6--D7Utr1**; 1--206.5--D1Rat194; 1--223.1--D1Rat81
1387819_at	7	*Ela1*	90%	-	-	-	7--39--D7Rat103; **7--173.2--D7Cebr205s1**; 8--147.8--D8Cebr46s6			
1389332_at	7	--	-	-	-	73%				1--107.3--D1Cebr72s1; 4--49--D4Cebr46s5
1393130_at	7	*Txn2*	-	-	-	92%				**7--120.8--D7Cebr77s1**; 10--11.5--D10Rat182; 10--34.9--D10Ntr32
1369171_at	8	*Mst1*	97%	-	94%	-	**8--140--D8Rat202**; 11--27.8--D11Mit2		**8--140--D8Rat202**; 1--41.7--D1Rat15; 7--172.5--D7Cebr6s5	
1369200_at	8	*Nt5e*	-	-	-	75%				**8--120.9--D8Rat130**; 11--11.8--D11Cebr77s5
1369665_a_at	8	*Il18*	-	83%	-	-		**8--81.6--Rt5**; 15--2.7--D15Rat1		
1371442_at	8	*Hyou1*	-	-	95%	90%			**8--53.2--D8Mit3**; 2--127.5--D2Rat222; 3--156.9--D3Cebr37s29	**8--52.5--Scnb2**; 3--127.6--D3Rat157
1373159_at	8	--	-	95%	-	73%		**8--133.1--Rbp2**; 6--116.6--D6Cebrp165s2		**8--143--Mylc1v**; 13--81.2--D13Utr1
1374583_at	8	*Dcps*	97%	97%	98%	98%	**8--45.2--Es6**; 13--45.2--D13Rat62	**8--49--D8Rat219**; 6--12--D6Cep8	**8--42.8--Kcnj1**; 10--98--D10Rat133	**8--49--D8Rat219**; 6--12--D6Cep8
1377452_at	8	*Clec3b*	-	83%	-	-		**8--159.8--D8Cebr16s5**; 1--155.8--D1Rat292		
1382997_at	8	*Slc22a14*	-	82%	-	-		**8--156.7--D8Rat81**; 1--135.1--D1Rat277		
1385386_at	8	*RGD:628754*	-	-	-	96%				**8--53.5--D8Mit4**; 8--118--D8Rat135
1390185_at	8	*Dcps*	-	97%	-	-		**8--53.5--D8Mit4**; 13--45.2--D13Rat62		
1398460_at	8	*RGD1311723*	95%	-	-	-	**8--5.6--D8Rat56**; 8--153.7--D8Cebrp203s1			
1370033_at	9	--	-	-	-	72%			3--127.6--D3Rat157; 4--37.6--D4Rat156	
1370959_at	9	*Col3a1*					**9--43.4--D9Rat23**; 4--135.6--Cacna1s			
1373463_at	9	--	69%	-	-	-	10--39.5--D10Utr5; 18--60--D18Rat50			
1374196_at	9	*Lancl1*	-	97%	-	95%		**9--88.4--Cryga**; 5--29.3--D5Cebr63s1; 10--2.2--D10Utr3		**9--88.4--Cryga**; 8--55.5--Thy1
1384309_at	9	--	-	-	83%	98%			**9--86.8--D9Rat15**; 5--90.4--D5Rat30	**9--72.9--D9Rat156**; 2--170--D2Cebr10s6; 11--90.2--D11Rat1
1387376_at	9	*Aox1*	-	91%	-	-		**9--69.7--D9Rat93**; 1--128.3--C		
1388926_at	9	*Enpp5*	-	77%	-	-		**9--12.9--D9Rat131**; 1--144.1--D1Cebr21s2		
1367562_at	10	*Sparc*	-	76%	-	-		**10--58--D10Cebr4s9**; 8--66.9--Crabp1		
1367989_at	10	*Slc2a4*	-	91%	-	-		**10--95.8--D10Rat80**; 2--177.2--D2Rat54; 20--46.4--D20Rat23		
1369983_at	10	*Ccl5*	74%	-	-	-	19--42.1--D19Ucsf2; 19--50.2--D19Rat14			
1370705_at	10	*Zfp597*	-	-	96%	-			**10--2.2--D10Utr3**; 10--95.8--D10Rat80	

			R^2 for the polygenic model				*cis* and *trans*-acting genetic regulation identified by the SBR models (≥2 eQTLs)											
Probeset ID	Probeset chr.	gene symbol	fat	kidney	adrenal	heart	fat			kidney			adrenal			heart		
1371700_at	10	--	-	-	-	86%										2--140.5--D2Cebr28s4	2--153.1--D2Mit12	
1374888_at	10	*Ccdc49*	93%	-	-	-	**10--118--D10Rat145**	1--217.6--D1Utr5										
1375519_at	10	*LOC287167*	88%	-	-	-	**10--5.3--D10Mit6**	1--64.1--D1Cebrp131s2										
1375540_at	10	*RGD1308696*	-	-	92%	-							**10--51.1--D10Mit4**	1--241.9--D1Cebrp37s18	6--112.7--D6Rat184			
1388116_at	10	*Col1a1*	77%	-	-	-	4--133--D4Rat240	13--67.2--D13Mit5										
1389935_at	10	*RGD1309310*	-	-	83%	-							**10--179.5--D10Rat7**	4--141.7--Eno2				
1372438_at	11	*Nit2*	-	-	-	93%										**11--36--D11Rat7**	13--82.6--D13Mit4	
1387036_at	11	--	-	73%	-	-				4--62--D4Rat102	8--53.9--Grik4							
1388879_at	11	*Abi3bp*	-	-	93%	-							**11--36--D11Rat7**	17--11.9--D17Ucsf2				
1375958_at	12	--	-	97%	93%	-				**12--25.1--Hsp27**	16--1.7--D16Rat54		**12--25.1--Hsp27**	16--1.7--D16Rat54				
1376550_at	12	--	93%	-	-	-	**12--15.8--D12Mit5**	1--114.7--D1Rat35										
1376840_at	12	--	-	93%	-	-				**12--5.6--D12Cebrp97s9**	1--144.1--D1Cebr21s2							
1377082_at	12	--	-	-	-	93%										**12--25.1--Hsp27**	16--1.7--D16Rat54	
1381336_at	12	--	-	78%	-	-				**12--3.1--D12Cebr4s3**	14--47.3--D14Cebrp136s2							
1387272_at	12	*Eif2ak1*	-	-	80%	-							**12--12.4--D12Rat61**	10--142.4--Dcp1				
1390701_at	12	*MGC94190*	-	96%	-	95%				**12--40.1--D12Cebr1s1**	19--112--D19Rat103					**12--23.4--D12Mit7**	2--121.7--D2Rat147	4--169--D4Cebr9s4
1392941_at	12	--	-	-	76%	-							**12--22.6--Pai1**	4--156.9--D4Rat68				
1371732_at	13	*Dpt*	86%	-	-	-	**13--64.7--D13Rat131**	15--30.5--D15Utr3										
1373661_a_at	13	*Cxcr4*	91%	-	-	-	**13--30--Ren**	10--131.1--D10Rat267	12--12.4--D12Rat61									
1375428_at	13	*Creg1*	86%	-	-	-	**13--64.7--D13Rat131**	11--12.7--D11Cebr204s16	17--71.4--D17Mit6									
1387074_at	13	--	72%	-	-	-	17--37.1--D17Rat144	19--115--D19Rat5										
1389521_at	13	*Ivns1abp*	-	88%	-	-				**13--41.3--D13Cebr2s5**	10--49.8--D10Cebrp1016s2							
1367816_at	14	*Hod*	-	-	-	90%										**14--35.5--D14Rat36**	2--94.9--D2Rat136	
1386881_at	14	--	-	-	71%	-							3--11.2--D3Rat53	10--39.5--D10Utr5				
1398390_at	14	*LOC498335*	74%	-	-	-	**14--12.8--D14Utr6**	1--0--D1Rat327										
1371963_at	15	*Pcca*	-	-	-	79%										**15--77.7--D15Rat107**	1--155.8--D1Rat292	
1375530_at	15	*Gnpnat1*	-	82%	-	-				**15--16.3--D15Mit3**	7--167.8--D7Cebr46s1							
1388654_at	15	*RGD:1309297*	-	-	99%	-							**15--24.6--D15Rat6**	1--46.3--D1Cebr68s1				
1374207_at	16	*RGD:621861*	-	90%	-	-				10--22.2--D10Cebrp207s1	10--86.2--D10Rat102	14--59.3--D14Rat37						
1388508_at	16	*Ap1m1*	92%	-	-	-	**16--11.1--D16Utr1**	3--213.4--D3Cebr80s1										
1388688_at	16	--	-	-	-	88%										**16--112.2--D16Rat15**	8--113.1--D8Utr5	18--105.5--D18Cebr51s1
1388909_at	16	*Oxnad1*	-	95%	79%	-				**16--1.7--D16Rat54**	1--147.1--D1Rat287	8--133.1--Rbp2	**16--1.7--D16Rat54**	6--99.1--D6Rat117				
1389722_at	16	--	-	-	89%	-							**16--112.2--D16Rat15**	8--113.1--D8Utr5	18--105.5--D18Cebr51s1			
1389793_at	16	--	89%	-	83%	-	**16--11.1--D16Utr1**	17--11.9--D17Ucsf2					**16--11.1--D16Utr1**	13--12.8--D13Cebr9s3				
1398245_at	16	--	-	-	85%	-							8--113.1--D8Utr5	9--69.7--D9Rat93	10--44.8--D10Rat71			
1368225_at	17	*Exoc2*	-	-	-	86%										**17--37.1--D17Rat144**	6--25.2--D6Rat171	
1372000_at	17	*Net1*	-	79%	-	-				**17--60.6--D17Rat151**	10--9.9--D10Rat121							
1385248_a_at	17	*Ogn*	88%	-	-	-	**17--9.6--D17Ucsf1**	4--135.6--Cacna1s	5--16.7--D5Rat126									
1388603_a_at	17	*Hbld2*	-	-	-	85%										**17--11.9--D17Ucsf2**	4--31.7--D4Rat252	
1390450_a_at	17	--	-	-	-	74%										16--25.2--D16Mit3	20--90.3--D20Arb249	
1376891_at	18	--	80%	-	-	-	4--10.4--D4Rat7	8--111.5--Tpm1										
1390569_at	18	*Cndp1*	-	86%	-	-				**18--106.3--D18Rat5**	1--144.1--D1Cebr21s2	6--52.8--D6Rat132						
1368281_at	19	*Dpep1*	86%	-	-	-	**19--113.4--D19Cebrp150s1**	5--71.3--D5Rat149	12--7.1--D12Ntr2									
1370005_at	19	*Cyb5b*	-	-	-	73%										1--130.7--D1Utr9	7--42.8--D7Rat107	
1370363_at	19	*Ces3*	-	88%	-	-				**19--6.7--D19Rat56**	1--143.4--D1Arb17	16--25.9--D16Rat28						
1368086_a_at	20	*LOC497745*	-	76%	-	-				**20--25.3--D20Ucsf1**	10--22.2--D10Cebrp207s1							
1369667_at	20	*Vps52*	-	-	-	92%										**20--0.8--Tnfa**	18--105.5--D18Cebr51s1	
1370428_x_at	20	*RT1-A1*	96%	95%	98%	-	**20--0.8--Tnfa**	11--91.7--D11Rat43		**20--0.8--Tnfa**	18--105.5--D18Cebr51s1		**20--0.8--Tnfa**	19--16.6--D19Utr4				
1371033_at	20	*RT1-Bb*	97%	98%	-	-	**20--0.8--Tnfa**	1--204.9--D1Rat304	1--217.6--D1Utr5	**20--0.8--Tnfa**	19--0.8--D19Rat19	19--15.2--D19Utr3						
1371985_a_at	20	*Bat5*	-	-	-	93%										**20--0.8--Tnfa**	11--91.7--D11Rat43	
1374429_at	20	--	-	-	95%	-							**20--0.8--Tnfa**	6--117.4--D6Cebrp91s1				
1388202_at	20	*RT1-CE10*	96%	-	97%	-	**20--0.8--Tnfa**	11--91.7--D11Rat43					**20--0.8--Tnfa**	11--91.7--D11Rat43				
1388255_x_at	20	*RT1-CE5*	-	79%	-	-				**20--0.8--Tnfa**	18--107.7--D18Utr2							
1394386_s_at	20	*Vps52*	90%	-	-	-	**20--0.8--Tnfa**	1--204.9--D1Rat304	1--218.6--D1Arb25									
1367712_at	X	*Timp1*	-	-	92%	-							3--47.8--D3Cebrp1038s1	3--86.2--D3Cebr9s1				
