# Supplementary material for: New Insights into the Genetic Control of Gene Expression using a Bayesian Multi-tissue Approach
Source: PLoS Comput Biol. 2010 Apr 8;6(4):e1000737. doi: 10.1371/journal.pcbi.1000737 (PMC2851562; doi:10.1371/journal.pcbi.1000737)
Supplement: Table S5 — eQTLs that were detected in common to all tissues by the SBR model (FDR <5%). (0.09 MB PDF) [file pcbi.1000737.s013.pdf]

Table S5. eQTLs that were detected in common to all tissues by the SBR model (FDR <5%). The results from the Bayesian modeling are from the best model visited (see Materials and Methods). For the probe sets, we determined which eQTLs were regulated in *cis* or in *trans* by defining *cis*-eQTLs as those with a peak of linkage within 10 Mbp of the physical location of the probe set. We also report the R^2 for the best SBR model visited within each tissue. For comparison with the single tissue SBR moldel, we report the results from the QTL Reaper, SSM, SBMR and Hotelling's T^2-test analyses using the same FDR cut-off of 5%, and use "Y" or "N" to indicate whether the shared eQTL was commonly detected or missed, respectively.

Probe identifier	Gene symbol	Gene name	Transcript chromosome	Transcript physical position (Mb)	Genetic marker at peak of linkage	Marker chromosome	Marker physical position (Mb)	cis/trans	Marker/transcript distance (Mb)	R^2 for the best model				SSM (FDR < 5%)	eQTL Reaper (FDR < 5%)	SBMR (FDR < 5%)	Hotelling's T^2 test (FDR < 5%)
										FAT	KIDNEY	ADRENAL	HEART				
1376249_at	Fuca2	plasma alpha-L-fucosidase precursor	1	8.30	D1Rat327	1	8.21	cis	0.09	73%	86%	88%	89%	Y	Y	Y	Y
1389650_at	--	--	1	135.87	D1Rat42	1	135.50	cis	0.37	83%	81%	63%	88%	Y	Y	Y	Y
1376780_at	RGD1310022	similar to RIKEN cDNA 2610204K14	1	137.98	D1Cebr103s1	1	135.50	cis	2.49	91%	94%	74%	94%	N	N	Y	Y
1370334_at	Plekhb1	pleckstrin homology domain containing, family B (evectins) member 1	1	158.09	D1Rat47	1	158.59	cis	0.50	54%	57%	50%	66%	N	N	N	Y
1374907_at	--	--	1	158.32	D1Rat47	1	158.59	cis	0.27	90%	88%	55%	52%	N	N		Y
1375664_at	--	--	1	182.05	Scnn1g	1	175.73	cis	6.33	87%	94%	96%	93%	Y	Y	Y	Y
1372846_at	Cybasc3	cytochrome b, ascorbate dependent 3	1	213.07	D1Rat293	1	213.04	cis	0.03	69%	78%	96%	75%	Y	Y	Y	Y
1374960_at	RGD1564887_predicted	similar to 9130011E15Rik protein (predicted)	1	251.07	D1Rat81	1	250.38	cis	0.69	94%	67%	65%	57%	Y	N	Y	Y
1370114_a_at	LOC497750	phosphatidylinositol 3-kinase	2	32.61	D2Rat94	2	31.10	cis	1.50	94%	65%	57%	64%	N	N	N	Y
1371776_at	Pik3r1	phosphatidylinositol 3-kinase, regulatory subunit, polypeptide 1	2	32.61	D2Rat94	2	31.10	cis	1.51	94%	94%	94%	92%	N	N	Y	Y
1373389_at	Acad9	acyl-CoA dehydrogenase 9	2	122.57	D2Mit7	2	114.72	cis	7.86	82%	73%	85%	81%	Y	Y	Y	N
1374006_at	Kat3	kynurenine aminotransferase III	2	240.85	D2Rat66	2	241.76	cis	0.91	77%	78%	71%	89%	Y	Y	Y	Y
1388912_at	Rexo4	XPMC2 prevents mitotic catastrophe 2 homolog	3	5.85	D3Cebr204s4	3	5.00	cis	0.85	90%	92%	88%	86%	Y	Y	Y	Y
1389816_at	Endog	endonuclease G	3	9.19	D3Cebr204s4	3	5.00	cis	4.19	78%	92%	60%	92%	Y	Y	Y	Y
1373537_at	--	--	3	10.09	D3Cebr26s1	3	10.21	cis	0.12	75%	83%	53%	61%	N	N	Y	Y
1375687_at	Rab14	Ras-related protein Rab-14	3	14.24	D3Rat53	3	13.01	cis	1.24	85%	79%	81%	84%	Y	Y	Y	Y
1376796_at	Rab14	Ras-related protein Rab-14	3	14.24	D3Rat53	3	13.01	cis	1.24	79%	71%	99%	84%	Y	N	Y	Y
1385314_at	Rab14	Ras-related protein Rab-14	3	14.24	D3Rat53	3	13.01	cis	1.24	89%	81%	91%	95%	Y	Y	Y	Y
1375676_at	--	--	3	95.34	D3Rat166	3	101.26	cis	5.92	89%	86%	89%	77%	Y	Y	Y	Y
1374921_at	Rtel1	Rtel1 protein	3	170.50	Edn3	3	166.49	cis	4.00	95%	91%	90%	82%	Y	Y	Y	Y
1367689_a_at	Cd36	Cd36 antigen	4	13.51	Cd36	4	13.51	cis	0.00	76%	81%	70%	71%	Y	Y	Y	Y
1386901_at	Cd36	Cd36 antigen	4	13.51	Cd36	4	13.51	cis	0.00	97%	88%	93%	88%	Y	Y	Y	Y
1375724_at	Glcci1	glucocorticoid induced transcript 1	4	33.66	D4Utr3	4	34.41	cis	0.75	76%	63%	71%	78%	N	Y	Y	Y
1370806_at	Retsat	all-trans-retinol 13,14-reductase precursor	4	106.10	D4Rat35	4	91.58	trans	14.52	62%	85%	84%	98%	Y	Y	Y	Y
1371998_at	--	--	4	117.56	D4Rat49	4	119.52	cis	1.97	64%	68%	74%	77%	Y	N	Y	Y
1369415_at	Bhlhb2	basic helix-loop-helix family, member e40	4	144.14	D4Rat240	4	148.25	cis	4.11	50%	73%	64%	73%	N	N	Y	Y
1390284_at	Ccdc77	coiled-coil domain containing 77	4	156.71	D4Utr4	4	160.25	cis	3.54	78%	67%	74%	54%	N	N	Y	Y
1373440_at	--	--	5	49.10	D5Rat6	5	52.53	cis	3.44	63%	70%	74%	65%	Y	Y	Y	Y
1374527_at	Echdc2	enoyl Coenzyme A hydratase domain containing 2	5	129.28	D5Rat79	5	156.50	trans	27.22	68%	63%	69%	62%	N	N	Y	Y
1371960_at	--	--	5	151.06	D5Rat38	5	149.77	cis	1.30	94%	66%	92%	96%	N	N	Y	Y
1372475_at	Pink1	PTEN induced putative kinase 1	5	157.09	D8Mit3	8	45.30	trans	--	85%	91%	88%	97%	N	N	N	N
1392720_at	--	--	7	13.11	D7Mit17	7	13.08	cis	0.04	80%	76%	68%	61%	Y	N	Y	Y
1382778_at	Dusp6	dual specificity phosphatase 6	7	36.90	D7Utr1	7	23.79	trans	13.10	92%	52%	54%	94%	N	N	Y	Y
1384717_at	Ddef1_predicted	130 kDa ARF1 GTPase- activating protein	7	101.49	D7Rat17	7	100.29	cis	1.19	94%	93%	88%	85%	Y	Y	Y	N
1386941_at	Plec1	plectin 1	7	114.22	D7Cebr77s1	7	114.49	cis	0.27	60%	53%	59%	55%	N	N	Y	Y
1376091_at	Adsl	adenylosuccinate lyase	7	119.24	D7Rat129	7	119.20	cis	0.04	52%	63%	54%	78%	N	N	Y	Y
1372805_at	--	--	8	11.15	D8Utr3	8	8.94	cis	2.21	58%	72%	73%	74%	Y	N	Y	Y
1380286_x_at	RGD1311723	similar to KIAA1731 protein	8	12.11	D8Utr3	8	8.94	cis	3.17	82%	87%	80%	75%	Y	Y	Y	Y
1398460_at	RGD1311723_predicted	similar to KIAA1731 protein (predicted)	8	12.12	D8Rat56	8	8.30	cis	3.82	95%	77%	76%	72%	Y	Y	Y	Y
1377501_at	Zfp75	zinc finger protein 75	8	18.08	D8Rat68	8	19.46	cis	1.38	79%	66%	64%	72%	Y	N	Y	Y
1388366_at	Mrpl4	mitochondrial ribosomal protein L4	8	20.03	D8Rat68	8	19.46	cis	0.57	94%	97%	96%	90%	Y	Y	Y	Y
1387366_at	Ilf3	Interleukin enhancer-binding factor 3	8	20.50	D8Rat68	8	19.46	cis	1.03	93%	96%	96%	97%	Y	Y	Y	Y
1374583_at	Dcps	decapping enzyme, scavenger	8	34.90	Es6	8	32.40	cis	2.50	97%	97%	98%	98%	Y	Y	Y	N
1374485_at	--	--	8	45.98	Grik4	8	45.77	cis	0.21	45%	52%	72%	48%	N	N	Y	Y
1371442_at	Hyou1	hypoxia up-regulated protein 1 precursor	8	47.35	D8Cebr81s4	8	40.26	cis	7.09	64%	74%	95%	90%	N	Y	Y	Y
1377452_at	Clec3b	C-type lectin domain family 3, member B	8	127.91	D8Cebr16s5	8	124.95	cis	2.96	88%	83%	53%	83%	N	N	Y	Y
1387376_at	Aox1	aldehyde oxidase	9	56.73	D9Rat93	9	59.61	cis	2.88	79%	91%	60%	72%	Y	N	Y	Y
1370176_at	Trak2	trafficking kinesin-binding protein 2	9	57.48	D9Rat19	9	59.61	cis	2.13	85%	57%	74%	94%	N	N	Y	Y
1374196_at	Lancl1	lanC-like protein 1	9	65.81	Cryga	9	65.28	cis	0.54	83%	97%	71%	95%	Y	Y	Y	Y
1389334_at	Ndufa10	NADH dehydrogenase (ubiquinone) 1 alpha subcomplex 1	9	91.66	D9Rat5	9	85.37	cis	6.29	95%	49%	51%	50%	N	N	Y	Y
1370705_at	Zfp597	zinc finger protein HIT-4	10	11.92	D10Utr3	10	9.09	cis	2.83	62%	85%	96%	59%	Y	N	Y	Y
1398910_at	Stub1	STIP1 homology and U-box containing protein 1	10	15.10	D10Mit6	10	14.72	cis	0.38	55%	59%	45%	65%	N	N	Y	Y
1375519_at	LOC287167	globin, alpha	10	15.56	D10Mit6	10	14.72	cis	0.84	88%	51%	60%	81%	N	N	Y	Y
1373417_at	Plscr3	phospholipid scramblase 3	10	56.69	D10Wox13	10	55.87	cis	0.82	79%	68%	73%	63%	Y	N	Y	Y
1370930_at	--	--	10	57.62	D10Wox13	10	55.87	cis	1.76	78%	79%	75%	70%	Y	Y	Y	Y
1374888_at	Ccdc49	coiled-coil domain containing 49 (predicted)	10	86.49	D10Rat145	10	84.92	cis	1.57	93%	81%	66%	80%	Y	Y	Y	N
1379896_at	Ddx42	ATP-dependent RNA helicase DDX42	10	95.63	D10Utr2	10	96.24	cis	0.62	69%	79%	75%	66%	Y	Y	Y	N
1389935_at	RGD1309310_predicted	similar to mKIAA0195 protein	10	105.90	D10Cebr39s1	10	76.77	trans	29.13	51%	67%	83%	81%	N	N	Y	Y
1390364_at	--	--	11	32.73	D11Mit2	11	30.89	cis	1.84	85%	70%	66%	75%	Y	Y	Y	Y
1372438_at	Nit2	nitrilase family, member 2	11	44.29	D11Rat7	11	46.56	cis	2.26	67%	70%	63%	93%	Y	Y	Y	Y
1376840_at	--	--	12	9.67	D12Cebrp97s9	12	5.40	cis	4.27	75%	93%	84%	86%	Y	Y	Y	Y
1377244_at	Zfp95	zinc finger with KRAB and SCAN domains 5	12	9.70	D12Cebrp97s9	12	5.40	cis	4.30	76%	68%	47%	80%	N	N	Y	Y
1376550_at	--	--	12	13.17	D12Mit5	12	12.80	cis	0.37	93%	73%	61%	65%	Y	Y	Y	Y
1371905_at	MGC94190	similar to 0610007L01Rik protein	12	27.48	D12Mit7	12	17.76	cis	9.72	79%	84%	80%	72%	Y	Y	Y	Y
1373682_at	DDX51	DEAD (Asp-Glu-Ala-Asp) box polypeptide 51	12	46.16	D12Rat20	12	44.12	cis	2.04	63%	79%	58%	72%	Y	Y	Y	Y
1370815_at	Nefh	Neurofilament heavy polypeptide	14	85.60	D14Utr1	14	77.70	cis	7.89	72%	73%	54%	73%	N	N	Y	Y
1388654_at	RGD:1309297	mitochondrial ribosomal protein L52 (predicted)	15	32.49	D15Rat6	15	32.65	cis	0.16	97%	92%	99%	96%	Y	Y	Y	Y
1390435_at	RGD1307583_predicted	LOC361111 (predicted)	16	6.56	D16Mit2	16	4.30	cis	2.25	72%	56%	64%	72%	Y	Y	Y	Y
1376732_at	Calr3	calreticulin 3	16	17.83	D16Utr1	16	16.19	cis	1.63	66%	59%	79%	89%	Y	Y	Y	Y
1388508_at	Ap1m1	adaptor-related protein complex 1, mu 1 subunit	16	18.03	D16Utr1	16	16.19	cis	1.83	92%	84%	92%	93%	Y	Y	Y	Y
1389793_at	--	--	16	20.00	D16Utr1	16	16.19	cis	3.81	89%	77%	83%	78%	Y	Y	Y	Y
1389722_at	--	--	16	62.67	D16Rat60	16	64.08	cis	1.41	75%	55%	89%	62%	N	N	Y	Y
1375315_at	AI316807	expressed sequence AI316807	16	74.19	D16Rat34	16	72.65	cis	1.53	62%	70%	79%	77%	N	N	Y	N
1388603_a_at	Hbld2	Iron-sulfur cluster assembly 1 homolog, mitochondrial precursor	17	10.82	D17Ucsf2	17	14.57	cis	3.75	70%	65%	59%	85%	N	N	Y	N
1383263_at	Ogn_predicted	osteoglycin	17	20.98	D17Utr1	17	23.79	cis	2.81	55%	50%	63%	63%	N	N	Y	Y
1376749_at	Ogn	osteoglycin	17	20.99	D17Utr1	17	23.79	cis	2.81	66%	57%	65%	60%	N	N	Y	Y
1388617_at	Bphl	biphenyl hydrolase-like	17	37.14	D17Rat144	17	37.97	cis	0.83	88%	74%	83%	87%	Y	Y	Y	Y
1374959_at	--	NAD(P)H dehydrogenase, quinone 2	17	37.27	D17Mit2	17	38.36	cis	1.09	79%	56%	94%	52%	N	N	Y	Y
1368225_at	Exoc2	exocyst complex component 2	17	40.04	D17Mit2	17	38.36	cis	1.68	76%	71%	88%	86%	Y	N	Y	Y
1388656_at		--	18	28.43	D18Rat99	18	31.68	cis	3.25	53%	59%	65%	49%	N	N	Y	Y
1373964_at	RGD1305487_predicted	similar to CG9147-PB	18	52.72	D18Rat57	18	54.59	cis	1.87	57%	65%	52%	57%	N	N	N	Y
1374392_at	Csnk1a1	casein kinase 1, alpha 1	18	57.57	D18Rat55	18	54.93	cis	2.64	72%	55%	57%	68%	Y	N	Y	Y
1375343_at	--	--	18	66.36	D18Rat13	18	67.87	cis	1.51	91%	97%	93%	96%	Y	Y	Y	Y
1388630_at	--	--	18	67.16	D18Rat13	18	67.87	cis	0.71	81%	60%	79%	89%	Y	Y	Y	Y
1371658_at	Cox4nb	COX4 neighbor	19	51.02	D19Cebr204s28	19	51.03	cis	0.01	49%	68%	62%	72%	N	N	Y	Y
1388202_at	RT1-CE10	RT1 class I, CE10	20	3.56	Tnfa	20	4.74	cis	1.18	96%	77%	97%	67%	N	Y	Y	Y
1371033_at	RT1-Bb	RT1 class II histocompatibility antigen, B-1 beta chain precursor	20	4.73	Tnfa	20	4.74	cis	0.01	97%	98%	81%	75%	Y	Y	Y	N
1370428_x_at	RT1-A1	RT1 class Ia, locus A2	20	5.02	Tnfa	20	4.74	cis	0.28	96%	95%	98%	77%	Y	Y	Y	N
1394386_s_at	Vps52	Vacuolar protein sorting-associated protein 52 homolog	20	5.07	Tnfa	20	4.74	cis	0.33	90%	76%	81%	79%	Y	Y	Y	N
1369667_at	Vps52	Vacuolar protein sorting-associated protein 52 homolog	20	5.08	Tnfa	20	4.74	cis	0.34	73%	77%	76%	92%	Y	Y	Y	N
1374429_at	--	--	20	7.82	Tnfa	20	4.74	cis	3.08	68%	72%	95%	77%	Y	Y	Y	Y
1368597_at	Snf1lk	Serine/threonine-protein kinase SNF1-like kinase 1	20	10.29	D20Rat4	20	12.14	cis	1.86	70%	66%	59%	77%	Y	N	Y	N
1374916_at	Pwp2h	periodic tryptophan protein homolog	20	10.85	D20Utr2	20	10.72	cis	0.13	81%	74%	72%	72%	Y	Y	Y	Y
