# Supplementary material for: New Insights into the Genetic Control of Gene Expression using a Bayesian Multi-tissue Approach
Source: PLoS Comput Biol. 2010 Apr 8;6(4):e1000737. doi: 10.1371/journal.pcbi.1000737 (PMC2851562; doi:10.1371/journal.pcbi.1000737)
Supplement: Table S6 — Cis-regulated transcripts found by both SBMR and the Hotelling's T2-test at 5% FDR. (0.09 MB PDF) [file pcbi.1000737.s014.pdf]

Table S6. *Cis*-regulated transcripts found by both SBMR and the Hotelling's T^2-test at 5% FDR. For each probeset, we report only the common *cis*-eQTLs but not the additional *trans*-eQTLs that were identified by either SBMR or Hotelling's T^2-test. For each probe set, we determined which eQTLs were regulated in *cis* or in *trans* by defining *cis*-eQTLs as those with a peak of linkage within 10 Mbp of the physical location of the probe set.

Probe identifier	Gene symbol	Gene name	Transcript chromosome	Transcript physical position (Mb)	Genetic marker at peak of linkage	Marker chromosome	Marker physical position (Mb)	Marker/transcript distance (Mb)
1371324_at	--	--	1	7.75	*D1Rat327*	1	8.21	0.46
1372312_at	*Ltv1*	LTV1 homolog	1	7.97	*D1Rat327*	1	8.21	0.24
1376249_at	*Fuca2*	Plasma alpha-L-fucosidase precursor	1	8.30	*D1Rat327*	1	8.21	0.09
1369956_at	*Ifngr1*	interferon gamma receptor 1	1	14.86	*D1Rat7*	1	16.00	1.14
1374851_at	--	--	1	83.50	*D1Rat24*	1	76.81	6.69
1373781_a_at	*Sbsn*	Suprabasin Precursor	1	85.80	*D1Rat212*	1	86.59	0.79
1399157_at	*NP_001100977.1*	similar to NNX3 (predicted)	1	90.43	*D1Rat27*	1	90.36	0.07
1389673_at	--	--	1	101.40	*D1Rat30*	1	100.62	0.78
1387812_at	--	--	1	120.48	*D1Rat30*	1	100.62	4.35
1372658_at	--	--	1	122.50	*D1Utr3*	1	123.04	0.54
1389650_at	--	--	1	135.87	*D1Rat42*	1	135.50	0.37
1376780_at	*RGD1310022*	similar to RIKEN cDNA 2610204K14	1	137.99	*D1Cebr103s1*	1	139.83	1.84
1373152_at	*Prss23*	Serine protease 23 precursor	1	146.09	*C*	1	140.72	5.37
1368669_at	*Ucp2*	Mitochondrial uncoupling protein 2 (UCP 2)	1	157.93	*D1Rat47*	1	158.59	0.66
1371085_at	*Ascl3*	Putative bHLH transcription factor	1	167.35	*D1Rat55*	1	170.61	3.26
1377407_at	--	--	1	177.79	*D1Cebr7s3*	1	178.17	0.38
1388567_at	*LOC682025*	THUMP domain containing 1	1	178.00	*D1Cebr7s3*	1	178.17	0.17
1389300_at	--	--	1	178.23	*D1Cebr7s3*	1	178.17	0.06
1375664_at	--	--	1	182.05	*Scnn1g*	1	180.79	1.26
1374017_at	--	--	1	187.30	*Myl2*	1	186.62	0.68
1373829_at	--	--	1	189.55	*D1Rat69*	1	199.26	9.71
1368311_at	*Mgmt*	Methylated-DNA--protein-cysteine methyltransferase	1	196.87	*D1Arb22*	1	198.32	1.45
1372846_at	*Cybasc3*	cytochrome b, ascorbate dependent 3	1	213.08	*D1Rat293*	1	213.04	0.04
1369098_at	*Vldlr*	Very low-density lipoprotein receptor precursor (VLDL receptor)	1	230.70	*D1Mit34*	1	231.13	0.43
1388491_at	*NP_001099842.1*	membrane-associated ring finger (C3HC4) 5	1	241.50	*D1Rat304*	1	242.35	0.85
1369626_at	*Ide*	Insulin-degrading enzyme	1	241.65	*D1Rat304*	1	242.35	0.70
1375922_at	--	--	1	247.09	*D1Rat235*	1	248.22	1.13
1398473_at	*Bloc1s2*	Biogenesis of lysosome-related organelles complex-1 subunit 2	1	249.09	*D1Rat81*	1	250.38	1.29
1374960_at	*RGD1564887*	similar to 9130011E15Rik protein (predicted)	1	251.11	*D1Rat81*	1	250.38	0.73
1372093_at	*Mxi1*	MAX-interacting protein 1	1	259.26	*D1Rat225*	1	261.39	2.13
1390466_at	--	--	2	31.50	*D2Rat94*	2	31.10	0.40
1371776_at	*Pik3r1*	Phosphatidylinositol 3-kinase, regulatory subunit, polypeptide 1	2	32.61	*D2Rat94*	2	31.10	1.51
1374586_at	--	--	2	45.71	*D2Rat202*	2	51.75	6.04
1387144_at	*Itga1*	Integrin alpha-1 precursor	2	47.21	*D2Rat201*	2	49.62	2.41
1376944_at	--	--	2	59.69	*D2Mit4*	2	63.35	3.66
1373082_at	--	--	2	96.52	*D2Utr4*	2	94.66	1.86
1389580_at	--	--	2	105.26	*D2Mit18*	2	108.10	2.84
1398387_at	*MGC72614*	Uncharacterized protein C4orf18 homolog	2	171.43	*Hsp27rs*	2	174.70	3.27
1386890_at	*S100a10*	Protein S100-A10	2	186.65	*D2Cebr104s1*	2	180.72	5.93
1367673_at	*Selenbp1*	Selenium-binding protein 1	2	189.85	*D2Cebr104s1*	2	180.72	9.13
1367986_at	*Ptgfrn*	Prostaglandin F2 receptor negative regulator precursor	2	196.16	*D2Cebr10s6*	2	197.22	1.06
1368988_at	*Casq2*	Calsequestrin-2 precursor	2	197.24	*D2Cebr10s6*	2	197.22	0.02
1386985_at	*Gstm1*	Glutathione S-transferase Mu 1	2	203.58	*D2Rat157*	2	206.37	2.79
1380404_at	--	--	2	213.17	*D2Rat236*	2	204.10	9.07
1373610_at	--	--	2	220.10	*D2Cebr4s8*	2	220.39	0.29
1376723_a_at	--	--	2	220.26	*D2Cebr4s8*	2	220.39	0.13
1374006_at	*1374006_at*	kynurenine aminotransferase III	2	240.85	*D2Rat66*	2	241.76	0.91
1368637_at	*Card9*	Caspase recruitment domain-containing protein 9	3	4.53	*D3Cebr83s1*	3	2.50	2.03
1389973_a_at	*Surf4*	Surfeit locus protein 1	3	5.83	*D3Ucsf1*	3	7.50	1.67
1388912_at	*Rexo4*	XPMC2 prevents mitotic catastrophe 2 homolog	3	5.86	*D3Cebr204s4*	3	5.00	0.86
1370573_at	*Sardh*	Sarcosine dehydrogenase, mitochondrial precursor	3	6.14	*D3Cebr204s4*	3	5.00	1.14
1389816_at	*Endog*	endonuclease G	3	9.19	*D3Cebr204s4*	3	5.00	4.19
1373782_a_at	--	--	3	9.19	*D3Cebr204s4*	3	5.00	4.19
1373537_at	--	--	3	10.09	*D3Cebr26s1*	3	9.36	0.73
1370964_at	*Ass*	Argininosuccinate synthase	3	10.62	*D3Cebr26s1*	3	9.36	1.26
1375687_at	*Rab14*	Ras-related protein Rab-14	3	14.25	*D3Rat53*	3	11.61	2.64
1376796_at	*Rab14*	Ras-related protein Rab-14	3	14.25	*D3Rat53*	3	11.61	2.64
1368618_at	*Grb14*	Growth factor receptor-bound protein 14	3	46.95	*D3Utr8*	3	48.28	1.32
1390501_at	--	--	3	55.84	*D3Rat180*	3	58.66	2.82
1368051_at	*Hsd17b12*	Estradiol 17-beta-dehydrogenase 12	3	78.56	*D3Utr4*	3	76.82	1.74
1375676_at	--	--	3	95.34	*D3Rat166*	3	101.26	5.92
1370232_at	*Ivd*	Isovaleryl-CoA dehydrogenase, mitochondrial precursor	3	105.40	*D3Utr6*	3	109.86	4.46
1370194_at	--	--	3	107.36	*D3Utr6*	3	109.86	2.50
1370195_at	--	--	3	107.36	*D3Utr6*	3	109.86	2.50
1389716_at	--	--	3	107.39	*D3Utr6*	3	109.86	2.47
1369635_at	*Q6TUH3_RAT*	LRRGT00071	3	109.05	*D3Utr6*	3	109.86	0.81
1375362_at	--	--	3	114.44	*D3Rat257*	3	115.93	1.49
1370156_at	*Prnp*	Major prion protein precursor	3	119.69	*D3Rat159*	3	119.34	0.35
1390717_at	*RGD1311037*	Cardiolipin synthetase	3	120.67	*D3Rat159*	3	119.34	1.33
1387170_at	*Csnk2a1*	Casein kinase II subunit alpha	3	142.61	*D3Mit3*	3	136.31	6.30
1373749_at	*Snta1*	Snta1 protein	3	144.87	*D3Mit3*	3	136.31	8.56
1388178_at	*Ncoa3*	Nuclear receptor coactivator 3	3	157.20	*Svp1*	3	157.49	0.29
1387906_a_at	--	--	3	165.22	*D3Rat132*	3	164.97	0.25
1367689_a_at	*Cd36*	Cd36 antigen	4	13.55	*Cd36*	4	13.51	0.04
1386901_at	*Cd36*	Cd36 antigen	4	13.55	*Cd36*	4	13.51	0.04
1370902_at	*Akr1b8*	aldo-keto reductase family 1, member B8	4	61.73	*Lep*	4	56.19	5.54
1374785_at	--	--	4	66.25	*D4Rat102*	4	66.50	0.25
1368947_at	*Gadd45a*	Growth arrest and DNA-damage-inducible protein	4	96.65	*D4Rat35*	4	91.58	5.07
1371998_at	--	--	4	117.56	*D4Rat49*	4	119.72	2.16
1377155_at	--	--	4	120.05	*D4Rat49*	4	119.72	0.33
1368208_at	*RGD621605*	Probable N-acetyltransferase CML6	4	120.08	*D4Rat49*	4	119.72	0.36
1369415_at	*Bhlhb2*	basic helix-loop-helix family, member e40	4	144.15	*D4Rat240*	4	148.25	4.10
1390284_at	*1390284_at*	coiled-coil domain containing 77	4	156.71	*D4Utr4*	4	161.22	4.51
1389553_at	--	--	4	159.47	*D4Rat202*	4	159.36	0.11
1373510_at	--	--	4	161.34	*D4Rat66*	4	161.27	0.07
1376200_at	--	--	4	186.58	*D4Rat140*	4	182.99	3.59
1373143_at	*RGD1309621*	RGD1309621 protein (Fragment)	4	186.79	*D4Rat140*	4	182.99	3.80
1367949_at	*Penk1*	Proenkephalin A precursor	5	17.51	*D5Rat188*	5	18.49	0.98
1372700_at	--	--	5	25.01	*D5Rat126*	5	24.79	0.22
1368910_at	*Ppm2c*	Pyruvate dehydrogenase	5	26.24	*D5Rat126*	5	24.79	1.45
1373440_at	--	--	5	49.10	*D5Rat6*	5	52.53	3.43
1373427_at	*NP_001100111.1*	Ras-related GTP binding D	5	49.30	*D5Rat6*	5	52.53	3.23
1376628_at	*NP_001101400.1*	zinc finger protein 189	5	66.28	*D5Utr1*	5	64.57	1.71
1376537_at	--	--	5	75.11	*D5Rat228*	5	74.57	0.54
1374527_at	*Echdc2*	enoyl Coenzyme A hydratase domain containing 2	5	129.28	*D5Rat158*	5	125.26	4.02
1371960_at	--	--	5	151.06	*D5Rat38*	5	149.77	1.29
1380433_at	--	--	5	153.27	*D5Rat93*	5	154.02	0.75
1374554_at	*1110049F12Rik*	UPF0424 protein C1orf128 homolog	5	154.77	*D5Rat93*	5	154.02	0.75
1368440_at	*Slc3a1*	Neutral and basic amino acid transport protein rBAT	6	8.45	*D6Cebrp424s2*	6	15.16	6.71
1374004_at	*RGD1310143*	Prolyl endopeptidase-like	6	8.45	*D6Cep8*	6	15.85	7.40
1369973_at	*Xdh*	Xanthine dehydrogenase/oxidase	6	21.59	*D6Rat147*	6	21.90	0.31
1375532_at	--	--	6	42.78	*D6Rat28*	6	43.41	0.63
1377329_at	--	--	6	54.84	*D6Cebrp97s14*	6	56.36	1.52
1370258_at	*Bzw2*	Basic leucine zipper and W2 domain-containing protein 2	6	54.88	*D6Cebrp97s14*	6	56.36	1.47
1379281_at	*Sostdc1*	Sclerostin domain-containing protein 1 precursor	6	55.07	*D6Cebrp97s14*	6	56.36	1.28
1372681_at	--	--	6	64.25	*D6Cebrp97s14*	6	56.36	7.89

Probe identifier	Gene symbol	Gene name	Transcript chromosome	Transcript physical position (Mb)	Genetic marker at peak of linkage	Marker chromosome	Marker physical position (Mb)	Marker/transcript distance (Mb)
1375450_at	*Sdccag1*	Serologically defined colon cancer antigen 1 homolog	6	91.28	*D6Cebr36s1*	6	97.87	6.59
1376268_at	--	--	6	91.34	*D6Cebr36s1*	6	97.87	6.53
1368859_at	*Ppm1a*	Protein phosphatase 1A	6	95.20	*D6Cebr36s1*	6	97.87	2.67
1370829_at	*Fntb*	Protein farnesyltransferase subunit beta	6	99.51	*D6Rat87*	6	98.75	0.76
1392720_at	--	--	7	13.11	*D7Mit17*	7	13.08	0.03
1382778_at	*Dusp6*	dual specificity phosphatase 6	7	36.90	*D7Utr1*	7	32.36	4.54
1377064_at	*Dusp6*	Dual specificity protein phosphatase 6	7	36.90	*D7Utr1*	7	32.36	4.54
1370609_a_at	*Slc16a7*	Monocarboxylate transporter 2 (MCT 2)	7	65.29	*D7Mit6*	7	61.84	3.45
1368536_at	*Enpp2*	Ectonucleotide pyrophosphatase/phosphodiesterase family member 2 Precursor	7	91.38	*D7Rat112*	7	90.26	1.12
1388347_at	*LOC362934*	similar to lymphocyte antigen 6 complex	7	113.16	*Cyp11b2*	7	113.05	0.11
1386941_at	*Plec1*	plectin 1	7	114.24	*D7Cebr77s1*	7	115.04	0.80
1368091_at	*Oplah*	5-oxoprolinase	7	114.34	*D7Rat131*	7	111.92	2.42
1371725_at	--	--	7	115.68	*D7Rat131*	7	111.92	3.76
1370881_at	*Tst*	Thiosulfate sulfurtransferase	7	116.36	*D7Cebr77s1*	7	115.04	1.32
1374915_at	--	--	7	116.87	*D7Rat129*	7	119.20	2.33
1367628_at	*Lgals1*	Galectin-1 (Lectin galactoside-binding soluble 1)	7	116.90	*D7Rat131*	7	111.92	4.98
1376091_at	*Adsl*	adenylosuccinate lyase	7	119.25	*D7Rat129*	7	119.20	0.05
1383188_at	--	--	7	123.81	*D7Mit2*	7	123.67	0.14
1372624_at	--	--	7	134.62	*D7Rat4*	7	134.69	0.07
1373198_at	*RGD1311098*	similar to RIKEN cDNA 2810451A06	7	137.88	*D7Utr4*	7	137.48	0.40
1372734_at	*Smagp*	small cell adhesion glycoprotein	7	139.40	*D7Cebr24s1*	7	137.90	1.50
1387819_at	*Ela1*	Elastase-1 precursor	7	139.45	*D7Cebr205s1*	7	141.00	1.55
1372805_at	--	--	8	11.15	*D8Rat56*	8	8.29	2.86
1398460_at	*RGD1311723*	similar to KIAA1731 protein	8	12.12	*D8Rat56*	8	8.29	3.83
1380286_x_at	*RGD1311723*	similar to KIAA1731 protein	8	12.12	*D8Utr3*	8	9.17	2.95
1377501_at	*Zfp75*	zinc finger protein 75	8	18.08	*D8Rat68*	8	19.46	1.38
1388366_at	*Mrpl4*	mitochondrial ribosomal protein L4	8	20.03	*D8Rat68*	8	19.46	0.57
1387366_at	*Ilf3*	Interleukin enhancer-binding factor 3	8	20.51	*D8Rat68*	8	19.46	1.05
1377061_at	--	--	8	32.03	*D8Rat49*	8	32.19	0.16
1390185_at	*Dcps*	Scavenger mRNA-decapping enzyme DcpS	8	34.96	*Kcnj1*	8	32.17	2.79
1390710_x_at	*Sorl1*	LR11 (Fragment)	8	44.98	*Grik4*	8	45.83	0.85
1393933_at	*Sorl1*	LR11 (Fragment)	8	44.98	*Grik4*	8	45.83	0.85
1377457_a_at	--	--	8	44.98	*Grik4*	8	45.83	0.85
1373055_at	--	--	8	45.40	*Grik4*	8	45.83	0.43
1374485_at	--	--	8	45.98	*Grik4*	8	45.83	0.15
1370665_at	*Hyou1*	Hypoxia up-regulated protein 1 precursor	8	47.36	*Scnb2*	8	48.08	0.72
1371442_at	*Hyou1*	Hypoxia up-regulated protein 1 precursor	8	47.36	*Scnb2*	8	48.08	0.72
1382838_at	--	--	8	60.91	*Crabp1*	8	58.34	2.57
1376110_at	*Rpp25*	Ribonuclease P protein subunit p25	8	61.25	*D8Mit12*	8	58.05	3.20
1370539_at	*Rab8b*	Ras-related protein Rab-8B	8	71.23	*Tpm1*	8	71.36	0.13
1389107_at	--	--	8	76.09	*D8Utr5*	8	79.25	3.16
1382105_at	--	--	8	80.17	*D8Cebr204s21*	8	87.14	6.97
1372500_at	*Tmod3*	tropomodulin 3	8	80.32	*D8Cebr204s21*	8	87.14	6.82
1372297_at	--	--	8	83.18	*D8Cebr204s21*	8	87.14	3.96
1370228_at	*Tf*	Serotransferrin precursor	8	108.24	*D8Ucsf1*	8	108.55	0.31
1372376_at	--	--	8	115.00	*Mylc1v*	8	115.16	0.16
1374159_at	--	--	8	115.29	*Mylc1v*	8	115.16	0.13
1382997_at	--	--	8	124.16	*D8Cebr46s6*	8	118.08	6.08
1377452_at	*Clec3b*	C-type lectin domain family 3, member B	8	127.91	*D8Cebr16s5*	8	126.00	1.91
1388926_at	--	--	9	12.57	*D9Rat131*	9	14.43	1.86
1371951_at	--	--	9	42.33	*D9Rat104*	9	42.29	0.04
1369313_at	*Fhl2*	Four and a half LIM domains protein 2	9	42.39	*D9Rat104*	9	42.29	0.10
1373887_at	*Sf3b1*	Spliceosomal protein SAP155	9	53.80	*D9Rat60*	9	44.98	8.82
1387376_at	*Aox1*	Aldehyde oxidase	9	56.77	*D9Rat93*	9	55.90	0.87
1370176_at	*Trak2*	Trafficking kinesin-binding protein 2	9	57.48	*D9Rat19*	9	59.61	2.13
1384309_at	--	--	9	61.79	*D9Rat156*	9	61.49	0.30
1374196_at	*Lancl1*	LanC-like protein 1	9	65.83	*Cryga*	9	63.87	1.96
1368127_at	*Neu2*	Sialidase-2	9	86.56	*D9Rat4*	9	90.90	4.34
1392702_at	--	--	9	112.81	*D9Rat1*	9	111.07	1.74
1370705_at	*Zfp597*	zinc finger protein HIT-4	10	11.93	*D10Utr3*	10	10.13	1.80
1371916_at	--	--	10	14.00	*D10Cebr27s2*	10	13.24	0.76
1398910_at	*Stub1*	STIP1 homology and U-box containing protein 1	10	15.10	*D10Mit6*	10	14.72	0.38
1375519_at	*LOC287167*	globin, alpha	10	15.56	*D10Mit6*	10	14.72	0.84
1368574_at	--	--	10	28.95	*D10Rat71*	10	29.54	0.59
1375540_at	--	--	10	37.50	*D10Mit4*	10	36.65	0.85
1371803_at	*Gm2a*	GM2 ganglioside activator protein	10	40.51	*D10Rat215*	10	38.40	2.11
1367562_at	*Sparc*	SPARC precursor	10	40.83	*D10Rat166*	10	43.94	3.11
1390063_at	*Mfap3*	Microfibril-associated glycoprotein 3 precursor	10	43.12	*D10Rat166*	10	43.94	0.82
1377353_a_at	--	--	10	56.50	*D10Wox14*	10	56.91	0.41
1373417_at	*Plscr3*	Phospholipid scramblase 3	10	56.69	*D10Wox13*	10	55.87	0.82
1367989_at	*Slc2a4*	Solute carrier family 2	10	56.79	*D10Rat102*	10	52.52	4.27
1372064_at	*Cxcl16*	similar to chemokine (C-X-C motif) ligand 16	10	57.31	*D10Wox13*	10	55.87	1.44
1370930_at	*Kif1c*	kinesin family member 1C	10	57.62	*D10Wox13*	10	55.87	1.75
1375672_at	--	--	10	62.78	*D10Rat80*	10	61.81	0.97
1376742_at	--	--	10	85.03	*D10Rat145*	10	84.92	0.11
1374948_at	*Tmem106a*	Transmembrane protein 106A	10	90.61	*Ppy*	10	91.18	0.57
1390310_at	--	--	10	95.77	*D10Utr2*	10	95.58	0.19
1389935_at	*RGD1309310_predicted*	similar to mKIAA0195 protein	10	105.90	*D10Rat7*	10	105.90	0.00
1373438_at	--	--	10	106.65	*D10Rat7*	10	105.90	0.75
1373895_at	--	--	10	107.18	*D10Rat7*	10	105.90	1.28
1390443_at	--	--	11	17.45	*D11Rat20*	11	16.67	0.78
1371572_at	*App*	Amyloid beta A4 protein precursor	11	24.69	*D11Rat19*	11	27.01	2.32
1375696_at	*NP_001099363.1*	interferon (alpha and beta) receptor 1	11	31.48	*D11Cebr15s1*	11	32.79	1.31
1379298_at	*NP_001099363.1*	interferon (alpha and beta) receptor 1	11	31.48	*D11Cebr15s1*	11	32.79	1.31
1388686_at	*Dscr1*	Calcipressin-1	11	32.41	*D11Rat16*	11	30.81	1.60
1390364_at	--	--	11	32.73	*D11Mit2*	11	30.89	1.84
1368037_at	*Cbr1*	Carbonyl reductase	11	33.79	*D11Cebr15s1*	11	32.79	1.00
1373085_at	*NP_001100580.1*	carbonyl reductase 3	11	33.92	*D11Cebr15s1*	11	32.79	1.13
1372438_at	*Nit2*	nitrilase family, member 2	11	44.30	*D11Rat7*	11	46.56	2.26
1376840_at	--	--	12	9.67	*D12Cebrp97s9*	12	5.55	4.12
1377244_at	*Zfp95*	zinc finger with KRAB and SCAN domains 5	12	9.70	*D12Cebrp97s9*	12	5.55	4.15
1376550_at	--	--	12	13.17	*D12Mit5*	12	12.80	0.37
1386936_at	*Grifin*	Grifin	12	14.55	*Pai1*	12	20.93	6.38
1375958_at	--	--	12	19.81	*Hsp27*	12	21.90	2.09
1376688_a_at	--	--	12	20.01	*D12Rat28*	12	16.34	3.67
1377082_at	--	--	12	20.79	*D12Mit7*	12	17.76	3.03
1392941_at	--	--	12	22.42	*Hsp27*	12	21.90	0.52
1371905_at	*MGC94190*	similar to 0610007L01Rik protein	12	27.49	*D12Mit7*	12	17.76	9.73
1383321_at	--	--	12	27.59	*D12Cebr1s1*	12	30.13	2.54
1373682_at	*RGD:1309580*	DEAD (Asp-Glu-Ala-Asp) box polypeptide 51	12	46.16	*D12Rat20*	12	44.12	2.04
1373661_a_at	*Cxcr4*	C-X-C chemokine receptor type 4	13	41.31	*D13Cebr5s3*	13	45.52	4.21
1389521_at	*Ivns1abp_predicted*	influenza virus NS1A binding protein	13	66.24	*D13Cebr2s5*	13	61.84	4.40
1368304_at	*Fmo3*	Dimethylaniline monooxygenase	13	78.68	*D13Mit3*	13	78.08	0.60
1371732_at	*Dpt*	Dermatopontin Precursor	13	80.59	*D13Rat131*	13	80.77	0.18
1377807_a_at	--	--	13	81.08	*D13Rat131*	13	80.77	0.31
1387314_at	*Sult1b1*	Sulfotransferase family cytosolic 1B member 1	14	22.03	*D14Utr5*	14	17.39	4.64
1367816_at	*Hod*	Homeodomain-only protein (Odd homeobox protein 1)	14	33.38	*D14Rat36*	14	33.04	0.34
1375194_at	--	--	14	36.61	*D14Rat52*	14	37.23	0.62
1374539_at	--	--	14	38.34	*D14Cebrp136s2*	14	45.02	6.68
1382667_at	--	--	14	81.75	*D14Rat37*	14	81.45	0.30

Probe identifier	Gene symbol	Gene name	Transcript chromosome	Transcript physical position (Mb)	Genetic marker at peak of linkage	Marker chromosome	Marker physical position (Mb)	Marker/transcript distance (Mb)
1370815_at	*Nefh*	Neurofilament heavy polypeptide	14	85.60	*D14Utr1*	14	78.45	7.15
1375530_at	--	--	15	21.28	*D15Mit3*	15	20.06	1.22
1387808_at	*Slc7a7*	Y+L amino acid transporter 1	15	32.47	*D15Rat6*	15	32.65	0.18
1388654_at	*RGD:1309297*	mitochondrial ribosomal protein L52 (predicted)	15	32.49	*D15Rat6*	15	32.65	0.16
1373122_at	*Jub*	Protein ajuba	15	32.64	*D15Rat6*	15	32.65	0.01
1371963_at	*PCCA_RAT*	Propionyl-CoA carboxylase alpha chain, mitochondrial precursor	15	108.03	*D15Rat107*	15	108.40	0.37
1390435_at	*RGD1307583_predicted*	LOC361111 (predicted)	16	6.56	*D16Rat51*	16	3.48	3.08
1388909_at	*NP_001100765.1*	oxidoreductase NAD-binding domain containing 1	16	7.57	*D16Rat54*	16	11.21	3.64
1388688_at	--	--	16	13.05	*D16Rat54*	16	11.21	1.84
1376732_at	*Calr3*	calreticulin 3	16	17.84	*D16Utr1*	16	16.19	1.65
1388508_at	*Ap1m1*	adaptor-related protein complex 1, mu 1 subunit	16	18.03	*D16Utr1*	16	16.19	1.84
1377959_at	--	--	16	18.03	*D16Utr1*	16	16.19	1.84
1389793_at	--	--	16	20.01	*D16Utr1*	16	16.19	3.82
1389722_at	--	--	16	62.67	*D16Rat60*	16	64.08	1.41
1390050_at	--	--	17	10.94	*D17Ucsf2*	17	15.61	4.67
1388485_at	--	--	17	14.30	*D17Utr1*	17	23.79	9.49
1376749_at	*Ogn_predicted*	osteoglycin	17	20.99	*D17Utr1*	17	23.79	2.80
1374903_at	*Gcnt2*	glucosaminyl (N-acetyl) transferase 2, I-branching enzyme	17	29.81	*D17Rat11*	17	30.93	1.12
1375003_at	*Serpinb6a*	serine (or cysteine) proteinase inhibitor, clade B (ovalbumin), member 6	17	37.34	*D17Rat144*	17	37.97	0.63
1373672_at	*MGC94010*	similar to SPI6	17	37.79	*D17Rat144*	17	37.97	0.18
1368225_at	*Exoc2*	Exocyst complex component 2	17	40.13	*D17Rat144*	17	37.97	2.16
1374126_at	--	--	17	67.01	*D17Rat50*	17	66.99	0.02
1372869_at	*LOC291276*	Nucleolar GTP-binding protein 1	17	72.28	*D17Rat151*	17	71.58	0.70
1388986_at	--	--	17	75.58	*D17Rat151*	17	71.58	4.00
1387060_at	--	--	17	75.59	*D17Rat151*	17	71.58	4.01
1372000_at	*Net1*	neuroepithelial cell transforming gene 1	17	77.54	*D17Rat151*	17	71.58	5.96
1388195_at	--	--	17	83.03	*D17Rat62*	17	83.49	0.46
1389867_at	--	--	17	83.03	*D17Rat62*	17	83.49	0.46
1388656_at	--	--	18	28.44	*D18Cebr19s1*	18	29.50	1.06
1375267_at	*Ppic*	peptidylprolyl isomerase C	18	48.90	*D18Rat19*	18	49.65	0.75
1374392_at	*Csnk1a1*	casein kinase 1, alpha 1	18	57.57	*D18Rat55*	18	54.93	2.64
1384970_at	--	--	18	62.04	*D18Rat61*	18	61.64	0.40
1390128_at	*LOC682926*	similar to chromatin modifying protein 1B	18	63.72	*D18Rat61*	18	61.64	2.08
1368842_at	--	--	18	66.36	*D18Rat41*	18	59.35	7.01
1375343_at	--	--	18	66.36	*D18Rat13*	18	67.93	1.57
1388630_at	--	--	18	67.16	*D18Rat13*	18	67.93	0.77
1380293_at	--	--	18	67.19	*D18Rat13*	18	67.93	0.74
1372072_at	*RGD1308579*	Immediate early response 3-interacting protein 1	18	73.95	*D18Rat5*	18	77.53	3.58
1389458_at	--	--	19	10.17	*D19Rat19*	19	7.82	2.35
1390491_at	--	--	19	10.61	*D19Rat19*	19	7.82	2.79
1389774_at	--	--	19	39.98	*D19Rat71*	19	41.19	1.21
1371658_at	*Cox4nb*	COX4 neighbor	19	51.02	*D19Cebr204s28*	19	51.03	0.01
1368891_at	--	--	19	55.06	*D19Utr7*	19	53.16	1.90
1377036_at	--	--	20	0.08	*Tnfa*	20	3.66	3.58
1390562_s_at	--	--	20	0.08	*Tnfa*	20	3.66	3.58
1388236_x_at	--	--	20	0.15	*Tnfa*	20	3.66	3.51
1371171_at	--	--	20	2.84	*Tnfa*	20	3.66	0.82
1370290_at	*Tubb5*	Tubulin beta-5 chain	20	3.06	*Tnfa*	20	3.66	0.60
1388202_at	*RT1-CE10*	RT1 class I, CE10	20	3.58	*Tnfa*	20	3.66	0.08
1388203_x_at	*RT1-CE10*	RT1 class I, CE10	20	3.58	*Tnfa*	20	3.66	0.08
1371985_a_at	*Bat5*	HLA-B associated transcript 5	20	3.80	*Tnfa*	20	3.66	0.14
1377091_at	--	--	20	4.22	*Tnfa*	20	3.66	0.56
1370822_at	--	--	20	4.70	*Tnfa*	20	3.66	1.04
1370882_at	--	--	20	4.84	*Tnfa*	20	3.66	1.18
1371213_at	--	--	20	5.03	*Tnfa*	20	3.66	1.37
1369726_at	*Wdr46*	WD repeat domain 46	20	5.12	*Tnfa*	20	3.66	1.46
1374429_at	--	--	20	7.82	*Tnfa*	20	3.66	4.16
1374916_at	*Pwp2h*	periodic tryptophan protein homolog	20	10.85	*D20Utr2*	20	10.72	0.13
1374558_at	--	--	20	10.95	*D20Utr2*	20	10.72	0.23
1369640_at	*Gja1*	Gap junction alpha-1 protein	20	35.42	*D20Rat52*	20	34.71	0.71
